# New 3,4-*seco*-3,19-Dinor- and Spongian-Based Diterpenoid Lactones from the Marine Sponge *Spongia* sp.

**DOI:** 10.3390/ijms24021252

**Published:** 2023-01-08

**Authors:** Chi-Jen Tai, Chih-Hua Chao, Atallah F. Ahmed, Chia-Hung Yen, Tsong-Long Hwang, Fang-Rong Chang, Yusheng M. Huang, Jyh-Horng Sheu

**Affiliations:** 1 Department of Marine Biotechnology and Resources, National Sun Yat-sen University, Kaohsiung 80424, Taiwan; 2 National Museum of Marine Biology and Aquarium, Pingtung 944401, Taiwan; 3 School of Pharmacy, China Medical University, Taichung 40604, Taiwan; 4 Chinese Medicine Research and Development Center, China Medical University Hospital, Taichung 40604, Taiwan; 5 Department of Pharmacognosy, College of Pharmacy, King Saud University, Riyadh 11451, Saudi Arabia; 6 Department of Pharmacognosy, Faculty of Pharmacy, Mansoura University, Mansoura 35516, Egypt; 7 Graduate Institute of Natural Products, College of Pharmacy, Kaohsiung Medical University, Kaohsiung 80708, Taiwan; 8 National Natural Product Libraries and High-Throughput Screening Core Facility, Kaohsiung Medical University, Kaohsiung 80708, Taiwan; 9 Graduate Institute of Natural Products, College of Medicine, Chang Gung University, Taoyuan 333, Taiwan; 10 Research Center for Chinese Herbal Medicine, Graduate Institute of Healthy Industry Technology, College of Human Ecology, Chang Gung University of Science and Technology, Taoyuan 33303, Taiwan; 11 Department of Anesthesiology, Chang Gung Memorial Hospital, Taoyuan 333423, Taiwan; 12 Department of Marine Recreation, National Penghu University of Science and Technology, Magong 88046, Taiwan; 13 Tropical Island Sustainable Development Research Center, National Penghu University of Science and Technology, Magong 88046, Taiwan; 14 Department of Medical Research, China Medical University Hospital, China Medical University, Taichung 404333, Taiwan

**Keywords:** Red Sea sponge, *Spongia* sp., 3,4-*seco*-3,19-dinorspongian diterpenoid lactones, spongian diterpene, secodinorspongins A−D, antibacterial assay, anti-inflammatory

## Abstract

Continuing chemical investigation of the Red Sea sponge *Spongia* sp. led to the isolation of four new 3,4-*seco*-3,19-dinorspongian diterpenoid lactones, secodinorspongins A−D (**1**−**4**), along with a classical spongian diterpenoid lactone, sponginolide (**5**). The chemical structures, including the absolute configurations of these compounds, were elucidated using the extensive spectroscopic study composed of 1D and 2D NMR data analyses, and a comparison between calculated-electronic-circular-dichroism (ECD) and experimental-circular-dichroism (CD) spectra. A plausible biosynthetic pathway of **1**−**4** was also proposed. Furthermore, the cytotoxicity, antibacterial and anti-inflammatory activities of **1**−**5** were evaluated. Compound **1** was found to exhibit inhibitory activity against the growth of *Staphylococcus aureus* (*S. aureus*), and **4** and **5** exhibited suppression of superoxide-anion generation and elastase release in fMLF/CB-induced human neutrophils.

## 1. Introduction

**Figure 1:**
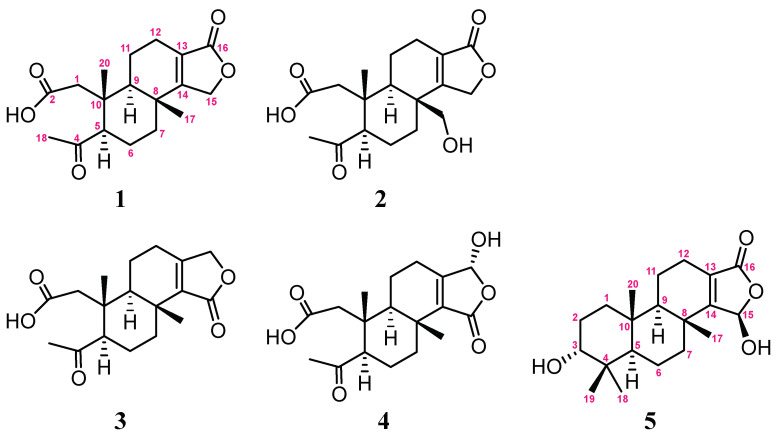
Structures of metabolites **1**–**5**.

**Figure 2:**
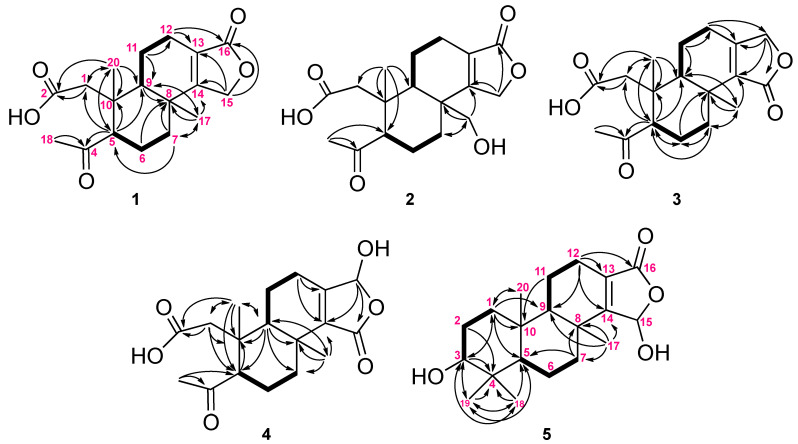
The selected COSY (▬) and HMBC (→) correlations of **1**–**5**.

**Figure 3:**
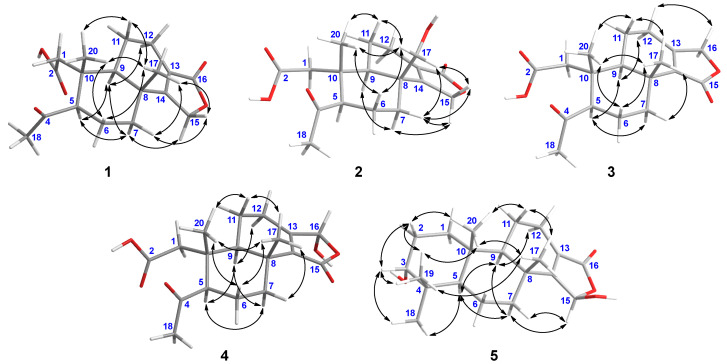
The key NOESY (↔) correlations of **1**–**5**.

**Figure 4:**
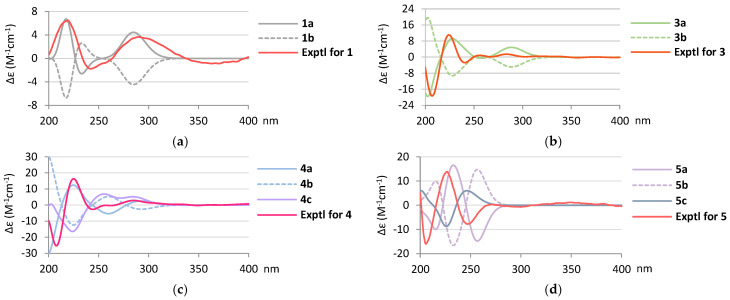
(**a**) Calculated ECD curves of 5*R*,8*R*,9*R*,10*R*-**1** (**1a**), 5*S*,8*S*,9*S*,10*S*-**1** (**1b**) and the experimental CD curve of **1**. (**b**) Calculated ECD curves of 5*R*,8*R*,9*R*,10*R*-**3** (**3a**), 5*S*,8*S*,9*S*,10*S*-**3** (**3b**) and the experimental CD curve of **3**. (**c**) Calculated ECD curves of 5*R*,8*R*,9*R*,10*R*,16*R*-**4** (**4a**), 5*S*,8*S*,9*S*,10*S*,16*S*-**4** (**4b**), 5*R*,8*R*,9*R*,10*R*,16*S*-**4** (**4c**) and the experimental CD curve of **4**. (**d**) Calculated ECD curves of 3*R*,5*R*,8*R*,9*R*,10*R*,15*R*-**5** (**5a**), 3*S*,5*S*,8*S*,9*S*,10*S*,15*S*-**5** (**5b**), 3*R*,5*R*,8*R*,9*R*,10*R*,15*S*-**5** (**5c**) and the experimental CD curve of **5**.

Spongian diterpenoids and structurally related metabolites are a series of natural products with unique structures distributed in sponges of the genus *Spongia*, and the structural diversity and potential biological significance of these compounds are well known [1,2,3,4,5,6,7,8,9,10,11]. Recently, we have reported the discovery of new spongian diterpenes, including four rare 5,5,6,6,5-pentacyclic diterpenoids, from a Red Sea sponge, *Spongia* sp. [12,13]. Some of these compounds have been shown to display antibacterial and anti-inflammatory activities. In our continuing study on the chemical constituents from this sponge, we further discovered four new 3,4-*seco*-3,19-dinorspongian diterpenoid lactones, secodinorspongins A−D (**1**−**4**), and one classical spongian diterpenoid lactone, sponginolide (**5**) (Figure 1). Compounds with the same carbon skeleton of **1**−**4** have been found only two times so far [1,2], and were discovered in Red Sea sponges for the first time. The relative structures and absolute configurations of these compounds were established by the analyses of MS, UV, IR, NMR, and CD spectra (Supplementary Figures S1–S51 and Figure 1, Figure 2, Figure 3 and Figure 4). Furthermore, the cytotoxicity, antibacterial and anti-inflammatory activities of **1**−**5** were assayed. Herein, we report the isolation, structural elucidation, and bioactivity evaluation of these compounds.

## 2. Results and Discussion

The lyophilized sponge *Spongia* sp. (550 g) was extracted with a solvent mixture of CH_2_Cl_2_/EtOAc/MeOH (1:1:0.5, *v/v*). The crude extract was partitioned between CH_2_Cl_2_ and H_2_O, to afford the CH_2_Cl_2_ fraction (18.5 g), which was fractionated repeatedly, using column chromatography, to yield compounds **1** (1.4 mg), **2** (4.3 mg), **3** (4.0 mg), **4** (1.3 mg) and **5** (3.0 mg) (Figure 1).

The HRESIMS of metabolite **1** exhibited the [M + H]^+^ peak at *m*/*z* 321.1696 (calcd for C_18_H_25_O_5_, 321.1697, Supplementary Figure S1) and established a molecular formula of C_18_H_24_O_5_, appropriate with seven degrees of unsaturation. The IR spectrum of **1** showed the presence of hydroxy (3462 cm^−1^), carbonyl (1748, 1698 and 1684 cm^−1^) and olefinic (1653 cm^−1^) functional groups (Supplementary Figure S3). The ^13^C NMR data of **1** displayed 18 carbon signals (Table 1), which were assigned with the assistance of the ^1^H and HSQC spectrum into three methyls (δ_C_ 31.8, 21.4, and 19.5), six methylenes (δ_C_ 44.2, 36.1, 22.2, 22.1, and 18.6, including one oxygenated methylene δ_C_ 68.8), two methines (δ_C_ 56.3 and 48.7) and seven non-protonated carbons (δ_C_ 211.6, 174.3, 172.8, 171.2, 123.9, 40.2, and 37.6). In total, the NMR spectroscopic data of **1** (Table 1) showed signals for an α,β-unsaturated *γ*-lactone (δ_C_ 174.3, C; 171.2, C; 123.9, C and 68.8, CH_2_; δ_H_ 4.86, dt, *J* = 17.0, 2.5 Hz, 1H and 4.72, dd, *J* = 17.0, 2.5 Hz, 1H) [2,12,14,15]. Furthermore, the ^1^H–^1^H COSY experiment showed the presence of two partial structures (Figure 2), which were further connected by analysis of the HMBC correlations (Figure 2), to establish the planar structure of **1** as 3,4-*seco*-3,19-dinorspongian diterpenoid lactone. 

In the NOESY spectrum of compound **1**, the following NOE interactions were found (Figure 3): H_3_-17 with H_3_-20, both H_3_-17 and H_3_-20 with one proton *(*δ_H_ 1.90) of H_2_-6 and one proton (δ_H_ 1.65) of H_2_-11, and H_3_-17 with one proton (δ_H_ 1.83) of H_2_-7 and one proton (δ_H_ 4.86) of H_2_-15. The above finding revealed that these protons must be located in the same orientation and were assumed to be β protons [2,12]. In contrast, the NOE correlations of the H-7α (δ_H_ 1.54) with both H-5 and H-9, and of H-9 with H-5 and H-12α (δ_H_ 2.05), indicated that these protons must be positioned on the α-face [2,12]. Furthermore, a comparison of the experimental ECD spectrum with those calculated for **1a** (5*R*,8*R*,9*R*,10*R*-**1**) and **1b** (5*S*,8*S*,9*S*,10*S*-**1**) allowed us to conclude the 5*R*,8*R*,9*R*,10*R*- configuration for **1** (Figure 4a). Accordingly, the structure of **1** was identified as a new 3,4-*seco*-3,19-dinorspongian diterpene, and named secodinorspongin A. 

The ^13^C and ^1^H NMR data of compound **2** are very similar to those of **1** (Table 1), except that a methyl group (δ_H_ 1.26, 3H, s and δ_C_ 21.4, CH_3_) at C-8 in **1** was oxidized to a hydroxymethyl (δ_H_ 4.15 and 3.71, each 1H, d, *J*= 10.0 Hz; δ_C_ 64.3, CH_2_) in **2**. The detailed analyses of 2D NMR correlations disclosed from the HMBC, COSY and NOESY experiments confirmed that **2** is the 17-hydroxylated derivative of **1** (Figure 2 and Figure 3). 

The NMR data of compound **3** are also similar to those of **1** (Table 1); however, notable differences were observed for NMR chemical shifts of CH_2_-7, CH_2_-12, and CH_3_-17. A comparison of their HMBC correlations also showed distinct differences in the correlation of H_2_-12. These protons correlated with the ester carbonyl carbon in **1** (Figure 2), whereas the same protons correlated with the oxygenated methylene carbon in **3** (Figure 2). Furthermore, the NOE correlations of H_3_-17 with H-7β and H_3_-20, and of H-5 with H-7α and H-9 suggested a 5R*,8R*,9R*,10R* relative configuration for **3** (Figure 3). 

A high similarity in the ^13^C and ^1^H NMR data between metabolites **3** and **4** (Table 1) was observed, with the exception being that the 16-oxymethylene group (δ_H_ 4.67 and 4.61, each 1H, d, *J* = 17.5 Hz; δ_C_ 71.3, CH_2_) in γ-lactone of **3** was converted to an acetal (δ_H_ 5.88, 1H, d, *J* = 4.0 Hz; δ_C_ 97.5, CH) in **4**. The detailed analyses observed by 2D NMR experiments confirmed that **4** is a 16-hydroxylated derivative of **3** (Figure 2). However, due to the lack of NOE interaction of H-16 with other protons in **4** (Figure 3), the configuration of C-16 was determined by comparison of the calculated ECD with experimental CD spectra (Figure 4c). The experimental CD curve of **4** was found to be quite similar to **4a** (5*R*,8*R*,9*R*,10*R* and 16*R*) rather than **4b** (5*S*,8*S*,9*S*,10*S* and 16*S*) or **4c** (5*R*,8*R*,9*R*,10*R* and 16*S*) (Figure 4c). On the basis of the above results, the structure of **4** was determined, and named secodinorspongin D.

**Scheme 1:**
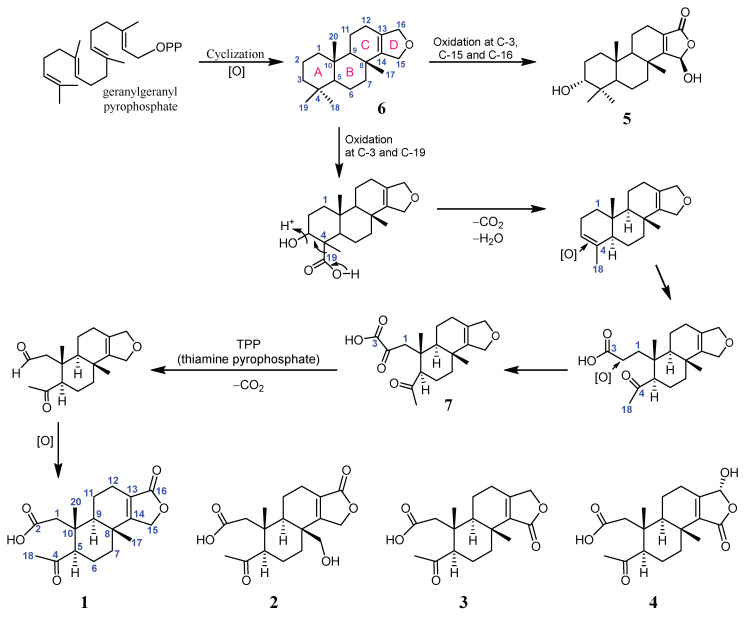
A plausible biosynthetic pathway for the formation of structures **1**–**5**.

It is suggested that compounds **1**–**4** share the same biogenetic origin, as they are obtained from the same organism. Consequently, we propose that **1**–**4** might be biosynthesized from the spongian diterpene (i.e., **6**, Scheme 1). The initial oxidation at C-3 and C-19 and the subsequent decarboxylation resulting in the loss of C-19 and dehydration, generate a methyl cyclohexene moiety. Further oxidative cleavage on the double bond of the cyclohexene ring and oxidation at C-2 give intermediate **7** (Scheme 1). With the loss of CO_2_ from the β-keto acid moiety and the subsequent oxidation at the relevant position, diterpenes **1**–**4** may be generated, as illustrated in Scheme 1. 

**Figure 5:**
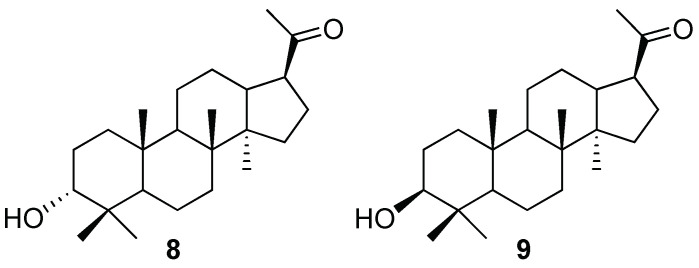
The structures of **8** and **9**.

The molecular formula of metabolite **5** was determined to be C_20_H_30_O_4_, from the HRESIMS (m/z 357.2035 [M + Na]^+^, calcd for C_20_H_30_O_4_Na, 357.2036), indicating six degrees of unsaturation. The IR spectrum displayed the absorptions of hydroxyl (3446 cm^−1^) and carbonyl (1748 cm^−1^) functionalities. The analysis of NMR spectral data, including ^1^H–^1^H COSY, HMBC, and NOE correlations (Table 2 and Figure 2 and Figure 3), suggested the gross structure of **5** was a (3R*, 5R*, 8R*, 9R*, 10R*)-spongian diterpene (Figure 3). Furthermore, the relative stereochemistry of C-3 was also identified by the comparison of the NMR data of **5** with those of the 3-epimeric analogs, aglaiabbreviatin C (**8**) [16] and 3*β*-hydroxy-22,23,24,25,26,27-hexanordammaran-20-one (**9**) [17] (Figure 5). The proton chemical shift and coupling patterns of H-3 (δ_H_ 3.44, t, *J* = 3.0 Hz) of **5** were similar to that of **8** (δ_H_ 3.40, t, *J* = 2.9 Hz) rather than that of **9** (δ_H_ 3.22, dd, *J* = 11.5 and 5.3 Hz) in CDCl_3_. Moreover, the experimental CD curve of **5** was found to be quite similar to **5a** (3*R*,5*R*,8*R*,9*R*,10*R*,15*R*-**5**) rather than **5b** (3*S*,5*S*,8*S*,9*S*,10*S*,15*S*-**5**) and **5c** (3*R*,5*R*,8*R*,9*R*, 10*R*,15*S*-**5**) (Figure 4d). On the basis of the above results, the structure of **5** was determined, and named sponginolide.

The cytotoxic, antibacterial and anti-inflammatory properties of **1**–**5** were analyzed in order to discover their potential medicinal application. The cytotoxicities of these compounds against the proliferation of the human hepatocellular carcinoma (HCC) Huh7 cell line were evaluated using the resazurin assay [18,19], and none of them showed notable inhibition regarding the growth of the HCC Huh7 cells. Furthermore, compound **1** exhibited 43%, 54%, and 75% inhibition at 50, 100, and 200 µM, respectively, in the assay for the growth inhibition of *S. aureus* (Table 3). In the anti-inflammatory assay (Table 4), compounds **1**–**5**, at 20 μM, exhibited suppression of superoxide anion generation (7.1 ± 5.3%, 7.2 ± 2.4%, 3.7 ± 2.7%, 20.4 ± 4.5 and 22.0 ± 4.6, respectively) and elastase release (12.2 ± 2.4%, 18.5 ± 1.7%, 17.6 ± 2.3%, 30.8 ± 2.6 and 22.5 ± 4.2, respectively) by fMLF/CB-stimulated human neutrophils [20,21,22].

## 3. Materials and Methods

### 3.1. General Experimental Procedures

The NMR experiments of all the compounds were recorded on a Varian Unity INOVA 500 FT-NMR (Varian Inc., Palo Alto, CA, USA). Specific optical rotations were performed in MeOH on the Jasco P-1020 polarimeter (JASCO Corporation, Tokyo, Japan). The IR spectra were recorded on an FT/IR-4100 infrared spectrophotometer (JASCO Corporation, Tokyo, Japan). Measurements of circular-dichroism spectra were performed on a Jasco J-715 CD spectrometer (JASCO Corporation, Tokyo, Japan). HRESIMS were measured on the Impact HD Q-TOF (Bruker, Bremen, Germany) mass spectrometer. Pre-coated silica gel plates (Silica gel 60 F254, 100 μm, Merck, Darmstadt, Germany), or C18 gel plates (Silica gel 60 RP-18 F_254_s, 100 μm, Merck, Darmstadt, Germany) were used for analytical thin-layer chromatography (TLC). The silica gel (40−63 μm, Merck, Billerica, U.S.A.) and reversed-phase silica gel (RP-18, 40−63 μm, Merck, Darmstadt, Germany) were applied for column chromatography. The Hitachi L-2455 HPLC apparatus (Hitachi, Tokyo, Japan) with a Supelco C18 column (250 × 21.2 mm, 5 μm, Supelco, Bellefonte, PA, USA) were used for HPLC.

### 3.2. Animal Material

The sponges *Spongia* sp. were collected from the coast of the Red Sea (21^o^22′11.08″ N, 39^o^06′56.62″ E), Saudi Arabia, in March 2016. The species of sponges was identified by Prof. Y. M. Huang. The voucher sample (RSS-1) was deposited at the Department of Pharmacognosy, College of Pharmacy, King Saud University, Saudi Arabia.

### 3.3. Extraction and Separation

The freeze-dried sponges (550 g) were minced and extracted with MeOH/EtOAc/ CH_2_Cl_2_ (1/1/0.5). The crude extract was suspended in water and partitioned with CH_2_Cl_2_ to obtain CH_2_Cl_2_ (18.47 g) fraction. The CH_2_Cl_2_ fraction was chromatographed on silica gel with *n*-hexane−EtOAc (100:0 to 0:100, stepwise) and then EtOAc−MeOH (3:1 to 0:100, stepwise) to yield 12 fractions (F1–F12).

Fraction F3 (0.987 g, EtOAc/*n*-hexane 1:9) was isolated using column chromatography on the reversed-phase silica gel with H_2_O−MeOH (100:0 to 0:100, stepwise), to yield six subfractions (F3-1 to F3-6). Subfraction F3-4 (79.1 mg, MeOH/H_2_O 3:2) was further isolated using reversed-phase HPLC (MeOH/H_2_O 7:3), to give seven subfractions (F3-4-1 to F3-4-7). F3-4-3 (18.3 mg) was purified using reversed-phase HPLC (MeOH/H_2_O 13:12), to obtain compounds **1** (1.4 mg) and **3** (4.0 mg). 

Fraction F7 (1.505 g, EtOAc/*n*-hexane 3:1) was isolated using column chromatography on the reversed-phase silica gel with H_2_O−MeOH (100:0 to 0:100, stepwise), to yield eight subfractions (F7-1 to F7-8). Subfraction F7-3 (146.3 mg, MeOH/H_2_O 2:3) was further isolated using reversed-phase HPLC (MeOH/H_2_O 1:1), to give ten subfractions (F7-3-1 to F7-3-10). F7-3-6 (28.2 mg) was purified using reversed-phase HPLC (CH_3_CN /H_2_O 1:3), to obtain **2** (4.3 mg) and **4** (1.3 mg). F7-5 (72.1 mg, MeOH/H_2_O 8:2) was isolated using reversed-phase HPLC (MeOH/H_2_O 9:1), to give 6 subfractions (F7-5-1 to F7-5-6); F7-5-3 (19.3 mg) was purified using reversed-phase HPLC (MeOH/H_2_O 1:1), to afford **5** (3.0 mg).

#### 3.3.1. Secodinorspongin A (**1**)

Colorless oil, ${\left[\alpha \right]}_{D}^{25}$ −34.3 (*c* = 0.38, CH_3_OH); UV (CH_3_OH) λ_max_ (log*ε*): 208 nm (3.46); IR (neat) ν_max_ 3462, 2921, 1748, 1698, 1684 and 1653 cm^–1^; CD experimental data and cartesian coordinates of conformer **1a**, see Figure 4a and Supplementary Tables S1 and S2, respectively; ^1^H NMR and ^13^C data, see Table 1; HRESIMS *m/z* 321.1696 [M + H]^+^ (calcd for C_18_H_25_O_5_, 321.1697).

#### 3.3.2. Secodinorspongin B (**2**)

Colorless oil, ${\left[\alpha \right]}_{D}^{25}$ −44.3 (*c* = 0.43, CH_3_OH); UV (CH_3_OH) λ_max_ (log*ε*): 211 nm (3.58); IR (neat) ν_max_ 3393, 2948, 2836, 1733, 1697, 1684 and 1654 cm^–1^; CD experimental data, see Supplementary Table S3; ^1^H NMR and ^13^C data, see Table 1; HRESIMS *m/z* 337.1644 [M + H]^+^ (calcd for C_18_H_25_O_6_, 337.1646).

#### 3.3.3. Secodinorspongin C (**3**)

Colorless oil, ${\left[\alpha \right]}_{D}^{25}$ −29.0 (*c* = 0.40, CH_3_OH); UV (CH_3_OH) λ_max_ (log*ε*): 205 nm (3.27); IR (neat) ν_max_ 3392, 2947, 2835, 1734, 1698, 1684 and 1653 cm^–1^; CD experimental data and cartesian coordinates of conformer **3a**, see Figure 4b and Supplementary Tables S4 and S5, respectively; ^1^H NMR and ^13^C data, see Table 1; HRESIMS *m/z* 321.1699 [M + H]^+^ (calcd for C_18_H_25_O_5_, 321.1697).

#### 3.3.4. Secodinorspongin D (**4**)

Colorless oil, ${\left[\alpha \right]}_{D}^{25}$ −26.0 (*c* = 0.35, CH_3_OH); UV (CH_3_OH) λ_max_ (log*ε*): 209 nm (3.71); IR (neat) ν_max_ 3446, 2949, 1748, 1700, 1684 and 1654 cm^–1^; CD experimental data and cartesian coordinates of conformers **4a** and **4c**, see Figure 4c and Supplementary Tables S6–S8, respectively; ^1^H NMR and ^13^C data, see Table 1; HRESIMS *m/z* 337.1648 [M + H]^+^ (calcd for C_18_H_25_O_6_, 337.1646).

#### 3.3.5. Sponginolide (**5**)

Colorless oil, ${\left[\alpha \right]}_{D}^{25}$ +30.5 (*c* = 0.30, CH_3_OH); UV (CH_3_OH) λ_max_ (log*ε*): 208 nm (3.66); IR (neat) ν_max_ 3446, 2923, 2854 and 1748 cm^–1^; CD experimental data and cartesian coordinates of conformer **5a** and **5c**, see Figure 4d and Supplementary Tables S10–S12, respectively; ^1^H NMR and ^13^C data, see Table 2 and Supplementary Table S9; ESIMS *m/z* 357 [M + Na] ^+^; HRESIMS *m/z* 357.2035 [M + Na]^+^ (calcd for C_20_H_30_O_4_Na, 357.2036).

### 3.4. DFT and TD-DFT Calculations

The geometry optimization of conformers was performed using DFT calculation at B3LYP/6-31G + (d) level of theory, and the resulting conformers were subsequently calculated using the time-dependent DFT (TD-DFT) approach at CAM-B3LYP/6-311 + G(d,p) (compound **1**) or at B3LYP/6-31 + G(d,p) (compounds **3**–**5**) level of theory [23]. The calculations were performed using Gaussian 09 program [24] with the integral-equation-formalism-polarizable-continuum (IEFPCM) solvent model in MeOH. The ECD curves were simulated using GaussSum 2.2.5, and then illustrated with Microsoft Excel.

### 3.5. Cytotoxicity Assay 

The human hepatocellular carcinoma (HCC) Huh7 cells were used in the resazurin assay (Cayman Chemical) to evaluate the cytotoxicity of compounds (Supplementary Table S13). The method was performed as described previously [18,19], and the detailed process of the cytotoxicity assay was the same as in our previous publications [12,25]. Sorafenib, the positive control, inhibited 52% of the growth of Huh7 cells at 12.5 µM, and the DMSO controls were assigned 100% of relative cell-viability.

### 3.6. Antibacterial Assay

The antibacterial assay was processed using the previously reported methods [26]. The culture and dilution of *S. aureus* were performed as previously described [12,25]. The bacteria aliquots were plated (100 μL/well of 96-well plate) with the tested compounds (cpd) at concentrations of 50 μM, 100 μM, and 200 μM. A total of 1% DMSO in LB solution (background control), 1% DMSO in the diluted bacteria solution (positive control), and 0.5 µg/mL tetracycline (known-drug control) were plated on the same plate. After incubation at 37 °C for 16 h, the plate was measured by the absorbance at 600 nm (A), and then the percentage of *S. aureus* growth was measured using the following equation: [(Acpd—Acpd_basal) − Abackground control]/[(Apositive control − Apositive control_basal) − Abackground control] × 100. 

### 3.7. Anti-Inflammatory Activity

The methods using dextran sedimentation, Ficoll-Hypaque gradient centrifugation, and hypotonic lysis to enrich the neutrophils, which were isolated from the blood of healthy adult volunteers and incubated in Ca^2 +^ -free HBSS buffer (pH 7.4, ice-cold), were described in a previous paper [22]. 

### 3.8. Inhibition of Superoxide Anion Generation

The method was performed as described previously [20,21], and the detailed process of incubating and treating neutrophils was the same as in our previous publications [12,25]. After cytochalasin B (CB, 1 μg/mL) for 3 min, the 100 nM fMLF for 10 min (fMLF/CB) was used to activate neutrophils, and the positive control LY294002 [2-(4-morpholinyl)-8-phenyl-1(4*H*)-benzopyran-4-one] was used. The wavelength 550 nm (spectrophotometer U-3010, Hitachi, Tokyo, Japan) was used to measure the generation of the superoxide anion.

### 3.9. Inhibition of Elastase Release

The method was performed as described previously [20,21], and the detailed process of incubating and treating neutrophils was the same as in our previous publications [12,25]. The fMLF (100 nM)/CB (0.5 μg/mL) for 10 min, was used to activate neutrophils, and the wavelength 405 nm (spectrophotometer U-3010, Hitachi, Tokyo, Japan) was used to measure the generation of elastase release.

## 4. Conclusions

The new dinorditerpenoid lactones **1**−**4** have the rare 3,4-*seco*-3,19-dinorspongian structure. In the previous study, compounds of this skeleton [1,2] did not show cytotoxicity, and the same situation was also found for metabolites **1**−**4**. However, the current study revealed that compound **1** possessed notable inhibition against the growth of *S. aureus*, while **4** and **5** exhibited in vitro anti-inflammatory potential through the inhibitory activity against the generation of the superoxide anion and elastase release in fMLF/CB-induced human neutrophils. In prior studies, most of the spongian diterpenoids were not found to exhibit conspicuous biological activities [1,2,4,6,12,13]; however, few analogs with 3,19-dihydroxyl-2-one fragment in the A-ring and/or furano D-ring [2,3,4,8,9,10,11,13,27] were reported to have potent cytotoxicity, anti-inflammatory, and anti-viral activities. In this study, compounds **1**−**5,** which lack the aforementioned functionality, could be responsible for the deficiency of cytotoxic and anti-inflammatory activities.

## Figures and Tables

**Table 1:** ^13^C and ^1^H NMR data for compounds **1**–**4** (125/500 MHz) in acetone-*d*_6_.

	1		2		3		4	
Position	δ_H_	δ_C_	δ_H_	δ_C_	δ_H_	δ_C_	δ_H_	δ_C_
1	2.30, d (14.5) ^a^	44.2, CH_2_	2.29, m	44.5, CH_2_	2.30, d (14.5)	44.4, CH_2_	2.29, d (14.5)	44.3, CH_2_
	2.57, d (14.5)		2.54, d (13.0)		2.55, d (14.5)		2.54, d (14.5)	
2	−	172.8, C	−	174.8, C	−	172.8, C	−	172.9, C
4	−	211.6, C	−	211.9 C	−	211.9, C	−	211.9, C
5	3.15, dd (13.0, 3.5)	56.3, CH	3.20, br d (11.5)	56.2, CH	3.11, dd (12.5, 3.5)	56.5, CH	3.11, dd (12.5, 3.5)	56.4, CH
6α	1.78, ddd (13.5, 7.0, 3.5)	22.1, CH_2_	1.73, m	21.9, CH_2_	1.75, ddd (13.5, 7.0, 3.5)	22.1, CH_2_	1.74, ddd (13.5, 7.0, 3.5)	22.1, CH_2_
6β	1.90, dd (13.5, 3.5)		1.87, m		1.82, td (13.5, 3.5)		1.81, dd (13.5, 3.5)	
7α	1.54, td (13.5, 3.5)	36.1, CH_2_	1.33, m	30.9, CH_2_	1.22, td (13.5, 3.5)	35.2, CH_2_	1.25, td (13.5, 3.5)	35.5, CH_2_
7β	1.83, dt (13.5, 3.5)		2.27, m		2.61, dt (13.5, 3.5)		2.57, dt (13.5, 3.5)	
8	−	37.6, C	−	43.7, C	−	35.9, C	−	35.9, C
9	1.67, m	48.7, CH	1.77, m	48.9, CH	1.63, m	49.6, CH	1.59, m	49.3, CH
10	−	40.2, C	−	40.1, C	−	40.2, C	−	40.2, C
11α	2.06, m	18.6, CH_2_	2.05, m	18.1, CH_2_	2.07, m	18.7, CH_2_	2.06, m	18.7, CH_2_
11β	1.65, m		1.77, m		1.63, m		1.59, m	
12α	2.05, m	22.2, CH_2_	2.11, m	22.1, CH_2_	2.34, m	25.6, CH_2_	2.37, m	24.7, CH_2_
12β	2.29, m		2.29, m		2.48, m		2.53, m	
13	−	123.9, C	−	125.1, C	−	161.2, C	−	160.1, C
14	−	171.2, C	−	169.8, C	−	135.1, C	−	138.2, C
15α	4.72, dd (17.0, 2.5)	68.8, CH_2_	4.71, dd (17.0)	72.3, CH_2_	−	172.5, C	−	169.7, C
15β	4.86, dt (17.0, 2.5)		5.00, dt (17.0)					
16α	−	174.3, C	−	174.8, C	4.67, d (17.5)	71.3, C	−	97.5, CH
16β					4.61, d (17.5)		5.88, d (4.0)	
17	1.26, s	21.4, CH_3_	3.71, d (10.0)	64.3, CH_2_	1.18, s	20.8, CH_3_	1.17, s	21.4, CH_3_
			4.15, d (10.0)					
18	2.23, s	31.8, CH_3_	2.23, s	31.9, CH_3_	2.22, s	31.7, CH_3_	2.22, s	31.7, CH_3_
20	1.07, s	19.5, CH_3_	1.01, s	20.0, CH_3_	1.05, s	19.5, CH_3_	1.04, s	19.5, CH_3_

^a^*J* values (Hz) in parentheses.

**Table 2:** ^13^C and ^1^H NMR data for compound **5** (150/600 MHz) in CDCl_3_.

5					
Position	δ_H_	δ_C_	Position	δ_H_	δ_C_
1	1.35, m	32.9, CH_2_	10	−	37.5, C
	1.51, m		11	1.52, m	16.8, CH_2_
2α	1.61, m	24.9, CH_2_	12α	2.13, m	21.4, CH_2_
2β	2.00, t (3.0) ^a^		12β	2.41, dd (18.6, 6.6)	
3	3.44, t (3.0)	75.9, CH	13	−	127.7, C
4	−	37.6, C	14	−	167.6, C
5	1.41, m	49.2, CH	15	6.06, br s	96.8, CH
6	1.57, m	17.7, CH_2_	16	−	173.5, C
7α	1.50, m	36.8, CH_2_	17	1.25, s	20.6, CH_3_
7β	2.04, m		18	0.96, s	28.2, CH_3_
8	−	37.6, C	19	0.87, s	21.8, CH_3_
9	1.18, m	55.6, CH	20	0.93, s	16.4, CH_3_

^a^ *J* values (Hz) in parentheses.

**Table 3:** Inhibitory effects of compounds **1**–**5** on the growth of *S. aureus*.

Compound	Growth of *S. aureus*		
	50 µM (%, Mean ± SD)	100 µM (%, Mean ± SD)	200 µM (%, Mean ± SD)
**1**	57.2 ± 6.4	46.4 ± 22.3	25.0 ± 20.5
**2**	96.7 ± 3.1	96.3 ± 4.7	96.2 ± 4.8
**3**	101.2 ± 2.4	100.4 ± 2.3	100.1 ± 3.5
**4**	100.4 ± 9.9	98.4 ± 2.3	95.7 ± 7.8
**5**	102.2 ± 6.9	103.2 ± 8.1	101.5 ± 7.7
Tetracycline ^a^	0.9 ± 0.3		

^a^ Tetracycline was used as a positive control at 0.5 µg/mL.

**Table 4:** Inhibitory effects of compounds **1**–**5** on superoxide anion generation and elastase release, zº by human neutrophils.

Compound	Superoxide Anion		Elastase Release	
	IC_50_ (μM) ^a^	Inh% ^b^	IC_50_ (μM)	Inh%
**1**	>20	7.1 ± 5.3	>20	12.2 ± 2.4 **
**2**	>20	7.2 ± 2.4 *	>20	18.5 ± 1.7 ***
**3**	>20	3.7 ± 2.7	>20	17.6 ± 2.3 **
**4**	>20	20.4 ± 4.5 **	>20	30.8 ± 2.6 ***
**5**	>20	22.0 ± 4.6 **	>20	22.5 ± 4.2 **
LY294002	1.9 ± 0.8 ***	88.7 ± 1.5 ***	2.9 ± 0.1 ***	79.5 ± 2.0 ***

Results are presented as mean ± S.E.M. (n = 3). * *p* < 0.05, ** *p* < 0.01, *** *p* < 0.001 compared with the control value (DMSO). ^a^ Concentration required for 50% inhibition (IC50). ^b^ Percentage of inhibition (Inh %) at 20 μM.

## Data Availability

Data from the present study are available in the article and Supplementary Materials.

## Supplementary Materials

The supporting information can be downloaded at https://www.mdpi.com/article/10.3390/ijms24021252/s1.

## References

[1] Liang Y., Liao X., Ling L., Yang Y., Zhao B., Xu S. (2022). A new dinorspongian diterpene with pyridyl D-ring from the marine sponge *Spongia* sp.. Chin. J. Org. Chem..

[2] Liang Y.-Q., Liao X.-J., Zhao B.-X., Xu S.-H. (2020). Novel 3,4-*seco*-3,19-dinorspongian and 5,17-epoxy-19-norspongian diterpenes from the marine sponge *Spongia* sp.. Org. Chem. Front..

[3] Jomori T., Setiawan A., Sasaoka M., Arai M. (2019). Cytotoxicity of new diterpene alkaloids, ceylonamides G−I, isolated from Indonesian marine sponge of *Spongia* sp.. Nat. Prod. Commun..

[4] Chen Q., Mao Q., Bao M., Mou Y., Fang C., Zhao M., Jiang W., Yu X., Wang C., Dai L. (2019). Spongian diterpenes including one with a rearranged skeleton from the marine sponge *Spongia officinalis*. J. Nat. Prod..

[5] Liang Y.-Q., Liao X.-J., Lin J.-L., Xu W., Chen G.-D., Zhao B.-X., Xu S.-H. (2019). Spongiains A−C: Three new spongian diterpenes with ring A rearrangement from the marine sponge *Spongia* sp.. Tetrahedron.

[6] Maximo P., Ferreira L.M., Branco P., Lima P., Lourenco A. (2016). The role of *Spongia* sp. in the discovery of marine lead compounds. Mar. Drugs.

[7] Ponomarenko L.P., Terent’eva N.A., Krasokhin V.B., Kalinovsky A.I., Rasskazov V.A. (2011). Terpenoid metabolites from *Spongia* spp. and their effects on nucleic acid biosynthesis in sea urchin eggs. Nat. Prod. Commun..

[8] Carroll A.R., Lamb J., Moni R., Hooper J.N.A., Quinn R.J. (2008). Spongian diterpenes with thyrotropin releasing hormone receptor 2 binding affinity from *Spongia* sp.. J. Nat. Prod..

[9] Mori D., Kimura Y., Kitamura S., Sakagami Y., Yoshioka Y., Shintani T., Okamoto T., Ojika M. (2007). Spongolactams, farnesyl transferase inhibitors from a marine sponge: Isolation through an LC/MS-Guided assay, structures, and semisyntheses. J. Org. Chem..

[10] Gunasekera S.P., Schmitz F.J. (1991). New spongian diterpenoids from a Great Barrier Reef sponge, *Spongia* sp.. J. Org. Chem..

[11] Kohmoto S., Mcconnell O.J., Wright A., Cross S. (1987). Isospongiadiol, a cytotoxic and antiviral diterpene from a Caribbean deep-water marine sponge, *Spongia* sp.. Chem. Lett..

[12] Tai C.-J., Ahmed A.F., Chao C.-H., Yen C.-H., Hwang T.-L., Chang F.-R., Huang Y.M., Sheu J.-H. (2022). Spongenolactones A−C, bioactive 5,5,6,6,5-pentacyclic spongian diterpenes from the Red Sea sponge *Spongia* sp.. Mar. Drugs.

[13] Tai C.-J., Huang C.-Y., Ahmed A.-F., Orfali R.-S., Alarif W.-M., Huang Y.M., Wang Y.-H., Hwang T.-L., Sheu J.-H. (2021). An anti-inflammatory 2,4-cyclized-3,4-secospongian diterpenoid and furanoterpene-related metabolites of a marine sponge *Spongia* sp. from the Red Sea. Mar. Drugs.

[14] Zeng L.-M., Guan Z., Su J.-Y., Feng X.-L., Cai J.-W. (2001). Two new spongian diterpene lactones. Acta Chim. Sin..

[15] Li C.-J., Schmitz F.J., Kelly-Borges M. (1999). Six new spongian diterpenes from the sponge *Spongia matamata*. J. Nat. Prod..

[16] Zhang F., Wang J.-S., Gu Y.-C., Kong L.-Y. (2010). Triterpenoids from *Aglaia abbreviata* and their cytotoxic activities. J. Nat. Prod..

[17] Tanaka R., Matsuda M., Matsunaga S. (1987). 3β-Hydroxyhexanordammaran-20-one from *Euphorbia supina*. Phytochemistry.

[18] Chen Y.-S., Chang H.-S., Hsiao H.-H., Chen Y.-F., Kuo Y.-P., Yen F.-L., Yen C.-H. (2021). Identification of *beilschmiedia tsangii* root extract as a liver cancer cell-normal keratinocyte dual-selective NRF2 regulator. Antioxidants.

[19] Kao Y.-T., Chen Y.-S., Tang K.-W., Lee J.-C., Tseng C.-H., Tzeng C.-C., Yen C.-H., Chen Y.-L. (2020). Discovery of 4-anilinoquinolinylchalcone derivatives as potential NRF2 activators. Molecules.

[20] Yu H.-P., Hsieh P.-W., Chang Y.-J., Chung P.-J., Kuo L.-M., Hwang T.-L. (2011). 2-(2-Fluorobenzamido)benzoate ethyl ester (EFB-1) inhibits superoxide production by human neutrophils and attenuates hemorrhagic shock-induced organ dysfunction in rats. Free Radic. Biol. Med..

[21] Yang S.-C., Chung P.-J., Ho C.-M., Kuo C.-Y., Hung M.-F., Huang Y.-T., Chang W.-Y., Chang Y.-W., Chan K.-H., Hwang T.-L. (2013). Propofol inhibits superoxide production, elastase release, and chemotaxis in formyl peptide-activated human neutrophils by blocking formyl peptide receptor 1. J. Immunol..

[22] Hwang T.-L., Su Y.-C., Chang H.-L., Leu Y.-L., Chung P.-J., Kuo L.-M., Chang Y.-J. (2009). Suppression of superoxide anion and elastase release by C18 unsaturated fatty acids in human neutrophils. J. Lipid Res..

[23] Pescitelli G., Bruhn T. (2016). Good computational practice in the assignment of absolute configurations by TDDFT calculations of ECD spectra. Chirality.

[24] Frisch M.J., Trucks G.W., Schlegel H.B., Scuseria G.E., Robb M.A., Cheeseman J.R., Scalmani G., Barone V., Mennucci B., Petersson G.A. (2013). Gaussian 09.

[25] Tai C.-J., Ahmed A.F., Chao C.-H., Yen C.-H., Hwang T.-L., Chang F.-R., Huang Y.M., Sheu J.-H. (2022). The chemically highly diversified metabolites from the Red Sea marine sponge *Spongia* sp.. Mar. Drugs.

[26] Jiang L., Watkins D., Jin Y., Gong C., King A., Washington A.Z., Green K.D., Garneau-Tsodikova S., Oyelere A.K., Arya D.P. (2015). Rapid synthesis, RNA binding, and antibacterial screening of a peptidic-aminosugar (PA) library. ACS Chem. Biol..

[27] Betancur-Galvis L., Zuluaga C., Arno M., Gonzalez M.A., Zaragoza R.J. (2002). Cytotoxic effect (on tumor cells) and in vitro antiviral activity against herpes simplex virus of synthetic spongiane diterpenes. J. Nat. Prod..

