# Supplementary material for: New 3,4-seco-3,19-Dinor- and Spongian-Based Diterpenoid Lactones from the Marine Sponge Spongia sp
Source: Int J Mol Sci. 2023 Jan 8;24(2):1252. doi: 10.3390/ijms24021252 (PMC9860656; doi:10.3390/ijms24021252)
Supplement: Supplementary file 1 [file ijms-24-01252-s001.zip › ijms-2133757-supplementary.pdf]

New 3,4-*seco*-3,19-Dinor- and Spongian-Based Diterpenoid Lactones from the Marine Sponge *Spongia* sp.

Chi-Jen Tai [1,2], Chih-Hua Chao [3,4], Atallah F. Ahmed [5,6], Chia-Hung Yen [7,8], Tsong-Long Hwang [9,10,11], Fang-Rong Chang [7], Yusheng M. Huang [12,13], and Jyh-Horng Sheu [1,7,14,*]

[1] Department of Marine Biotechnology and Resources, National Sun Yat-sen University, Kaohsiung 80424, Taiwan
[2] National Museum of Marine Biology and Aquarium, Pingtung 944401, Taiwan
[3] School of Pharmacy, China Medical University, Taichung 40604, Taiwan
[4] Chinese Medicine Research and Development Center, China Medical University Hospital, Taichung 40604, Taiwan
[5] Department of Pharmacognosy, College of Pharmacy, King Saud University, Riyadh 11451, Saudi Arabia
[6] Department of Pharmacognosy, Faculty of Pharmacy, Mansoura University, Mansoura 35516, Egypt
[7] Graduate Institute of Natural Products, College of Pharmacy, Kaohsiung Medical University, Kaohsiung 80708, Taiwan
[8] National Natural Product Libraries and High-Throughput Screening Core Facility, Kaohsiung Medical University, Kaohsiung 80708, Taiwan
[9] Graduate Institute of Natural Products, College of Medicine, Chang Gung University, Taoyuan 333, Taiwan
[10] Research Center for Chinese Herbal Medicine, Graduate Institute of Healthy Industry Technology, College of Human Ecology, Chang Gung University of Science and Technology, Taoyuan 33303, Taiwan
[11] Department of Anesthesiology, Chang Gung Memorial Hospital, Taoyuan 333423, Taiwan
[12] Department of Marine Recreation, National Penghu University of Science and Technology, Magong, Penghu 88046, Taiwan
[13] Tropical Island Sustainable Development Research Center, National Penghu University of Science and Technology, Magong, Penghu 88046, Taiwan
[14] Department of Medical Research, China Medical University Hospital, China Medical University, Taichung 404333, Taiwan
* Correspondence: sheu@mail.nsysu.edu.tw; Tel.: +886-7-525-2000 (ext. 5030); Fax: +886-7-525-5020

Contents

Table S1. The CD experimental data of **1**. 9

Table S2. The cartesian coordinates of conformer **1a**. 10

Table S3. The CD experimental data of **2**. 16

Table S4. The CD experimental data of **3**. 22

Table S5. The cartesian coordinates of conformer **3a**. 24

Table S6. The CD experimental data of **4**. 30

Table S7. The cartesian coordinates of conformer **4a**. 32

Table S8. The cartesian coordinates of conformer **4c**. 33

Table S9. ^{13}C and ^{1}H NMR data for compound **5** (125/500 MHz) in acetone-d_6. 34

Table S10. The CD experimental data of **5**. 43

Table S11. The cartesian coordinates of conformer **5a**. 45

Table S12. The cartesian coordinates of conformer **5c**. 46

Table S13. Cytotoxicity (ED_{50} μg/mL) of compounds **1**−**5**. 47

Figure S1. HRESIMS spectrum of **1**. 4

Figure S2. UV spectrum of **1**. 4

Figure S3. IR spectrum of **1**. 5

Figure S4. ^{1}H NMR spectrum of **1** in acetone-d_6. 5

Figure S5. ^{13}C NMR spectrum of **1** in acetone-d_6. 6

Figure S6. HSQC spectrum of **1** in acetone-d_6. 6

Figure S7. ^{1}H−^{1}H COSY spectrum of **1** in acetone-d_6. 7

Figure S8. HMBC spectrum of **1** in acetone-d_6. 7

Figure S9. NOESY spectrum of **1** in acetone-d_6. 8

Figure S10. HRESIMS spectrum of **2**. 11

Figure S11. UV spectrum of **2**. 11

Figure S12. IR spectrum of **2**.. 12

Figure S13. ^{1}H NMR spectrum of **2** in acetone-d_6. 12

Figure S14. ^{13}C NMR spectrum of **2** in acetone-d_6. 13

Figure S15. HSQC spectrum of **2** in acetone-d_6. 13

Figure S16. ^{1}H–^{1}H COSY spectrum of **2** in acetone-d_6. 14

Figure S17. HMBC spectrum of **2** in acetone-d_6. 14

Figure S18. NOESY spectrum of **2** in acetone-d_6. 15

Figure S19. HRESIMS spectrum of **3**. 17

Figure S20. UV spectrum of **3**. 17

Figure S21. IR spectrum of **3**. 18

Figure S22. ^{1}H NMR spectrum of **3** in acetone-d_6. 18

Figure S23. ^{13}C NMR spectrum of **3** in acetone-d_6. 19

Figure S24. HSQC spectrum of **3** in acetone-d_6. 19

Figure S25. ^{1}H–^{1}H COSY spectrum of **3** in acetone-d_6. 20

Figure S26. HMBC spectrum of **3** in acetone-d_6. 20

Figure S27. NOESY spectrum of **3** in acetone-d_6. 21

Figure S28. HRESIMS spectrum of **4**. 25

Figure S29. UV spectrum of **4**. 25

Figure S30. IR spectrum of **4**. 26

Figure S31. ^{1}H NMR spectrum of **4** in acetone-d_6. 26

Figure S32. ^{13}C NMR spectrum of **4** in acetone-d_6. 27

Figure S33. HSQC spectrum of **4** in acetone-d_6. 27

Figure S34. $^1H-^1H$ COSY spectrum of **4** in acetone-d_6. 28

Figure S35. HMBC spectrum of **4** in acetone-d_6. 28

Figure S36. NOESY spectrum of **4** in acetone-d_6. 29

Figure S37. HRESIMS spectrum of **5**. 35

Figure S38. UV spectrum of **5**. 35

Figure S39. IR spectrum of **5**. 36

Figure S40. ^1H NMR spectrum of **5** in $CDCl_3$. 36

Figure S41. ^{13}C NMR spectrum of **5** in $CDCl_3$. 37

Figure S42. HSQC spectrum of **5** in $CDCl_3$. 37

Figure S43. HMBC spectrum of **5** in $CDCl_3$. 38

Figure S44. NOESY spectrum of **5** in $CDCl_3$. 38

Figure S45. ^1H NMR spectrum of **5** in acetone-d_6. 39

Figure S46. ^{13}C NMR spectrum of **5** in acetone-d_6. 39

Figure S47. DEPT spectrum of **5** in acetone-d_6. 40

Figure S48. HSQC spectrum of **5** in acetone-d_6. 40

Figure S49. $^1H-^1H$ COSY spectrum of **5** in acetone-d_6. 41

Figure S50. HMBC spectrum of **5** in acetone-d_6. 41

Figure S51. NOESY spectrum of **5** in acetone-d_6. 42

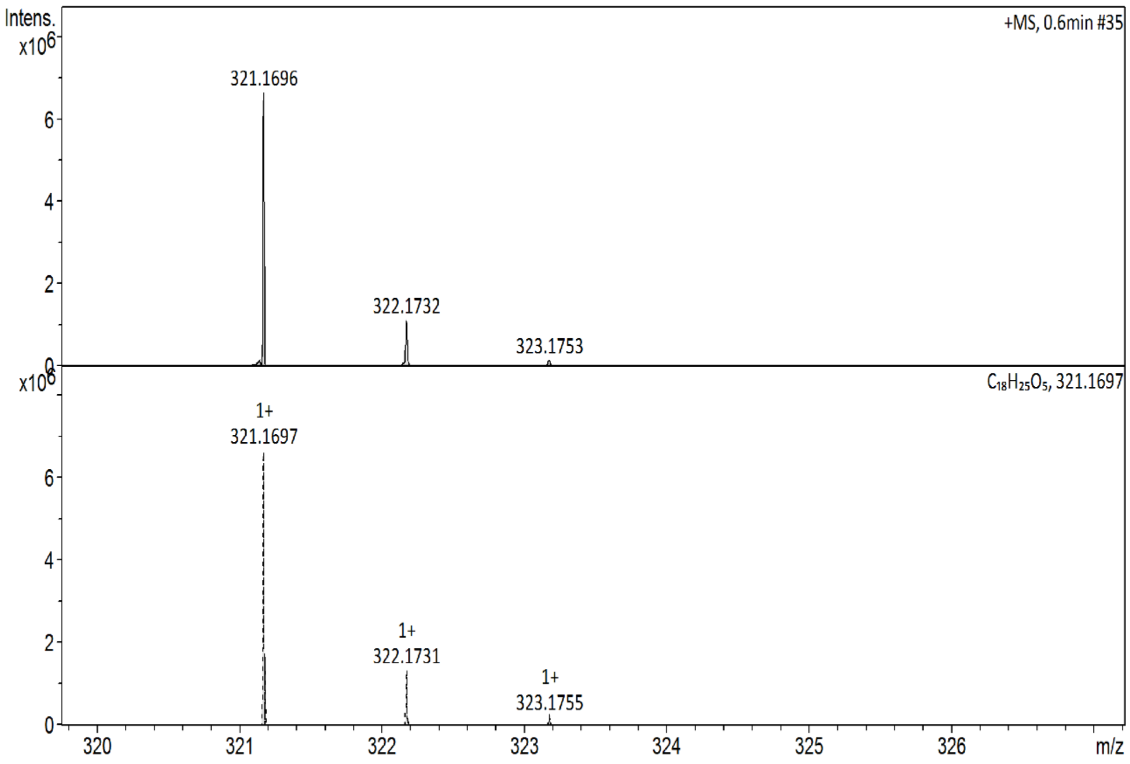

Figure S1. HRESIMS spectrum of **1**.

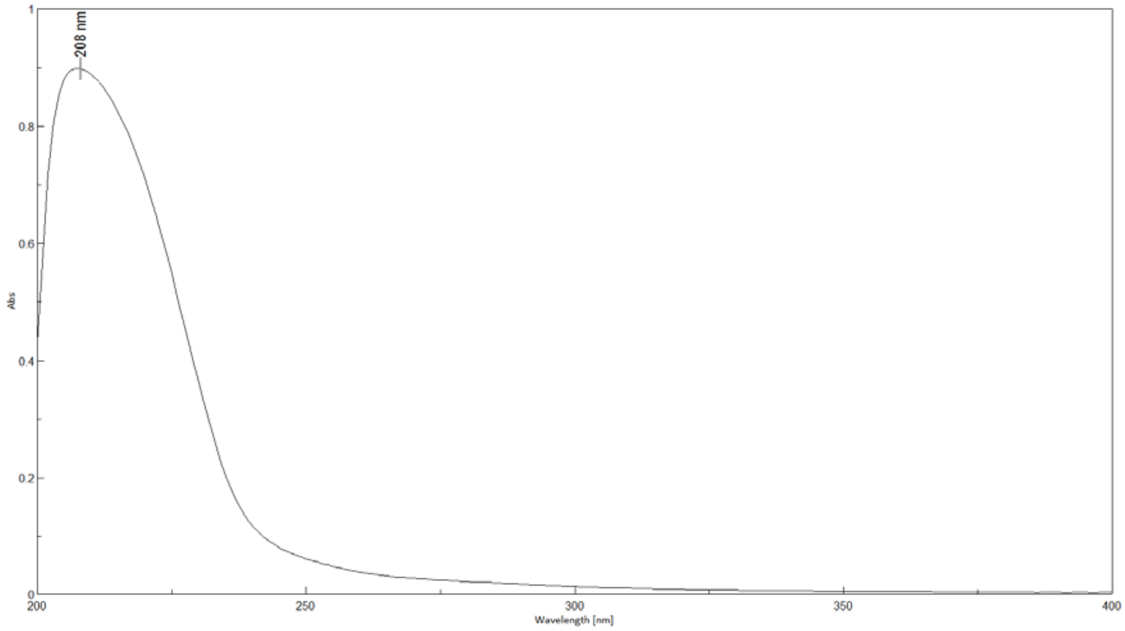

Figure S2. UV spectrum of **1**.

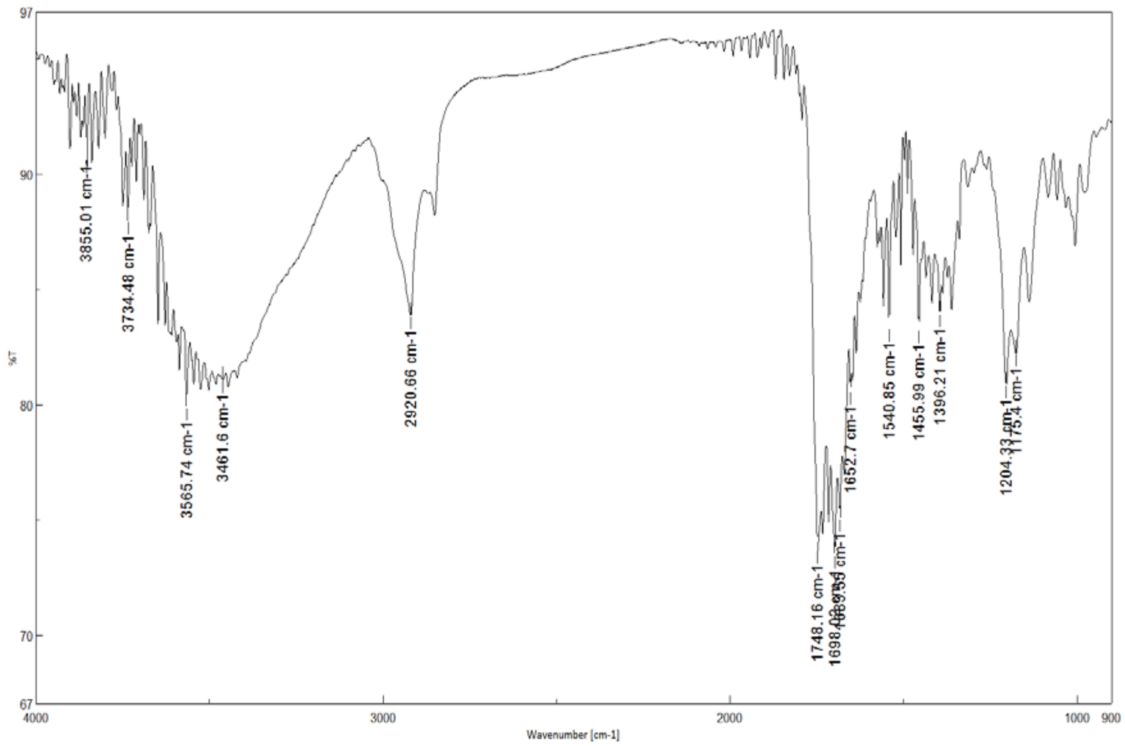

Figure S3. IR spectrum of **1**.

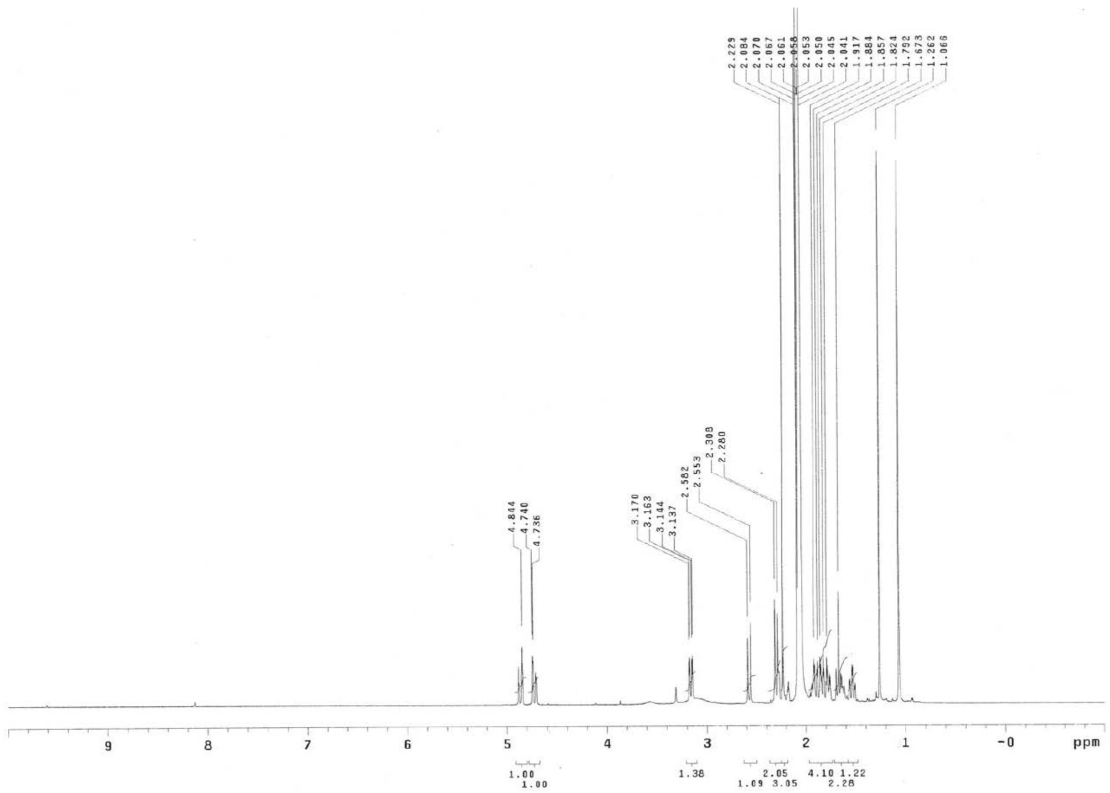

Figure S4. ^{1}H NMR spectrum of **1** in acetone-d_6.

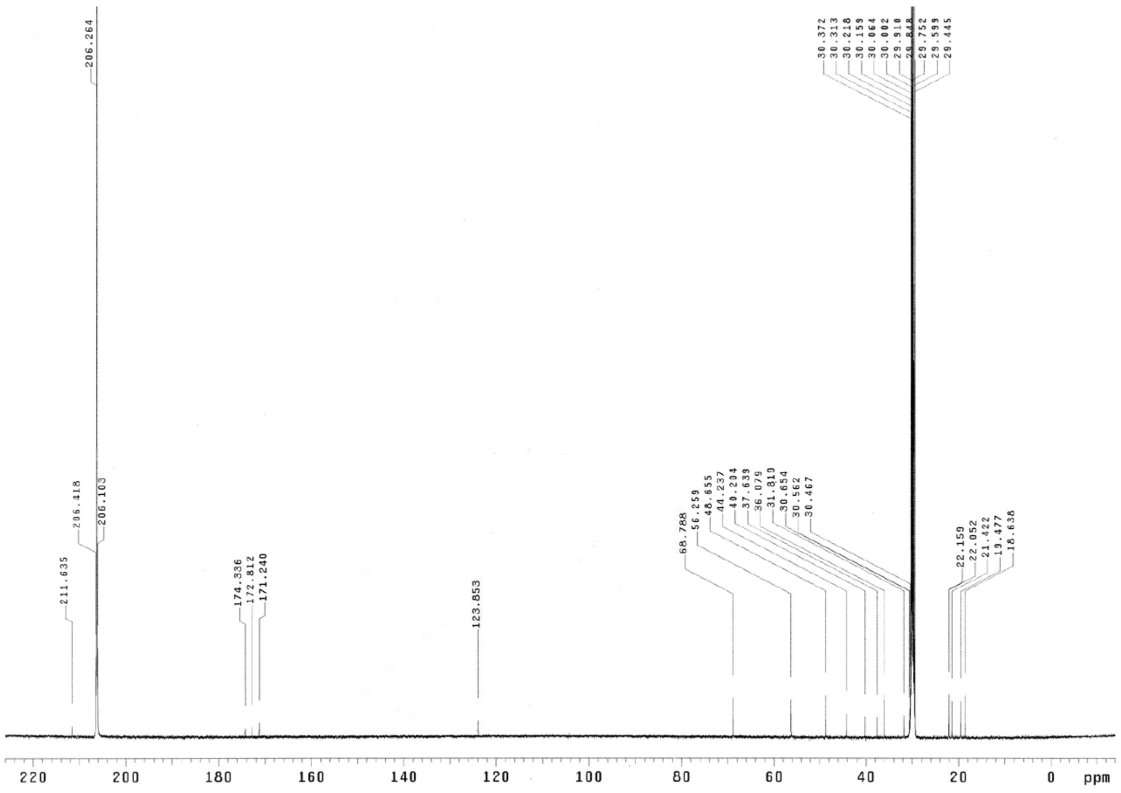

Figure S5.^{13}C NMR spectrum of **1** in acetone-d_6.

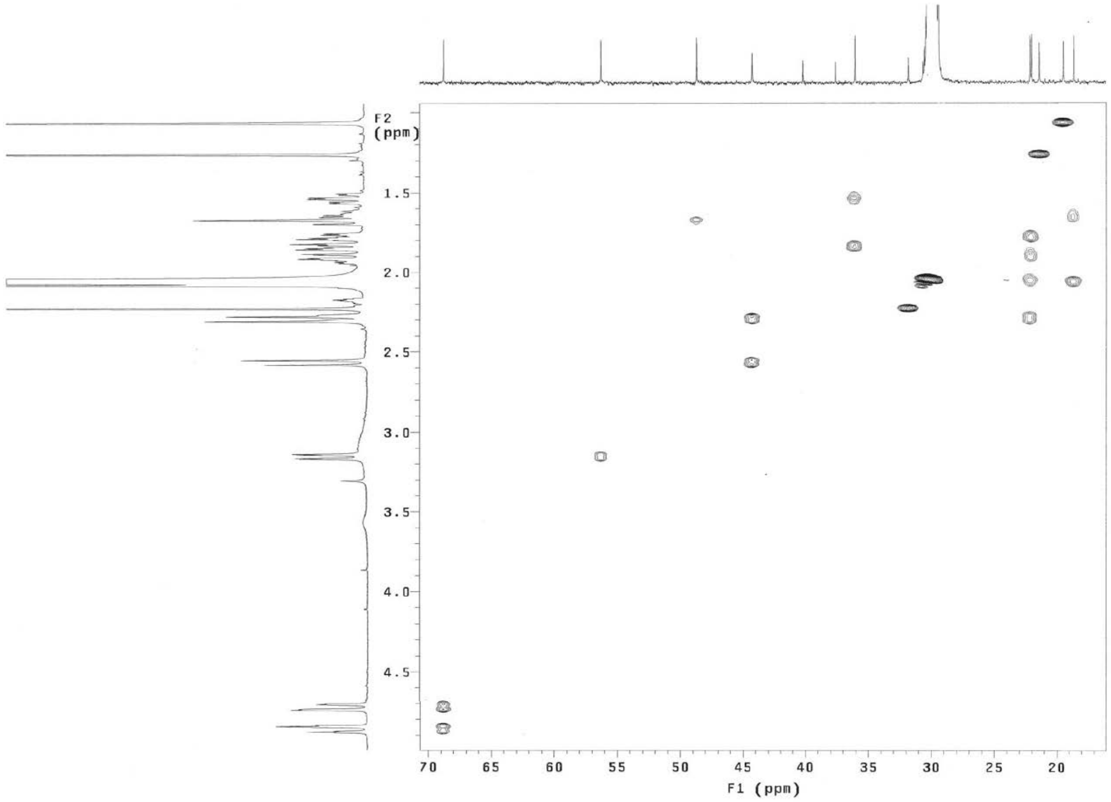

Figure S6. HSQC spectrum of **1** in acetone-d_6.

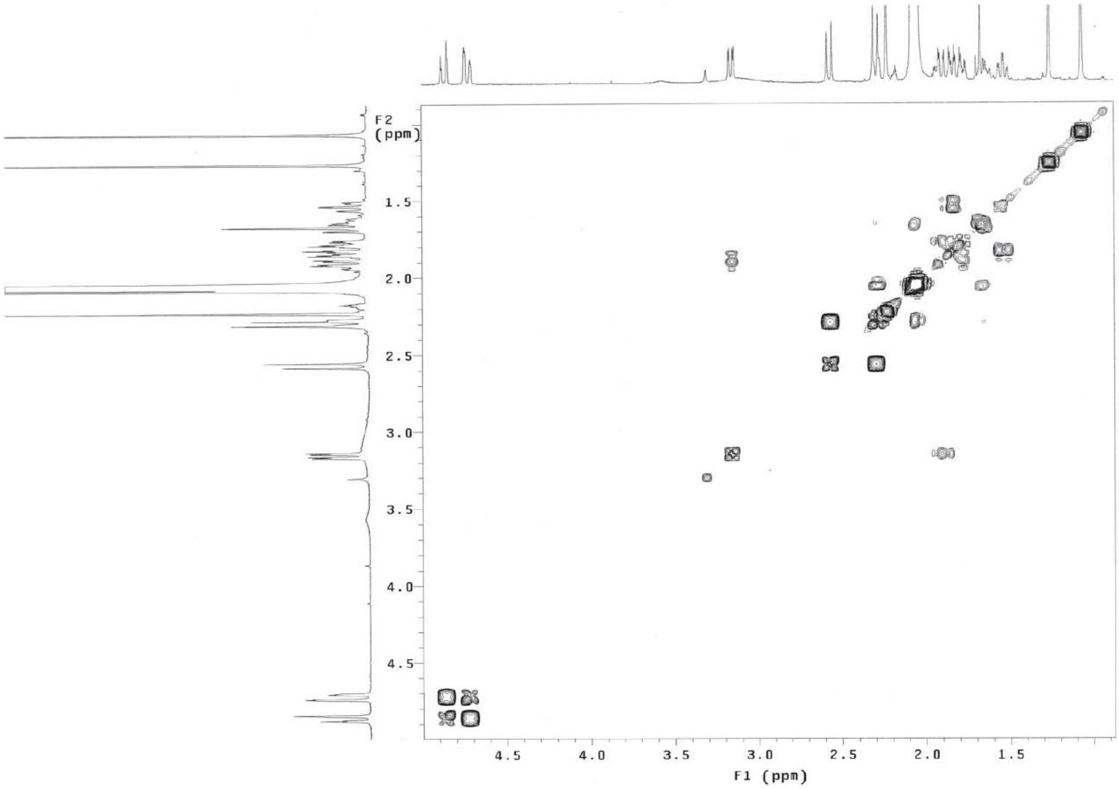

Figure S7. ^{1}H–^{1}H COSY spectrum of **1** in acetone-d_6.

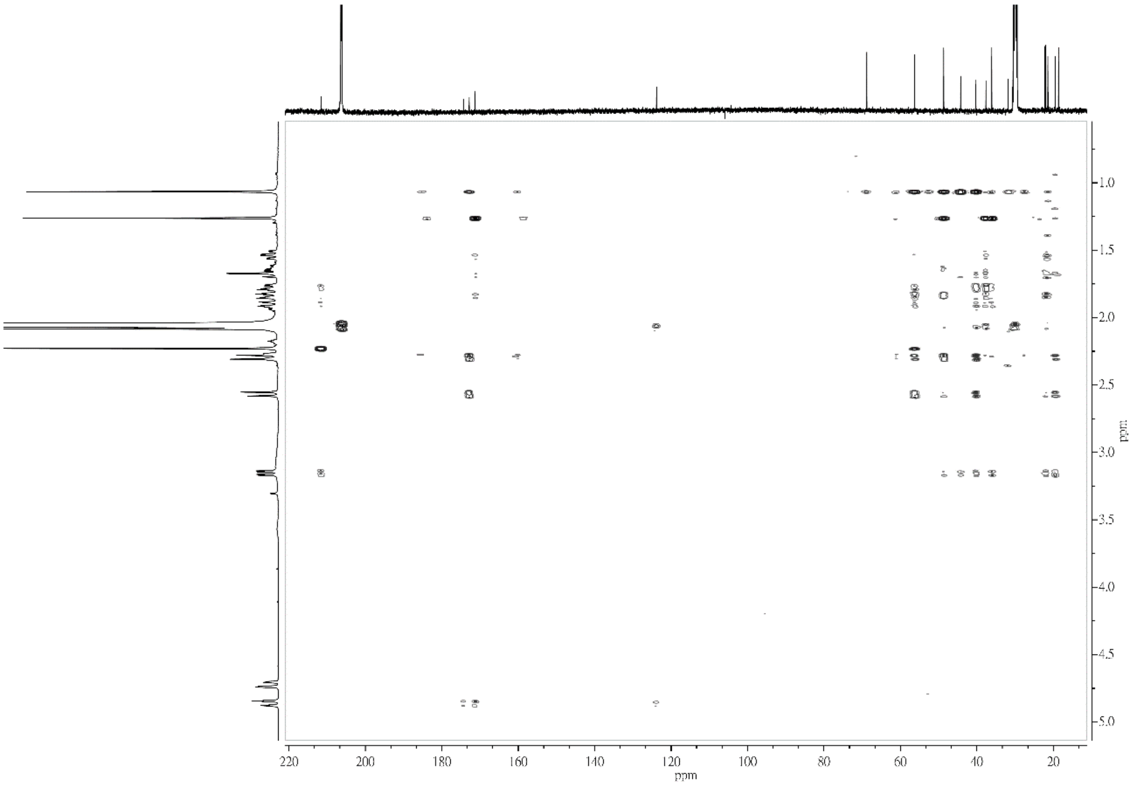

Figure S8. HMBC spectrum of **1** in acetone-d_6.

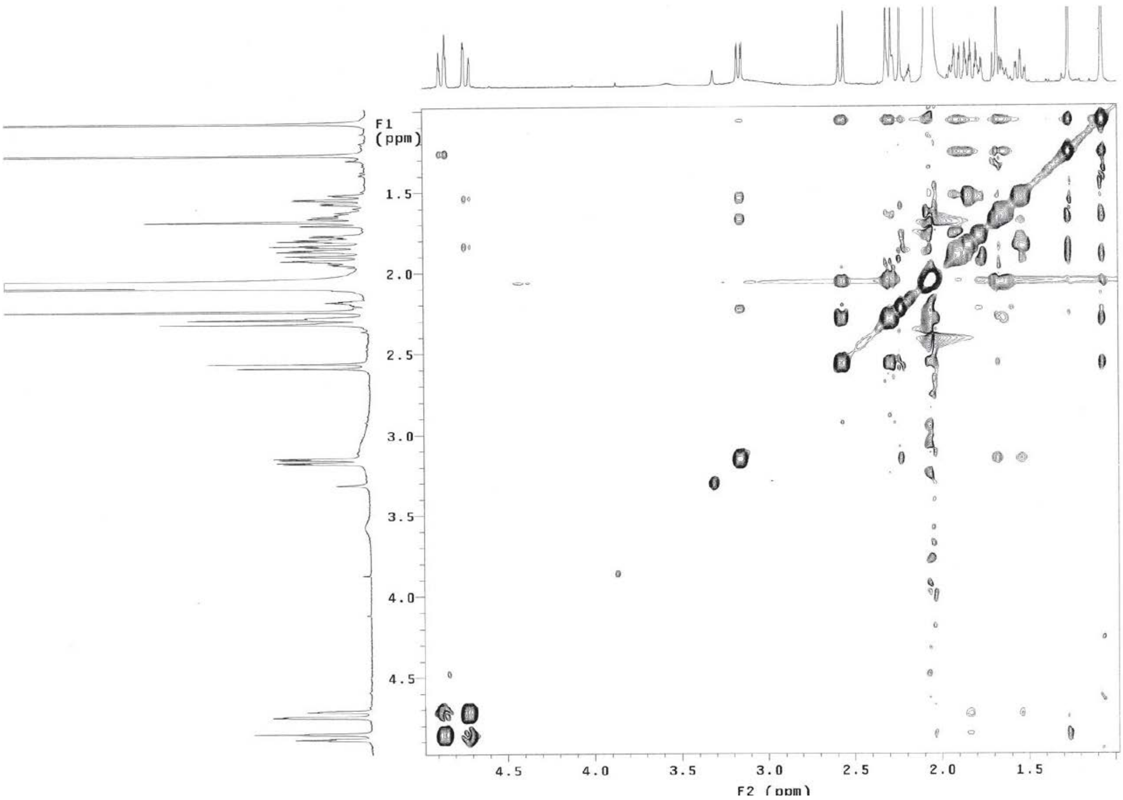

Figure S9. NOESY spectrum of **1** in acetone-d_6.

Table S1. The CD experimental data of **1**.

Wavelength [nm]	CD [mdeg]	Wavelength [nm]	CD [mdeg]	Wavelength [nm]	CD [mdeg]	Wavelength [nm]	CD [mdeg]
400.0	0.22852	349.0	-0.553456	298.0	3.42337	247.0	-1.47307
399.0	0.170548	348.0	-0.5284	297.0	3.4402	246.0	-1.55173
398.0	0.105764	347.0	-0.494044	296.0	3.45836	245.0	-1.62265
397.0	0.0452783	346.0	-0.466602	295.0	3.48476	244.0	-1.69152
396.0	-0.00779782	345.0	-0.443212	294.0	3.53628	243.0	-1.74783
395.0	-0.061939	344.0	-0.423078	293.0	3.57961	242.0	-1.76913
394.0	-0.121056	343.0	-0.3802	292.0	3.61251	241.0	-1.74318
393.0	-0.188866	342.0	-0.307237	291.0	3.64398	240.0	-1.64312
392.0	-0.270206	341.0	-0.210437	290.0	3.65969	239.0	-1.49438
391.0	-0.364143	340.0	-0.130332	289.0	3.65164	238.0	-1.30294
390.0	-0.451244	339.0	-0.0672454	288.0	3.60922	237.0	-1.09186
389.0	-0.52444	338.0	-0.0156641	287.0	3.54798	236.0	-0.802341
388.0	-0.56021	337.0	0.0406597	286.0	3.50198	235.0	-0.44379
387.0	-0.561007	336.0	0.108065	285.0	3.46359	234.0	-0.0243583
386.0	-0.535714	335.0	0.185083	284.0	3.40291	233.0	0.450151
385.0	-0.516273	334.0	0.268266	283.0	3.32185	232.0	0.965092
384.0	-0.516462	333.0	0.35648	282.0	3.19392	231.0	1.52869
383.0	-0.547374	332.0	0.459581	281.0	3.04967	230.0	2.13352
382.0	-0.585903	331.0	0.554089	280.0	2.90742	229.0	2.73056
381.0	-0.618581	330.0	0.626858	279.0	2.76713	228.0	3.30347
380.0	-0.639514	329.0	0.667793	278.0	2.63093	227.0	3.8318
379.0	-0.637204	328.0	0.706815	277.0	2.50734	226.0	4.32234
378.0	-0.630183	327.0	0.773817	276.0	2.38512	225.0	4.76614
377.0	-0.611935	326.0	0.880304	275.0	2.26663	224.0	5.16834
376.0	-0.622648	325.0	1.00593	274.0	2.12035	223.0	5.51605
375.0	-0.65789	324.0	1.1313	273.0	1.9462	222.0	5.84662
374.0	-0.722007	323.0	1.25395	272.0	1.761	221.0	6.15852
373.0	-0.788597	322.0	1.36278	271.0	1.57031	220.0	6.38923
372.0	-0.841731	321.0	1.47075	270.0	1.38311	219.0	6.47836
371.0	-0.866916	320.0	1.57686	269.0	1.18844	218.0	6.42077
370.0	-0.867611	319.0	1.67548	268.0	0.968748	217.0	6.32249
369.0	-0.853073	318.0	1.7855	267.0	0.732605	216.0	6.24645
368.0	-0.825506	317.0	1.89168	266.0	0.493759	215.0	6.14753
367.0	-0.814972	316.0	1.99891	265.0	0.261686	214.0	6.01141
366.0	-0.815264	315.0	2.09668	264.0	0.0616102	213.0	5.79963
365.0	-0.83965	314.0	2.18845	263.0	-0.0988238	212.0	5.54921
364.0	-0.862641	313.0	2.27215	262.0	-0.215624	211.0	5.22607
363.0	-0.864374	312.0	2.35348	261.0	-0.320429	210.0	4.83836
362.0	-0.853908	311.0	2.43882	260.0	-0.436395	209.0	4.47788
361.0	-0.829362	310.0	2.52944	259.0	-0.554616	208.0	4.10248
360.0	-0.812156	309.0	2.62369	258.0	-0.655245	207.0	3.60238
359.0	-0.791718	308.0	2.71407	257.0	-0.734182	206.0	3.13177
358.0	-0.767812	307.0	2.80069	256.0	-0.785758	205.0	2.70601
357.0	-0.748848	306.0	2.88918	255.0	-0.818027	204.0	2.23685
356.0	-0.742326	305.0	2.98288	254.0	-0.841675	203.0	1.7569
355.0	-0.745653	304.0	3.08108	253.0	-0.898223	202.0	1.31127
354.0	-0.739703	303.0	3.16876	252.0	-0.970006	201.0	0.924219
353.0	-0.717834	302.0	3.25205	251.0	-1.06005	200.0	0.601894
352.0	-0.669672	301.0	3.30672	250.0	-1.15759		
351.0	-0.625936	300.0	3.35809	249.0	-1.26376		
350.0	-0.584331	299.0	3.39432	248.0	-1.38234		

Table S2. The cartesian coordinates of conformer **1a**.

C	1.210336	-0.381653	0.362340	H	1.240980	3.063321	-0.467026
C	1.680880	0.933418	-0.357143	H	1.066276	2.392489	1.140849
C	0.877854	2.186527	0.081439	H	-0.771832	1.922287	-1.273311
C	-0.618355	2.020235	-0.190068	H	-1.153501	2.924523	0.126936
C	-1.201143	0.779962	0.525295	H	-0.450529	-0.532676	-0.969673
C	-0.350150	-0.486438	0.126138	H	1.501937	-1.607126	-1.431251
C	1.832292	-1.615648	-0.387836	H	1.465377	-2.532521	0.077009
C	1.593891	-0.463794	1.855507	H	1.038693	-1.271800	2.344224
C	-1.315699	1.069022	2.048359	H	2.657387	-0.665003	1.975879
C	-2.609093	0.506698	0.038924	H	1.379448	0.460887	2.392730
C	-3.123517	-0.712705	-0.185836	H	-1.604035	0.179967	2.614919
C	-2.388433	-2.005813	-0.030942	H	-0.385165	1.452231	2.469174
C	-1.032594	-1.768924	0.665241	H	-2.083579	1.830868	2.222162
C	-3.671838	1.534482	-0.243899	H	-2.232202	-2.456788	-1.021578
O	-4.826613	0.774556	-0.647837	H	-2.994012	-2.725302	0.534071
C	-4.520945	-0.563810	-0.624376	H	-0.410143	-2.652814	0.510538
C	3.339122	-1.746881	-0.361577	H	-1.188562	-1.692908	1.746821
O	3.992181	-2.239083	0.537168	H	-3.405537	2.215625	-1.059923
O	3.915538	-1.278724	-1.495323	H	-3.944270	2.137203	0.629939
C	3.159661	1.322681	-0.245229	H	4.880995	-1.395219	-1.396685
O	3.810605	1.168911	0.777032	H	3.103109	2.768726	-1.867322
C	3.763573	1.992397	-1.466086	H	3.893740	1.240459	-2.252649
O	-5.337383	-1.409918	-0.929262	H	4.734272	2.425039	-1.214111
H	1.471472	0.794066	-1.427454				

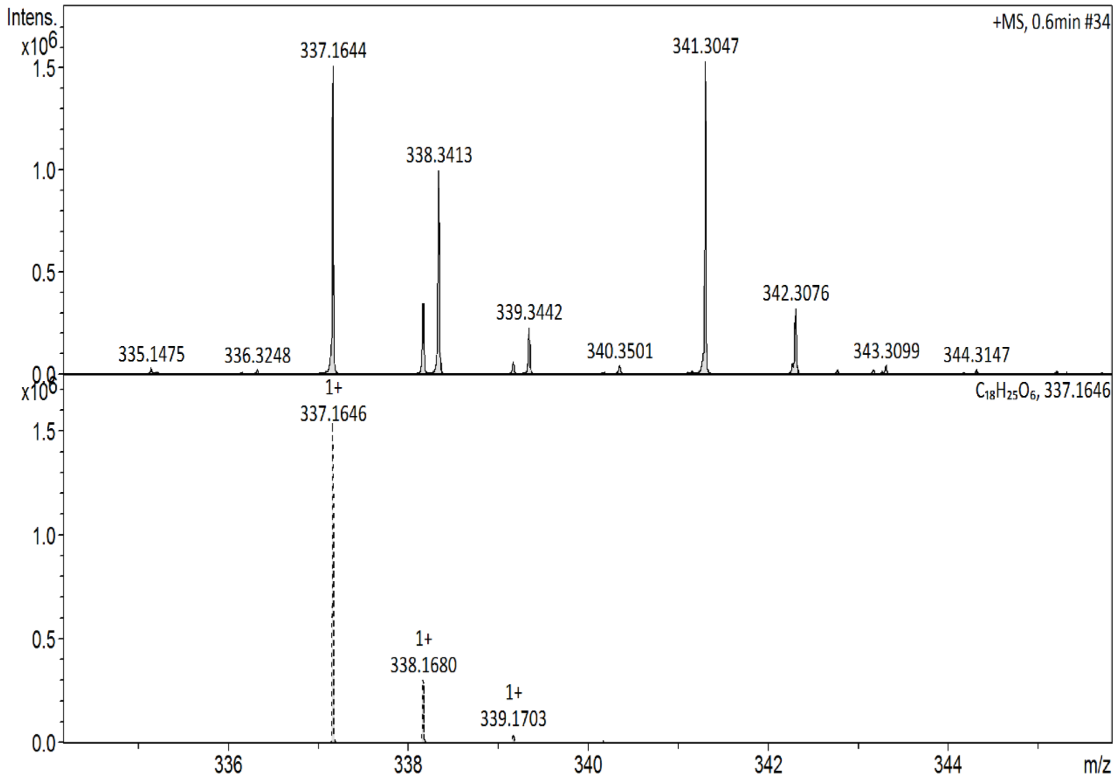

Figure S10. HRESIMS spectrum of **2**.

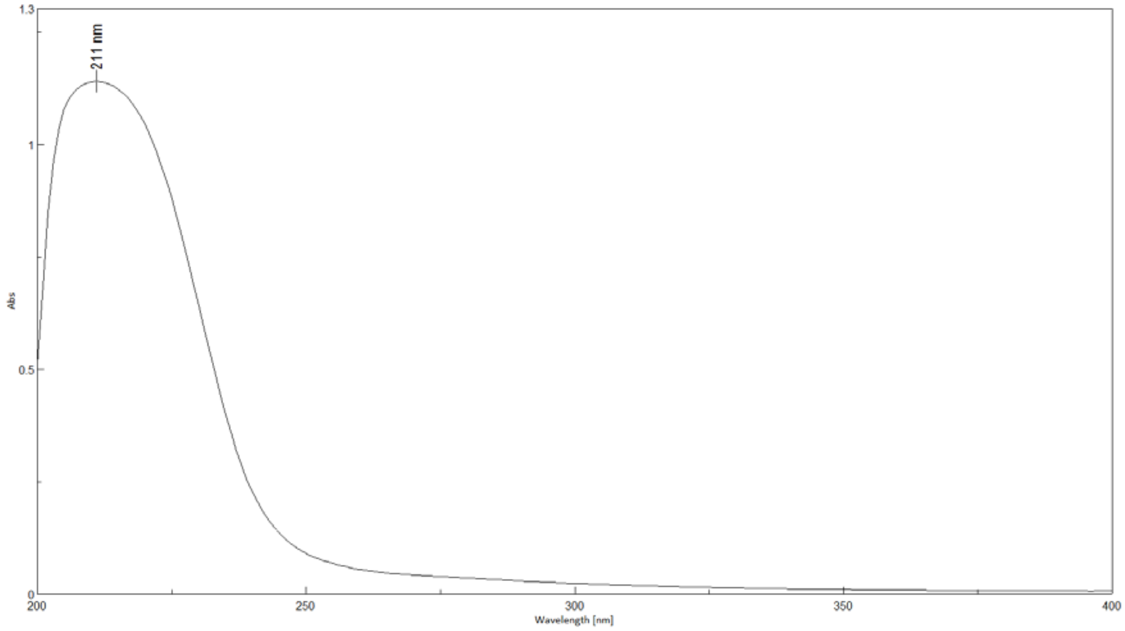

Figure S11. UV spectrum of **2**.

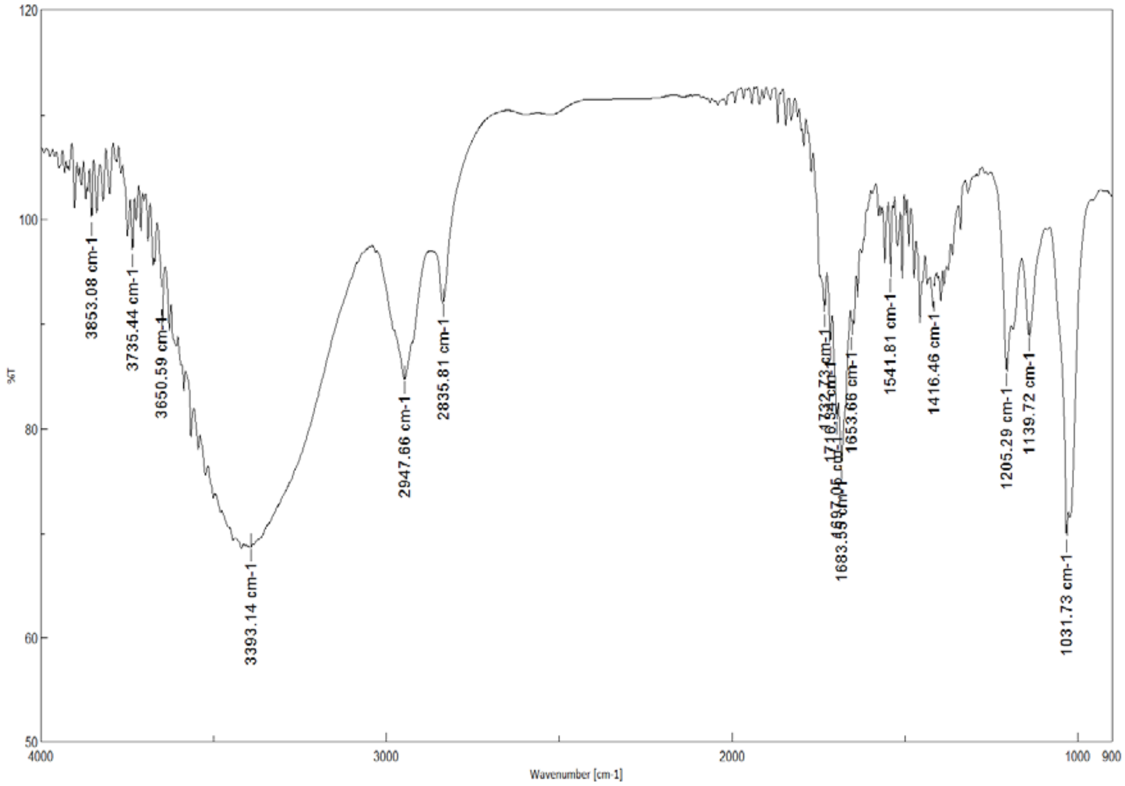

Figure S12. IR spectrum of **2**.

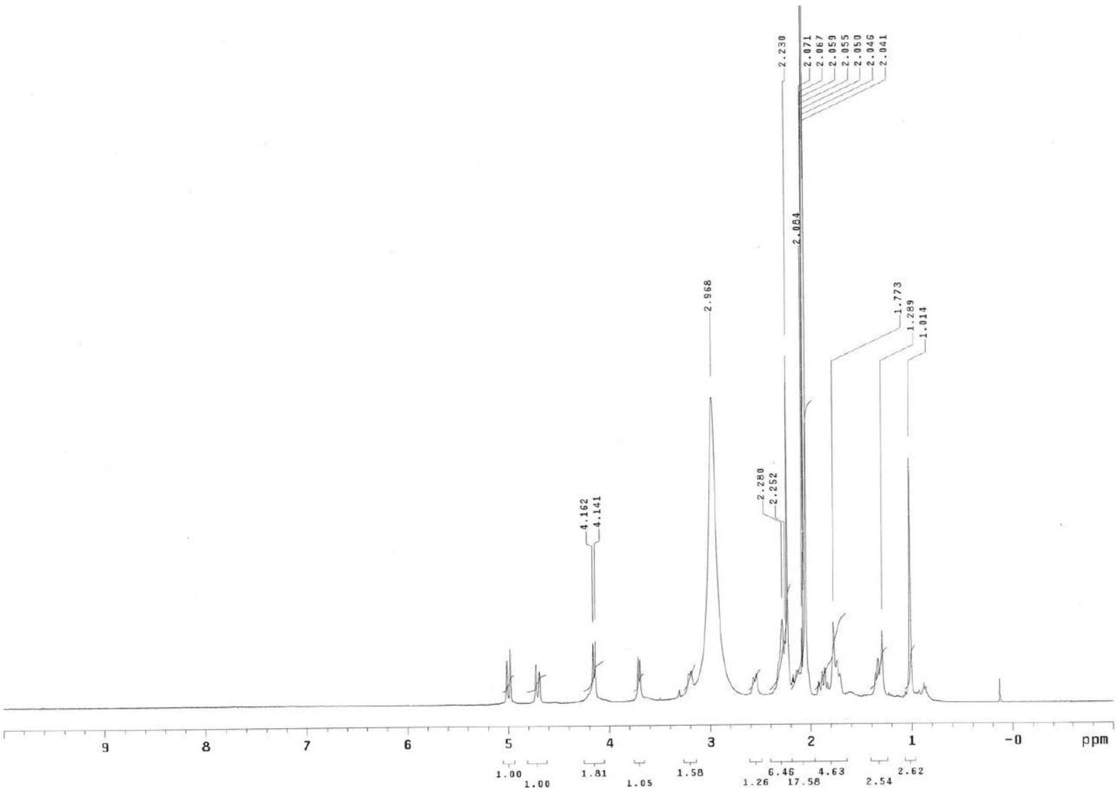

Figure S13. ^{1}H NMR spectrum of **2** in acetone-d_6.

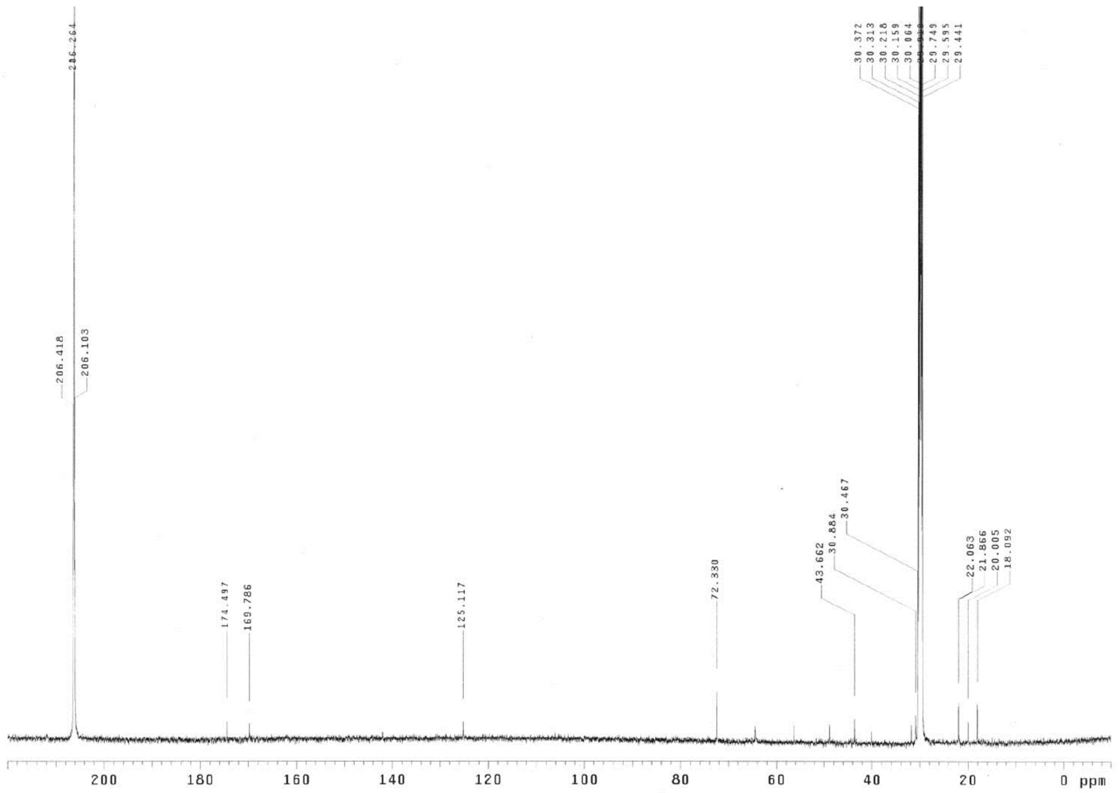

Figure S14. ^{13}C NMR spectrum of **2** in acetone-d_6.

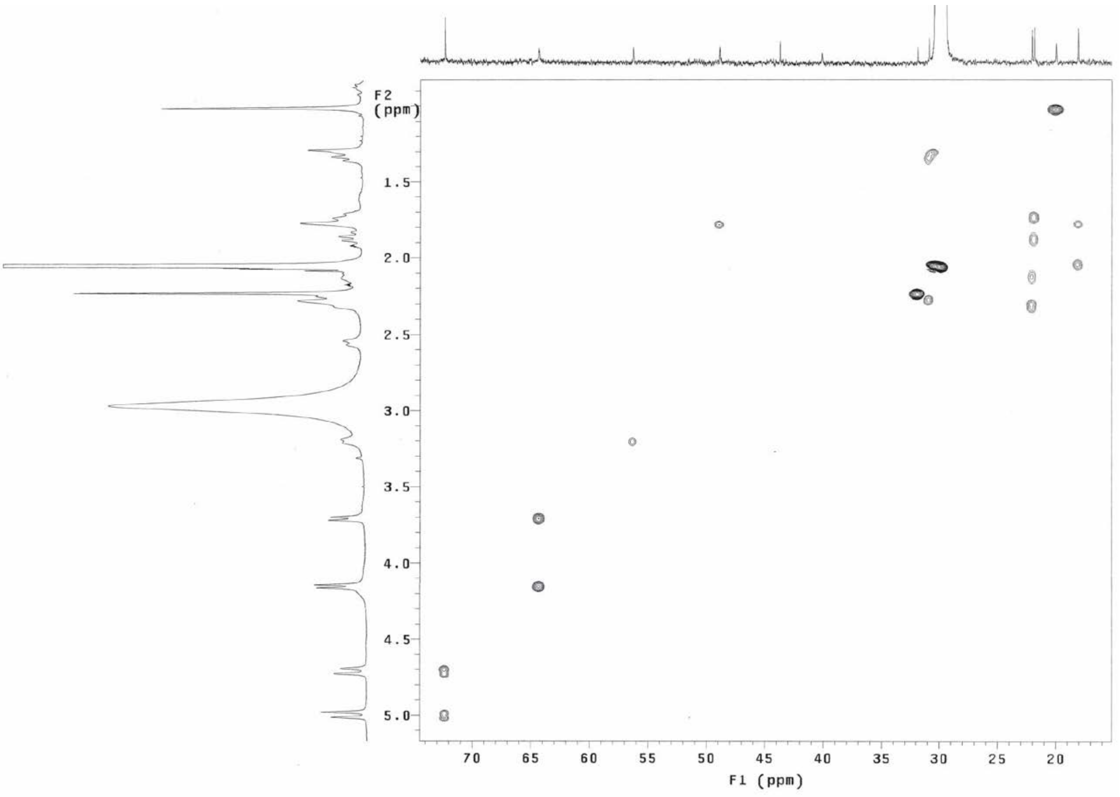

Figure S15. HSQC spectrum of **2** in acetone-d_6.

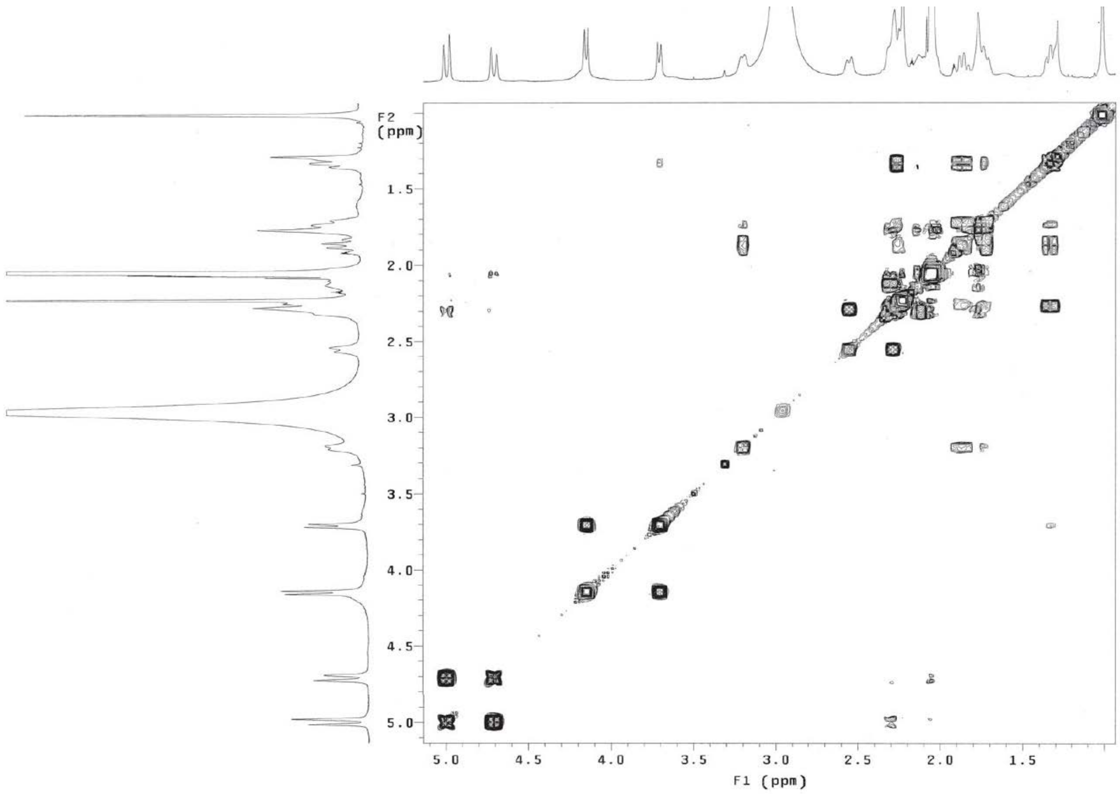

Figure S16. ^{1}H–^{1}H COSY spectrum of **2** in acetone-d_6.

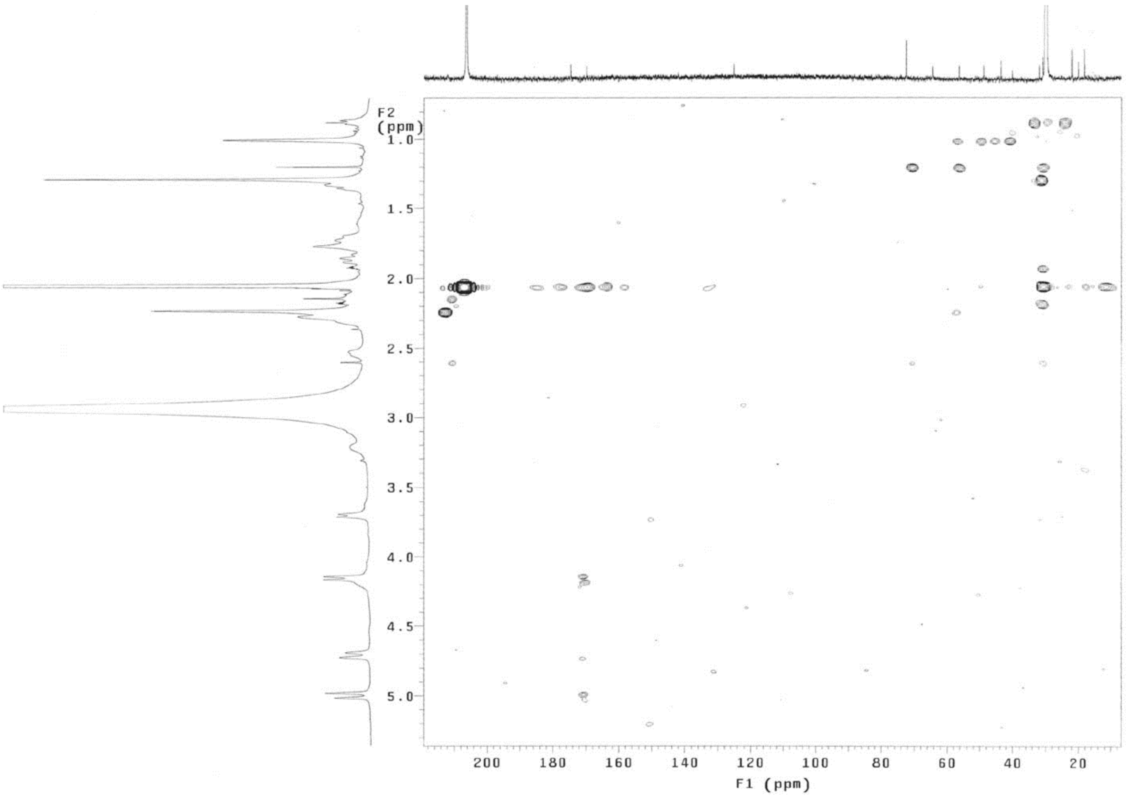

Figure S17. HMBC spectrum of **2** in acetone-d_6.

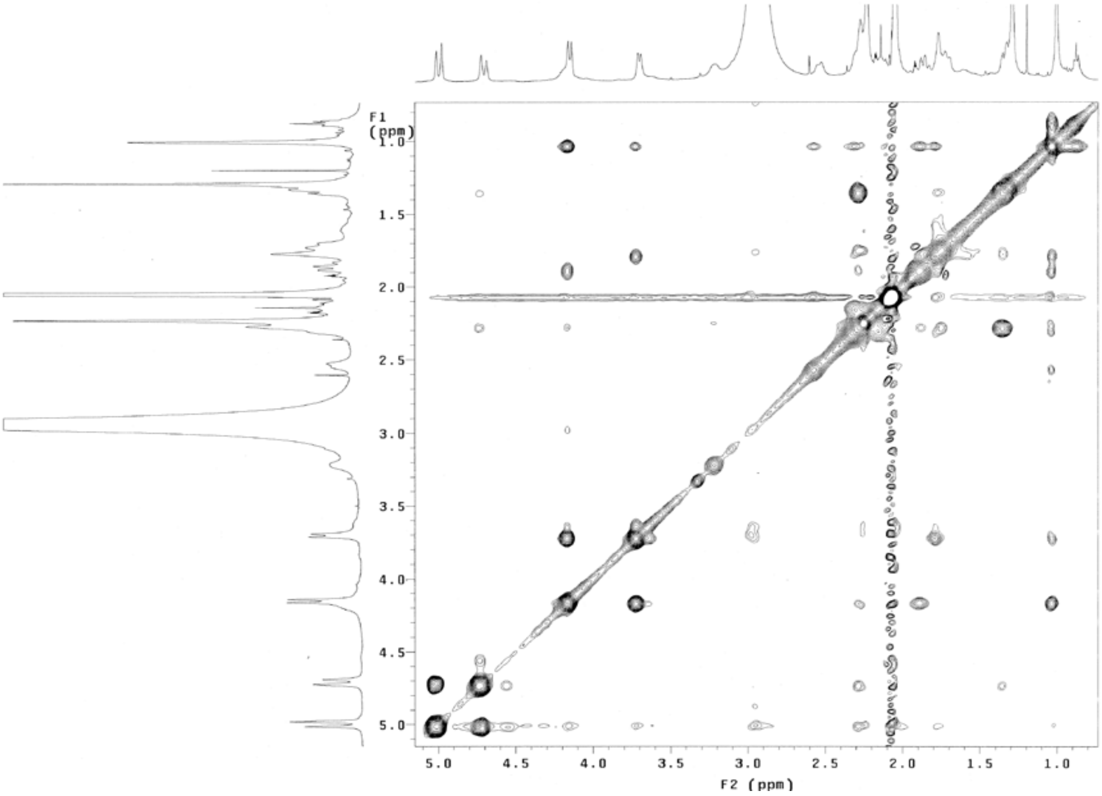

Figure S18. NOESY spectrum of **2** in acetone-d_6.

Table S3. The CD experimental data of **2**.

Wavelength [nm]	CD [mdeg]	Wavelength [nm]	CD [mdeg]	Wavelength [nm]	CD [mdeg]	Wavelength [nm]	CD [mdeg]
400.0	0.321834	349.0	-0.780443	298.0	3.94397	247.0	0.265689
399.0	0.281808	348.0	-0.739962	297.0	4.01863	246.0	0.210737
398.0	0.235798	347.0	-0.693495	296.0	4.08368	245.0	0.117652
397.0	0.182394	346.0	-0.641812	295.0	4.14073	244.0	-0.0194541
396.0	0.121131	345.0	-0.587366	294.0	4.18927	243.0	-0.209395
395.0	0.051804	344.0	-0.530862	293.0	4.22646	242.0	-0.455755
394.0	-0.023197	343.0	-0.471363	292.0	4.2536	241.0	-0.762065
393.0	-0.100854	342.0	-0.407469	291.0	4.27026	240.0	-1.12468
392.0	-0.174889	341.0	-0.338232	290.0	4.27674	239.0	-1.53687
391.0	-0.242736	340.0	-0.264019	289.0	4.27002	238.0	-1.99299
390.0	-0.303599	339.0	-0.184946	288.0	4.24539	237.0	-2.48921
389.0	-0.360054	338.0	-0.1029	287.0	4.20218	236.0	-3.02614
388.0	-0.412826	337.0	-0.0194129	286.0	4.14359	235.0	-3.59829
387.0	-0.461962	336.0	0.0626986	285.0	4.07261	234.0	-4.19747
386.0	-0.507356	335.0	0.141214	284.0	3.98792	233.0	-4.81
385.0	-0.54598	334.0	0.215831	283.0	3.88772	232.0	-5.42663
384.0	-0.576968	333.0	0.288237	282.0	3.77089	231.0	-6.03498
383.0	-0.599306	332.0	0.360769	281.0	3.64102	230.0	-6.61962
382.0	-0.614555	331.0	0.434886	280.0	3.4989	229.0	-7.16652
381.0	-0.626801	330.0	0.510199	279.0	3.34329	228.0	-7.66671
380.0	-0.639668	329.0	0.585194	278.0	3.17539	227.0	-8.12448
379.0	-0.655624	328.0	0.658681	277.0	2.99581	226.0	-8.54031
378.0	-0.674501	327.0	0.730365	276.0	2.80794	225.0	-8.91048
377.0	-0.693852	326.0	0.802864	275.0	2.61477	224.0	-9.22001
376.0	-0.711869	325.0	0.878649	274.0	2.41434	223.0	-9.45525
375.0	-0.729473	324.0	0.959893	273.0	2.20486	222.0	-9.60893
374.0	-0.746755	323.0	1.04702	272.0	1.98611	221.0	-9.68425
373.0	-0.765191	322.0	1.13907	271.0	1.76174	220.0	-9.68701
372.0	-0.785577	321.0	1.23562	270.0	1.53567	219.0	-9.61849
371.0	-0.807242	320.0	1.33654	269.0	1.31222	218.0	-9.48579
370.0	-0.830578	319.0	1.44036	268.0	1.09548	217.0	-9.2965
369.0	-0.852877	318.0	1.54593	267.0	0.891854	216.0	-9.06835
368.0	-0.872877	317.0	1.65397	266.0	0.703519	215.0	-8.79414
367.0	-0.891066	316.0	1.76574	265.0	0.531137	214.0	-8.46428
366.0	-0.908578	315.0	1.88354	264.0	0.376689	213.0	-8.07931
365.0	-0.927014	314.0	2.00738	263.0	0.240965	212.0	-7.6789
364.0	-0.945189	313.0	2.13522	262.0	0.126717	211.0	-7.29316
363.0	-0.961182	312.0	2.26486	261.0	0.0369944	210.0	-6.94228
362.0	-0.973482	311.0	2.39527	260.0	-0.0260089	209.0	-6.64216
361.0	-0.981941	310.0	2.52761	259.0	-0.0623904	208.0	-6.39592
360.0	-0.986627	309.0	2.66237	258.0	-0.0745093	207.0	-6.21045
359.0	-0.987451	308.0	2.79897	257.0	-0.0646119	206.0	-6.08366
358.0	-0.98387	307.0	2.93442	256.0	-0.036118	205.0	-6.00062
357.0	-0.976123	306.0	3.069	255.0	0.00536826	204.0	-5.94813
356.0	-0.965294	305.0	3.20039	254.0	0.0542515	203.0	-5.92734
355.0	-0.950619	304.0	3.32764	253.0	0.10904	202.0	-5.94802
354.0	-0.931556	303.0	3.44802	252.0	0.165277	201.0	-6.0187
353.0	-0.907426	302.0	3.5605	251.0	0.217348	200.0	-6.12177
352.0	-0.878559	301.0	3.66618	250.0	0.259522		
351.0	-0.848046	300.0	3.76607	249.0	0.285166		
350.0	-0.815334	299.0	3.85952	248.0	0.289524		

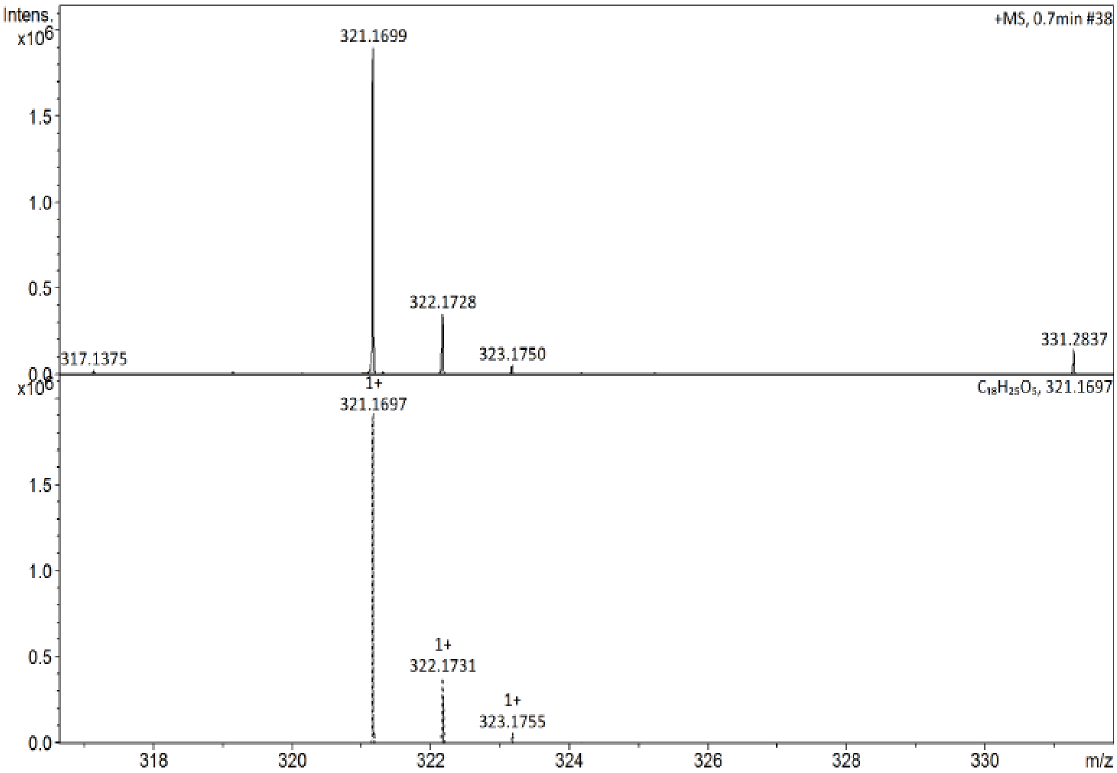

Figure S19. HRESIMS spectrum of **3**.

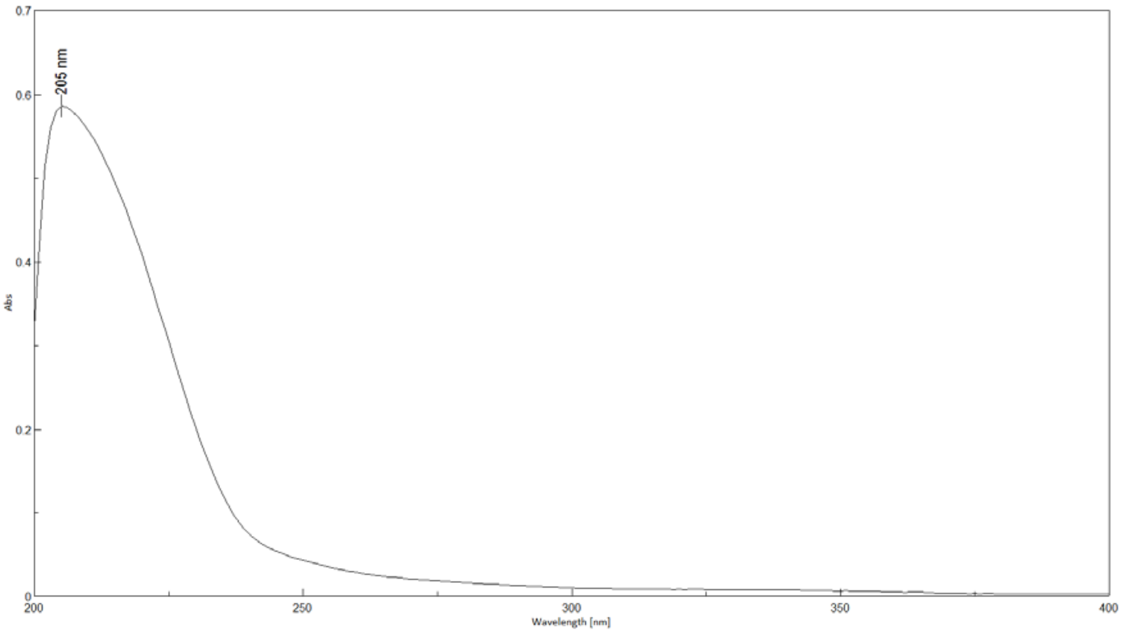

Figure S20. UV spectrum of **3**.

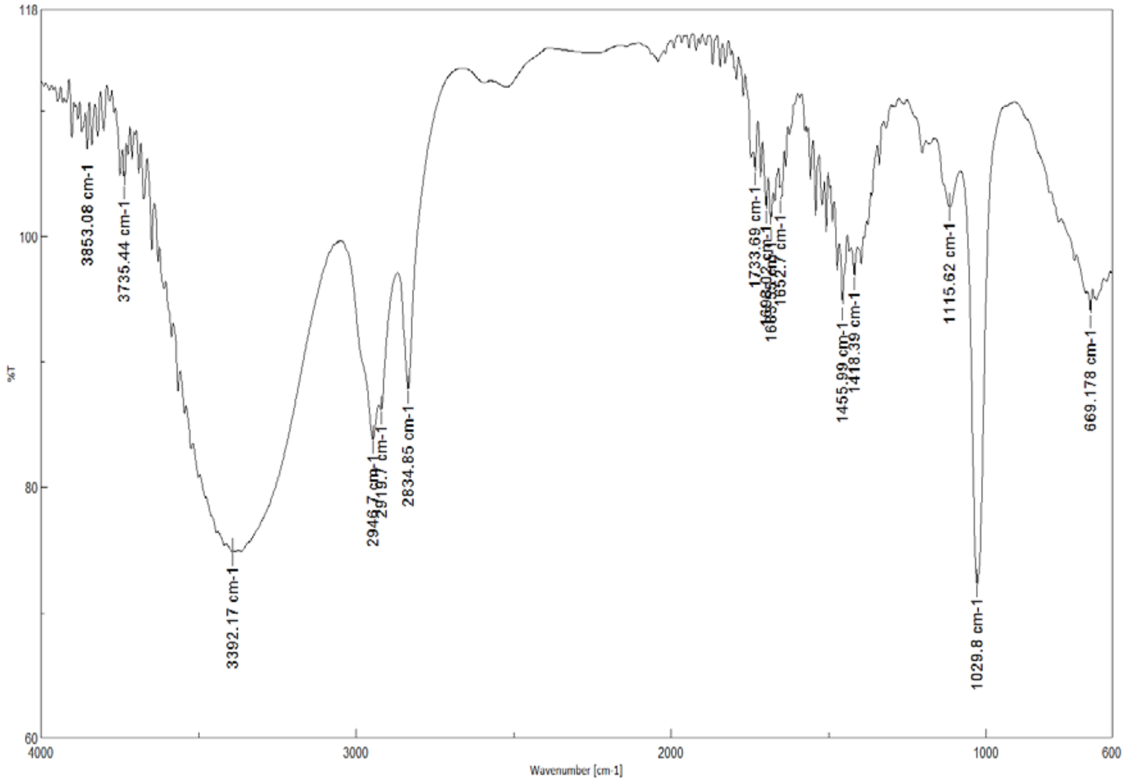

Figure S21. IR spectrum of **3**.

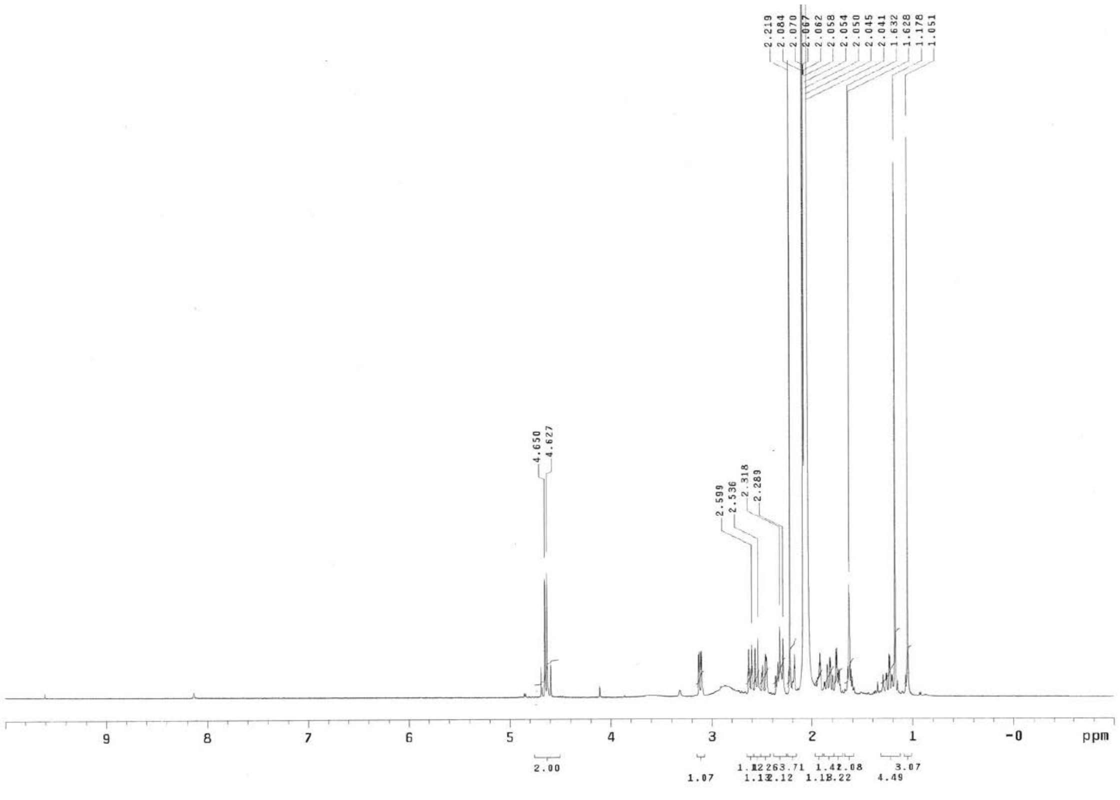

Figure S22. ^{1}H NMR spectrum of **3** in acetone-d_6.

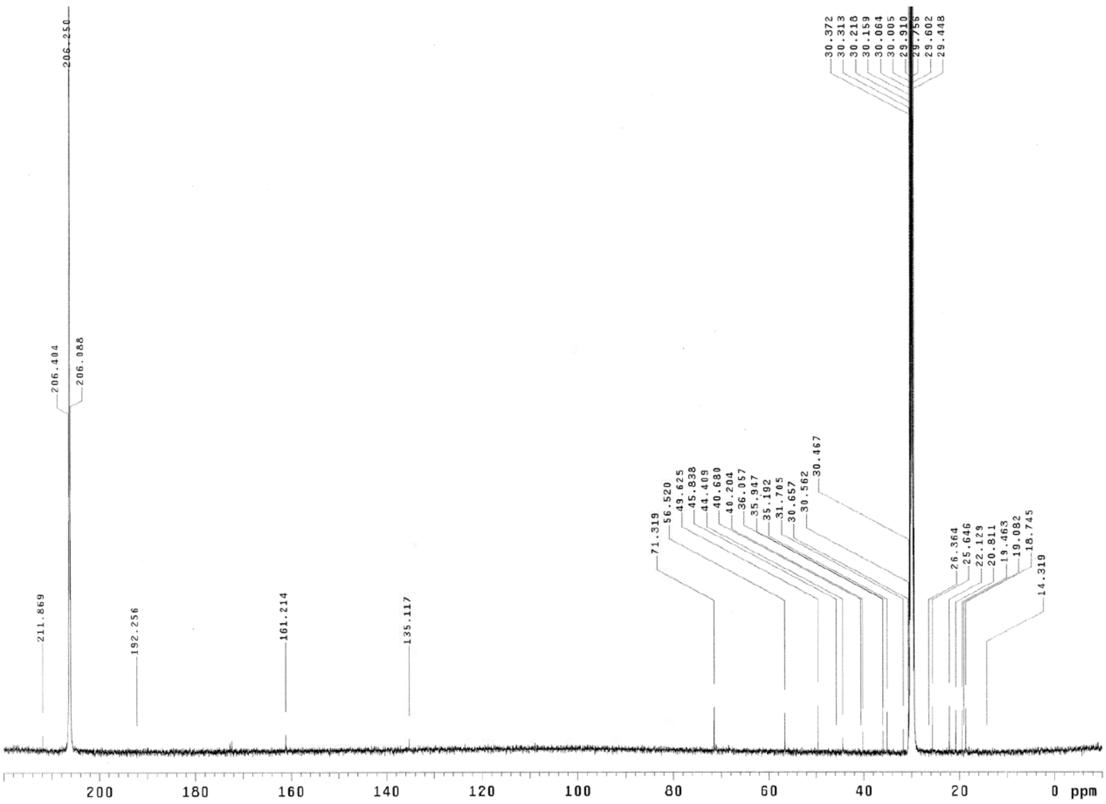

Figure S23. ^{13}C NMR spectrum of **3** in acetone-d_6.

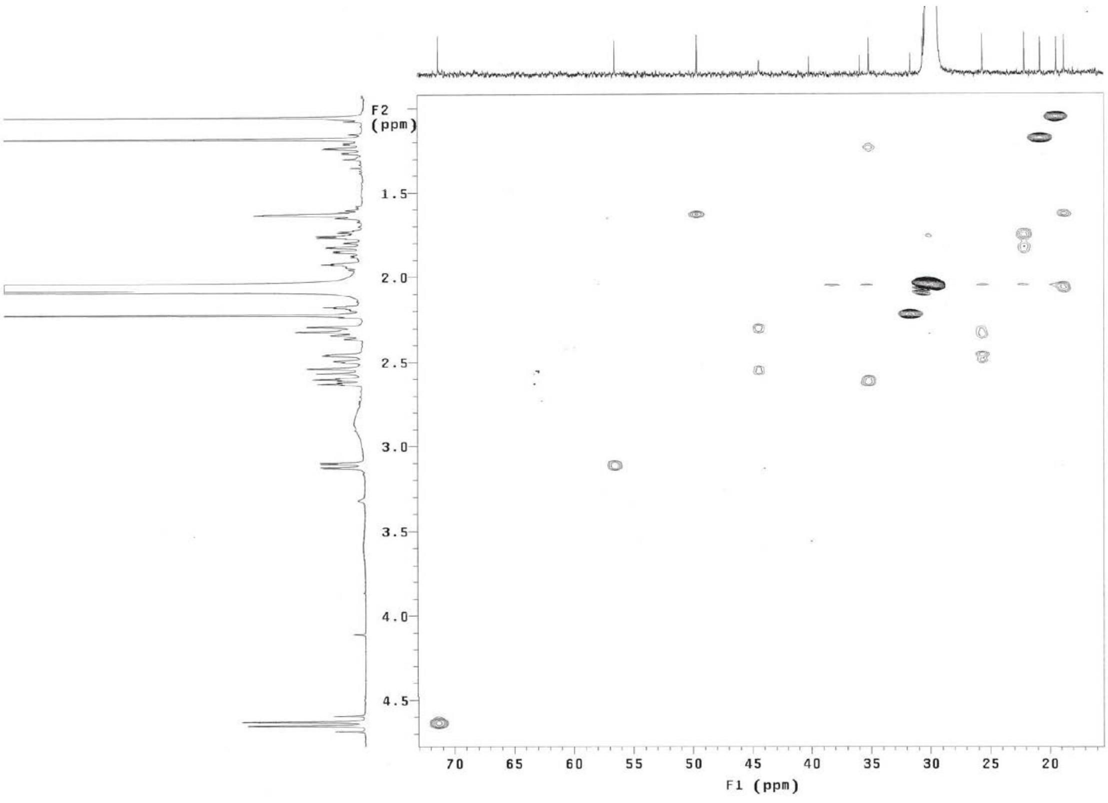

Figure S24. HSQC spectrum of **3** in acetone-d_6.

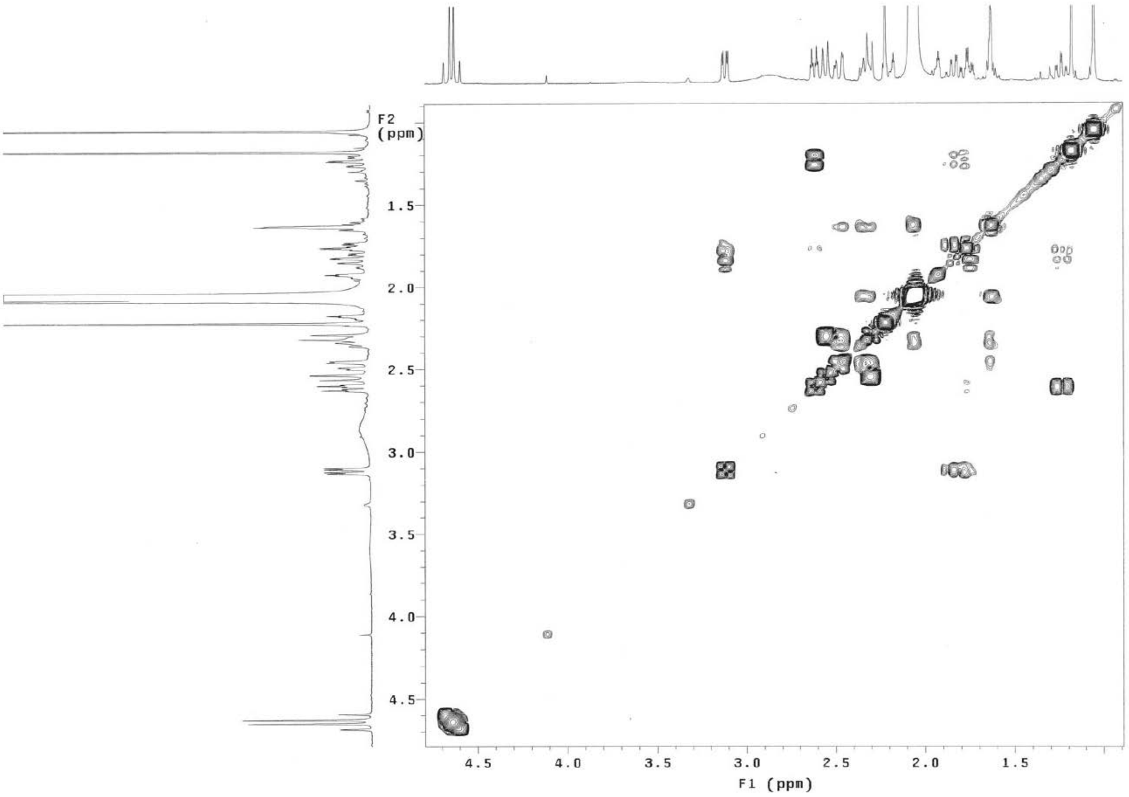

Figure S25. $^{1}H-^{1}H$ COSY spectrum of **3** in acetone-d_6.

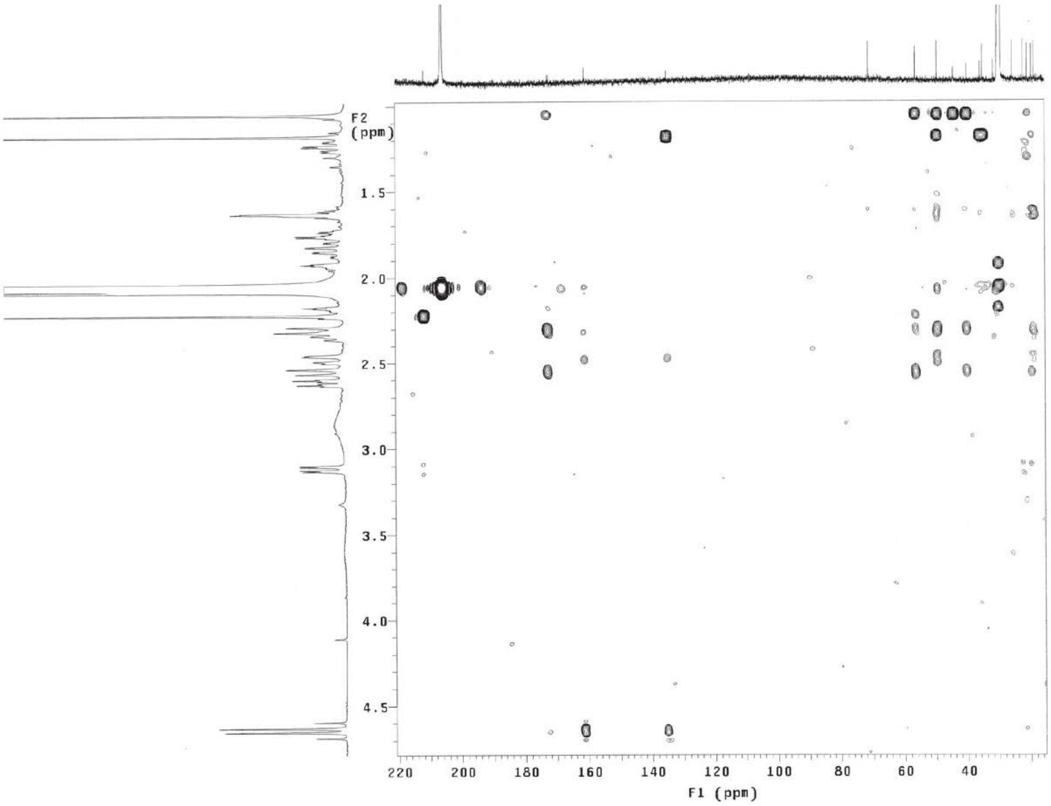

Figure S26. HMBC spectrum of **3** in acetone-d_6.

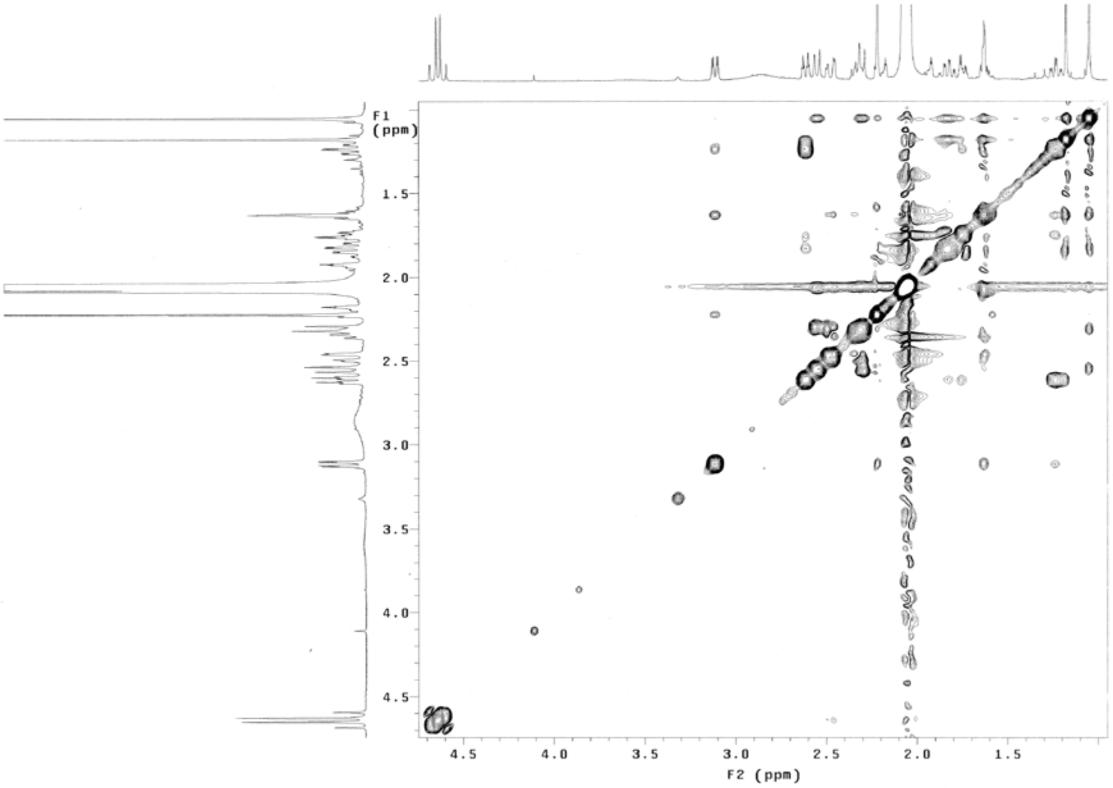

Figure S27. NOESY spectrum of **3** in acetone-d_6.

Table S4. The CD experimental data of **3**.

Wavelength [nm]	CD [mdeg]	Wavelength [nm]	CD [mdeg]	Wavelength [nm]	CD [mdeg]	Wavelength [nm]	CD [mdeg]
400.0	-0.1348565	375.2	0.079924	350.4	-0.116589	325.6	0.374704
399.6	-0.1385035	374.8	0.0811835	350.0	-0.0991795	325.2	0.380213
399.2	-0.142054	374.4	0.0816875	349.6	-0.081573	324.8	0.38574
398.8	-0.145329	374.0	0.0814345	349.2	-0.063995	324.4	0.391231
398.4	-0.1485295	373.6	0.0804665	348.8	-0.0465431	324.0	0.396739
398.0	-0.151895	373.2	0.0788605	348.4	-0.02913495	323.6	0.40225
397.6	-0.155572	372.8	0.0768675	348.0	-0.0117869	323.2	0.407716
397.2	-0.1596055	372.4	0.074548	347.6	0.00554835	322.8	0.412997
396.8	-0.163753	372.0	0.0716425	347.2	0.02308	322.4	0.41804
396.4	-0.167604	371.6	0.0679835	346.8	0.0408337	322.0	0.4227595
396.0	-0.170938	371.2	0.06338	346.4	0.058686	321.6	0.427159
395.6	-0.17372	370.8	0.0577685	346.0	0.076577	321.2	0.4313865
395.2	-0.175912	370.4	0.051325	345.6	0.094263	320.8	0.4354435
394.8	-0.1775605	370.0	0.0440428	345.2	0.111612	320.4	0.4391395
394.4	-0.17856	369.6	0.0359071	344.8	0.1286015	320.0	0.4422445
394.0	-0.1787315	369.2	0.0269872	344.4	0.14525	319.6	0.444615
393.6	-0.1780785	368.8	0.01734705	344.0	0.161683	319.2	0.4461535
393.2	-0.176545	368.4	0.0072983	343.6	0.1779165	318.8	0.4469605
392.8	-0.174163	368.0	-0.00292732	343.2	0.1938525	318.4	0.4473735
392.4	-0.1710045	367.6	-0.01335245	342.8	0.209318	318.0	0.44751
392.0	-0.167065	367.2	-0.02388825	342.4	0.2240105	317.6	0.447438
391.6	-0.162437	366.8	-0.03453715	342.0	0.237801	317.2	0.4470515
391.2	-0.157323	366.4	-0.0454026	341.6	0.250719	316.8	0.446182
390.8	-0.1518775	366.0	-0.056444	341.2	0.262757	316.4	0.444822
390.4	-0.14635	365.6	-0.0676895	340.8	0.2739875	316.0	0.443062
390.0	-0.140953	365.2	-0.0790835	340.4	0.2844935	315.6	0.4409385
389.6	-0.135704	364.8	-0.090434	340.0	0.2942135	315.2	0.4384565
389.2	-0.1305825	364.4	-0.1015145	339.6	0.303072	314.8	0.435686
388.8	-0.1254875	364.0	-0.1122975	339.2	0.3111215	314.4	0.4324895
388.4	-0.1202495	363.6	-0.122848	338.8	0.3184245	314.0	0.4288025
388.0	-0.1147815	363.2	-0.133313	338.4	0.325052	313.6	0.4246495
387.6	-0.1089425	362.8	-0.1439115	338.0	0.331235	313.2	0.419952
387.2	-0.102573	362.4	-0.154606	337.6	0.3370175	312.8	0.4146865
386.8	-0.0955995	362.0	-0.165262	337.2	0.342125	312.4	0.40887
386.4	-0.08795	361.6	-0.1757225	336.8	0.346289	312.0	0.402424
386.0	-0.079837	361.2	-0.1857545	336.4	0.349362	311.6	0.3951935
385.6	-0.071547	360.8	-0.1952955	336.0	0.3512405	311.2	0.3870685
385.2	-0.063219	360.4	-0.2044145	335.6	0.352168	310.8	0.377891
384.8	-0.0548805	360.0	-0.213154	335.2	0.352547	310.4	0.3676325
384.4	-0.0464725	359.6	-0.2214055	334.8	0.352517	310.0	0.356479
384.0	-0.03804955	359.2	-0.2290735	334.4	0.352242	309.6	0.3444365
383.6	-0.0298201	358.8	-0.235961	334.0	0.351819	309.2	0.3315465
383.2	-0.02197295	358.4	-0.2418195	333.6	0.351095	308.8	0.3180405
382.8	-0.0145037	358.0	-0.246602	333.2	0.3499845	308.4	0.3040185
382.4	-0.0073196	357.6	-0.250336	332.8	0.348476	308.0	0.2897285
382.0	-0.000322778	357.2	-0.2529765	332.4	0.3466775	307.6	0.275536
381.6	0.0064992	356.8	-0.2546195	332.0	0.3447595	307.2	0.261599
381.2	0.0130746	356.4	-0.2552855	331.6	0.342937	306.8	0.248064
380.8	0.01931915	356.0	-0.2549845	331.2	0.341434	306.4	0.2350975
380.4	0.025229	355.6	-0.2536555	330.8	0.340398	306.0	0.222921
380.0	0.03084165	355.2	-0.251188	330.4	0.3398905	305.6	0.211808
379.6	0.03620495	354.8	-0.2475505	330.0	0.339988	305.2	0.2019925
379.2	0.04144705	354.4	-0.242573	329.6	0.3407875	304.8	0.1935845
378.8	0.04666155	354.0	-0.2361725	329.2	0.342295	304.4	0.186694
378.4	0.051862	353.6	-0.2282705	328.8	0.344395	304.0	0.1814145
378.0	0.0569785	353.2	-0.2187635	328.4	0.346931	303.6	0.177761
377.6	0.061814	352.8	-0.207631	328.0	0.349747	303.2	0.175876
377.2	0.066131	352.4	-0.194976	327.6	0.3528365	302.8	0.17576
376.8	0.069862	352.0	-0.18103	327.2	0.35625	302.4	0.1774385
376.4	0.073053	351.6	-0.1660425	326.8	0.3601285	302.0	0.181106
376.0	0.0757505	351.2	-0.150205	326.4	0.364584	301.6	0.1869105
375.6	0.0780505	350.8	-0.1336695	326.0	0.369478	301.2	0.1949645

Wavelength [nm]	CD [mdeg]
300.8	0.2053475
300.4	0.217909
300.0	0.232467
299.6	0.2488865
299.2	0.267033
298.8	0.2869135
298.4	0.308616
298.0	0.3319835
297.6	0.3569105
297.2	0.383307
296.8	0.4110835
296.4	0.440397
296.0	0.471419
295.6	0.50414
295.2	0.538595
294.8	0.57476
294.4	0.612505
294.0	0.651655
293.6	0.69199
293.2	0.733315
292.8	0.775465
292.4	0.8182
292.0	0.861225
291.6	0.904205
291.2	0.94687
290.8	0.988945
290.4	1.03036
290.0	1.0711
289.6	1.111075
289.2	1.15013
288.8	1.187915
288.4	1.22404
288.0	1.258215
287.6	1.290245
287.2	1.32002
286.8	1.34742
286.4	1.372305
286.0	1.39448
285.6	1.41377
285.2	1.43011
284.8	1.443405
284.4	1.45353
284.0	1.460495
283.6	1.46421
283.2	1.464405
282.8	1.46085
282.4	1.4534
282.0	1.44207
281.6	1.426845
281.2	1.407825
280.8	1.38522
280.4	1.35924
280.0	1.33023
279.6	1.29848
279.2	1.264135
278.8	1.22705
278.4	1.18721
278.0	1.14471
277.6	1.09965
277.2	1.0522
276.8	1.00255
276.4	0.95101
276.0	0.8981
275.6	0.84447
275.2	0.79077
274.8	0.73744
274.4	0.68471
274.0	0.63268
273.6	0.581555
273.2	0.53158
272.8	0.4830645
272.4	0.4365185
272.0	0.392266
271.6	0.3505425
271.2	0.311777
270.8	0.2761345
270.4	0.244058
270.0	0.2159455
269.6	0.191926
269.2	0.172046
268.8	0.1562815
268.4	0.1446915
268.0	0.13749
267.6	0.1350995
267.2	0.137727
266.8	0.1452935
266.4	0.1574845
266.0	0.1738365
265.6	0.194018
265.2	0.2176555
264.8	0.244365
264.4	0.273979
264.0	0.3062555
263.6	0.34093
263.2	0.3776745
262.8	0.415957
262.4	0.4551735
262.0	0.4947115
261.6	0.53413
261.2	0.5732
260.8	0.611695
260.4	0.649175
260.0	0.68522
259.6	0.719365
259.2	0.75109
258.8	0.77997
258.4	0.805715
258.0	0.82806
257.6	0.84664
257.2	0.861115
256.8	0.871265
256.4	0.876785
256.0	0.877265
255.6	0.87235
255.2	0.8618
254.8	0.84519
254.4	0.82232
254.0	0.793525
253.6	0.759135
253.2	0.719235
252.8	0.67403
252.4	0.622045
252.0	0.562245
251.6	0.4942115
251.2	0.417739
250.8	0.3325485
250.4	0.238399
250.0	0.135408
249.6	0.0238549
249.2	-0.095866
248.8	-0.2232155
248.4	-0.357766
248.0	-0.498989
247.6	-0.646095
247.2	-0.798225
246.8	-0.95434
246.4	-1.113055
246.0	-1.273015
245.6	-1.43262
245.2	-1.59021
244.8	-1.744235
244.4	-1.893225
244.0	-2.03575
243.6	-2.170135
243.2	-2.294505
242.8	-2.40703
242.4	-2.505945
242.0	-2.589515
241.6	-2.656175
241.2	-2.704455
240.8	-2.73255
240.4	-2.738645
240.0	-2.721225
239.6	-2.678535
239.2	-2.60897
238.8	-2.511355
238.4	-2.38443
238.0	-2.227295
237.6	-2.03937
237.2	-1.82033
236.8	-1.570415
236.4	-1.289875
236.0	-0.979315
235.6	-0.63958
235.2	-0.27125
234.8	0.124685
234.4	0.546725
234.0	0.99322
233.6	1.46191
233.2	1.950515
232.8	2.45673
232.4	2.97798
232.0	3.511795
231.6	4.055265
231.2	4.60502
230.8	5.1576
230.4	5.7088
230.0	6.2541
229.6	6.7893
229.2	7.3105
228.8	7.81405
228.4	8.29605
228.0	8.75245
227.6	9.17885
227.2	9.57105
226.8	9.92515
226.4	10.23745
226.0	10.50435
225.6	10.7219
225.2	10.8868
224.8	10.99635
224.4	11.0483
224.0	11.04105
223.6	10.9733
223.2	10.8436
222.8	10.65065
222.4	10.3931
222.0	10.07005
221.6	9.6811
221.2	9.2262
220.8	8.70635
220.4	8.1232
220.0	7.47895
219.6	6.77585
219.2	6.01675
218.8	5.20455
218.4	4.341795
218.0	3.43161
217.6	2.477055
217.2	1.48128
216.8	0.4489925
216.4	-0.614
216.0	-1.70171
215.6	-2.807505
215.2	-3.92489
214.8	-5.04775
214.4	-6.1701
214.0	-7.2855
213.6	-8.3874
213.2	-9.4692
212.8	-10.52445
212.4	-11.54665
212.0	-12.52995
211.6	-13.4688
211.2	-14.3573
210.8	-15.1903
210.4	-15.9622
210.0	-16.66725
209.6	-17.29955
209.2	-17.8532
208.8	-18.3222
208.4	-18.7023
208.0	-18.9903
207.6	-19.18335
207.2	-19.27935
206.8	-19.2759
206.4	-19.17085
206.0	-18.96235
205.6	-18.64965
205.2	-18.23395
204.8	-17.71715
204.4	-17.10305
204.0	-16.39555
203.6	-15.59735
203.2	-14.71115
202.8	-13.7417
202.4	-12.6945
202.0	-11.5731
201.6	-10.38175
201.2	-9.12785
200.8	-7.81705
200.4	-6.47415
200.0	-5.11535

Table S5. The cartesian coordinates of conformer **3a**.

C	1.196291	-0.419356	0.344544	H	0.726997	2.286467	1.228039
C	1.544300	0.968350	-0.305244	H	0.871205	3.039475	-0.346490
C	0.592955	2.105367	0.155505	H	-0.979841	1.714956	-1.262609
C	-0.868161	1.790882	-0.171719	H	-1.507253	2.617573	0.153768
C	-1.337868	0.470919	0.482819	H	-0.404370	-0.677359	-1.044623
C	-0.339600	-0.675293	0.055396	H	-1.931812	-2.759263	-1.271244
C	-2.697697	0.055928	-0.057713	H	-2.715360	-3.221734	0.228122
C	-3.062401	-1.200573	-0.376111	H	-0.172087	-2.854525	0.350761
C	-2.178110	-2.398133	-0.260815	H	-1.105020	-2.040001	1.585237
C	-0.890805	-2.048314	0.511064	H	-4.590862	-1.530827	-1.891160
C	-3.869690	0.931690	-0.268908	H	-5.142850	-1.813580	-0.221249
O	-4.904028	0.170313	-0.753659	H	1.649846	-1.533998	-1.487459
C	-4.482459	-1.206076	-0.850138	H	1.679862	-2.520140	-0.017703
C	1.960184	-1.552855	-0.437791	H	4.993829	-0.975504	-1.380000
C	3.470830	-1.538551	-0.388019	H	4.400373	2.871849	-1.003397
O	4.021222	-0.944579	-1.474111	H	2.760980	3.001002	-1.713189
C	2.963603	1.519478	-0.137376	H	3.778316	1.617958	-2.111644
C	3.511413	2.313812	-1.307149	H	2.620073	-0.601844	1.996558
O	-4.017071	2.126991	-0.077444	H	1.194722	0.329208	2.417173
O	3.610528	1.392632	0.895451	H	1.082608	-1.428366	2.272964
O	4.155876	-2.030375	0.491637	H	-1.718053	-0.249200	2.538435
C	1.543210	-0.531021	1.844589	H	-0.652069	1.160234	2.475062
C	-1.518982	0.687710	2.010112	H	-2.376197	1.349298	2.178471
H	1.382675	0.854694	-1.387067				

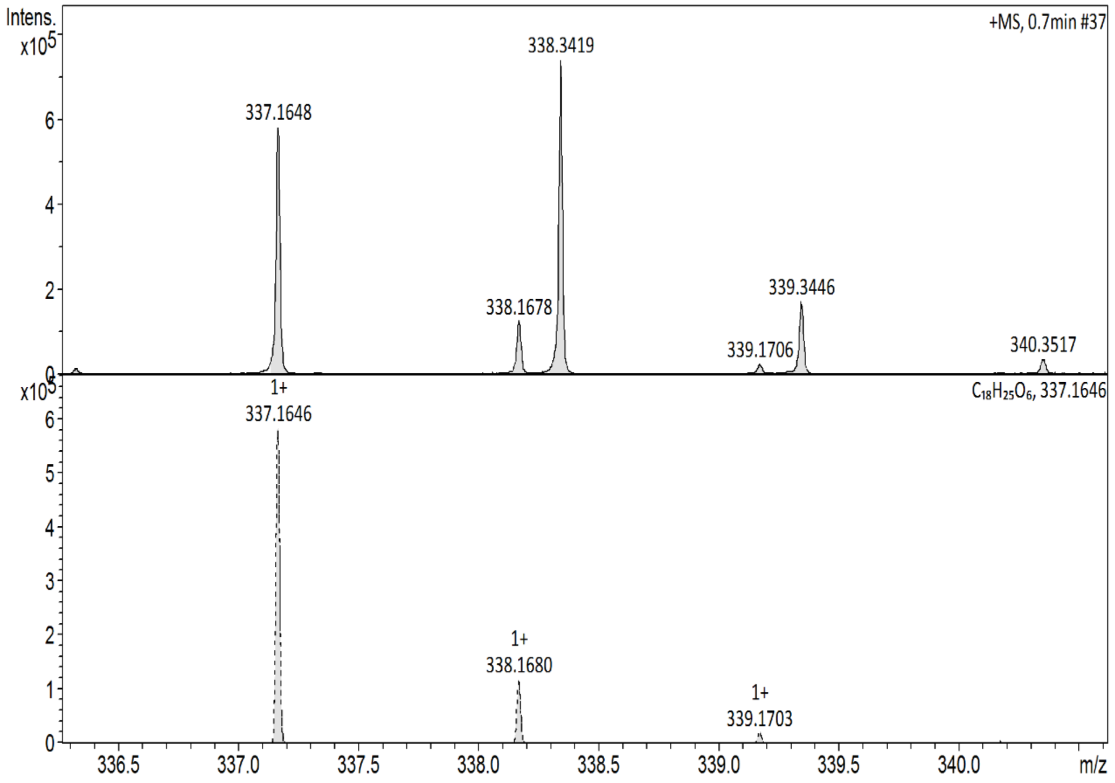

Figure S28. HRESIMS spectrum of **4**.

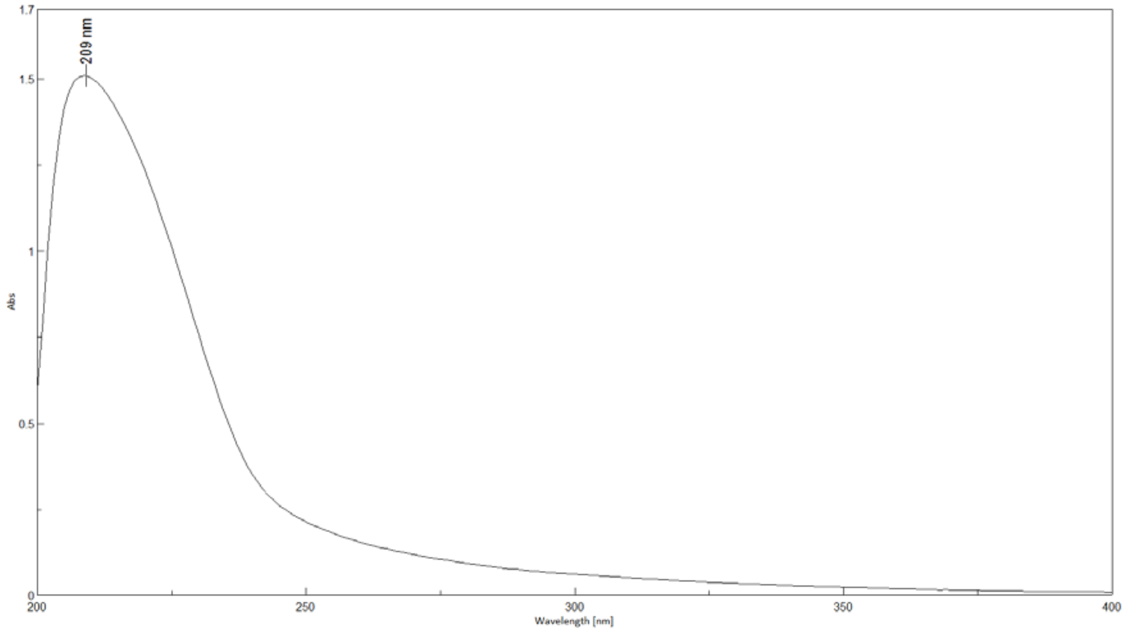

Figure S29. UV spectrum of **4**.

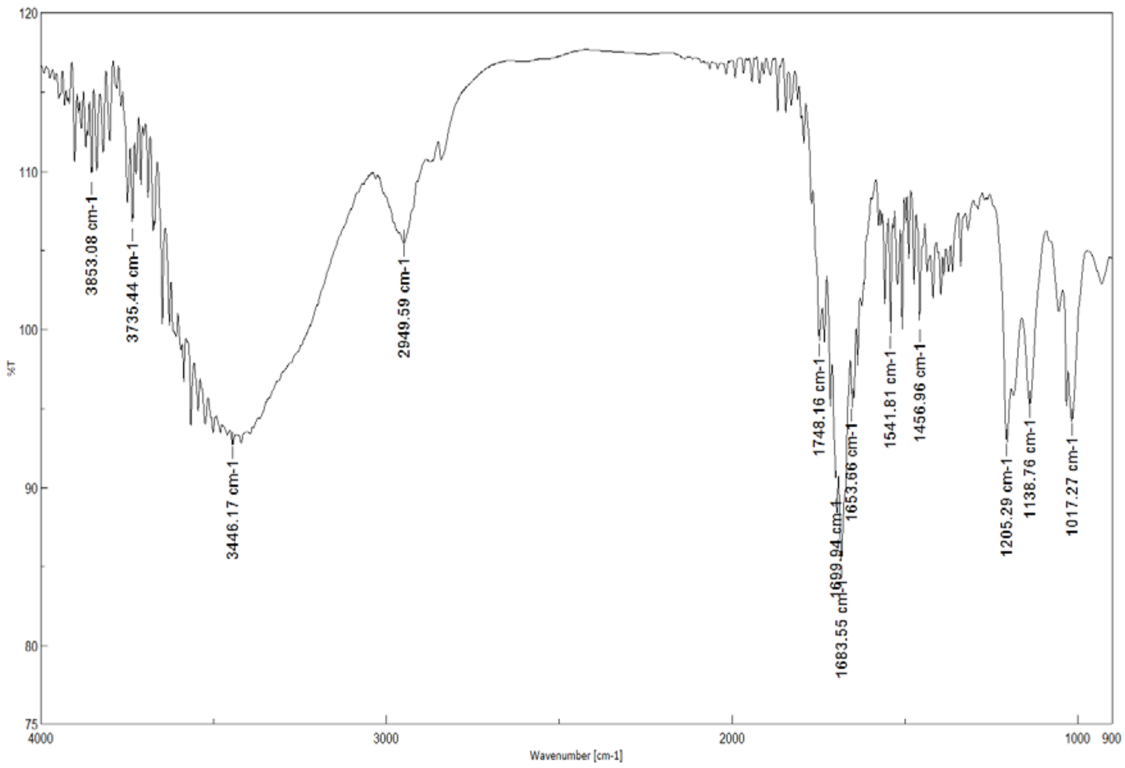

Figure S30. IR spectrum of **4**.

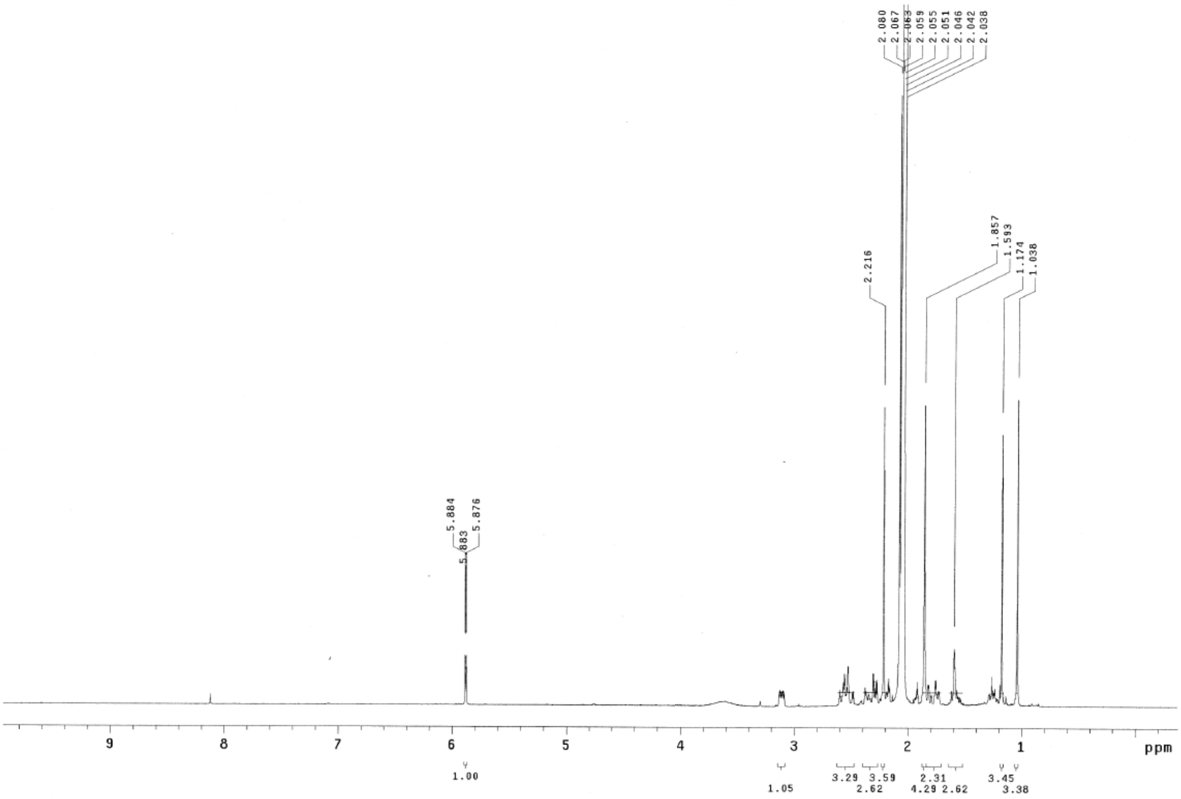

Figure S31. ^{1}H NMR spectrum of **4** in acetone-d_6.

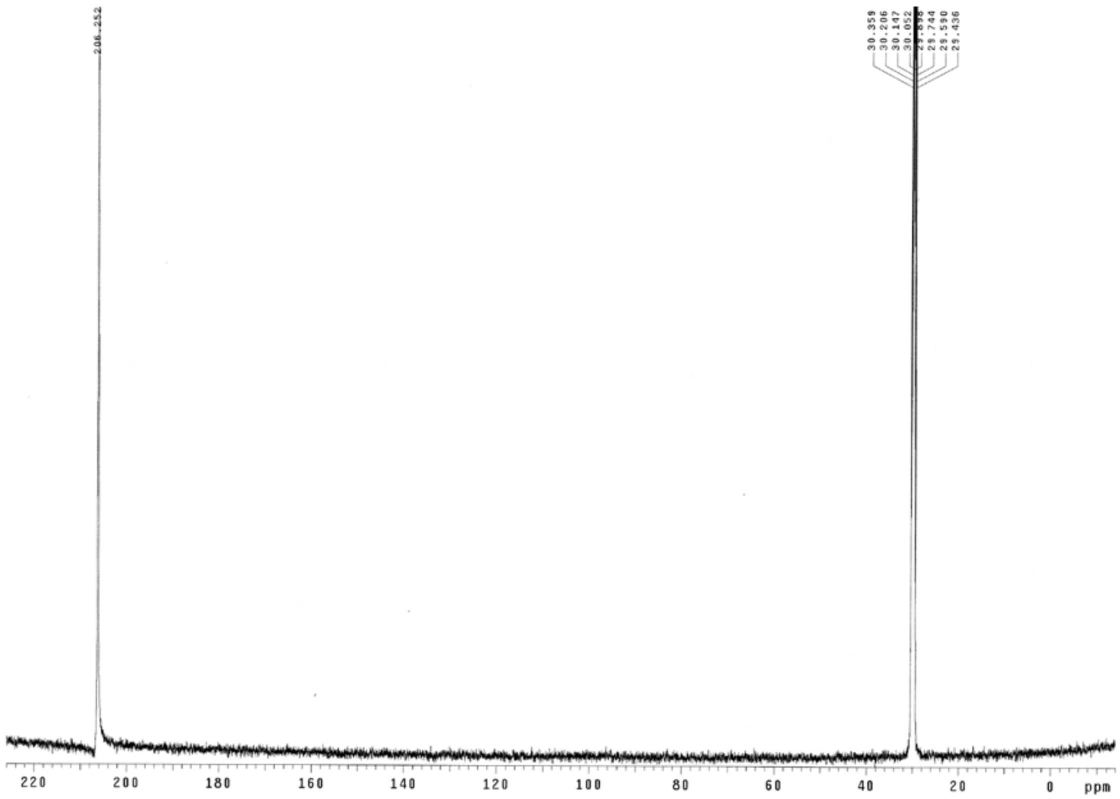

Figure S32. ^{13}C NMR spectrum of **4** in acetone-d_6.

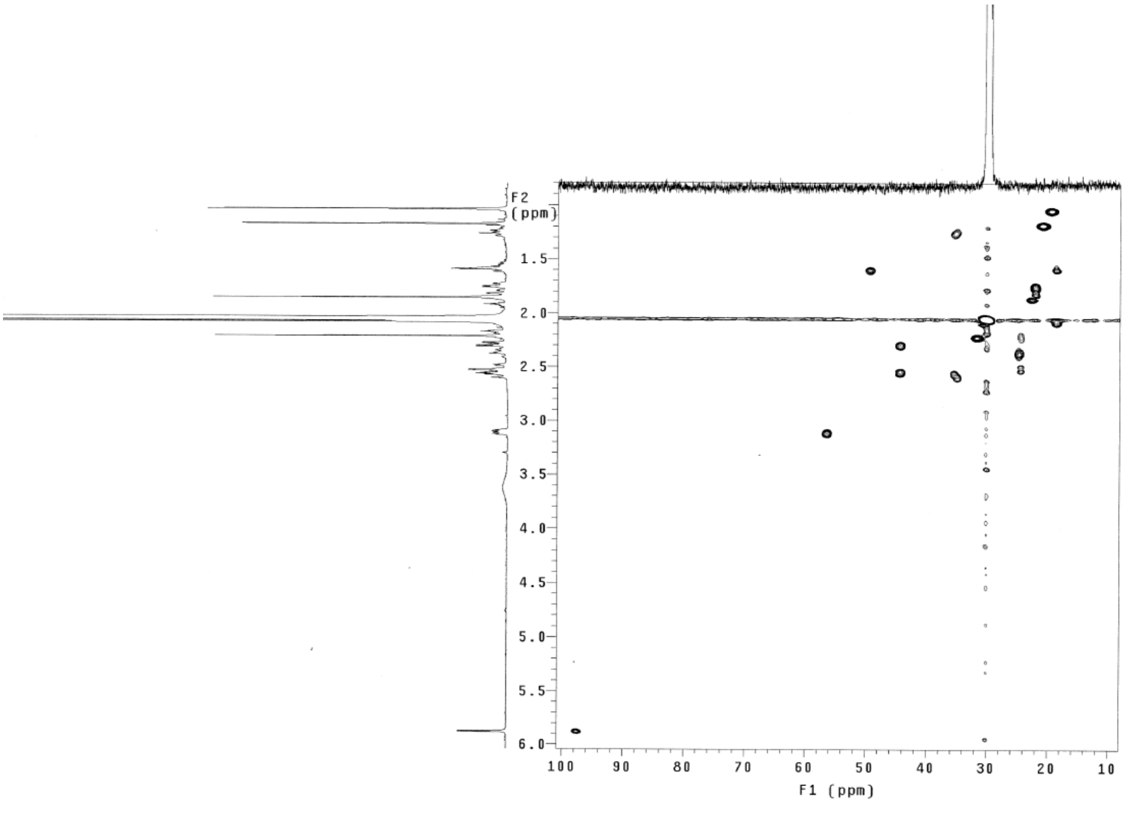

Figure S33. HSQC spectrum of **4** in acetone-d_6.

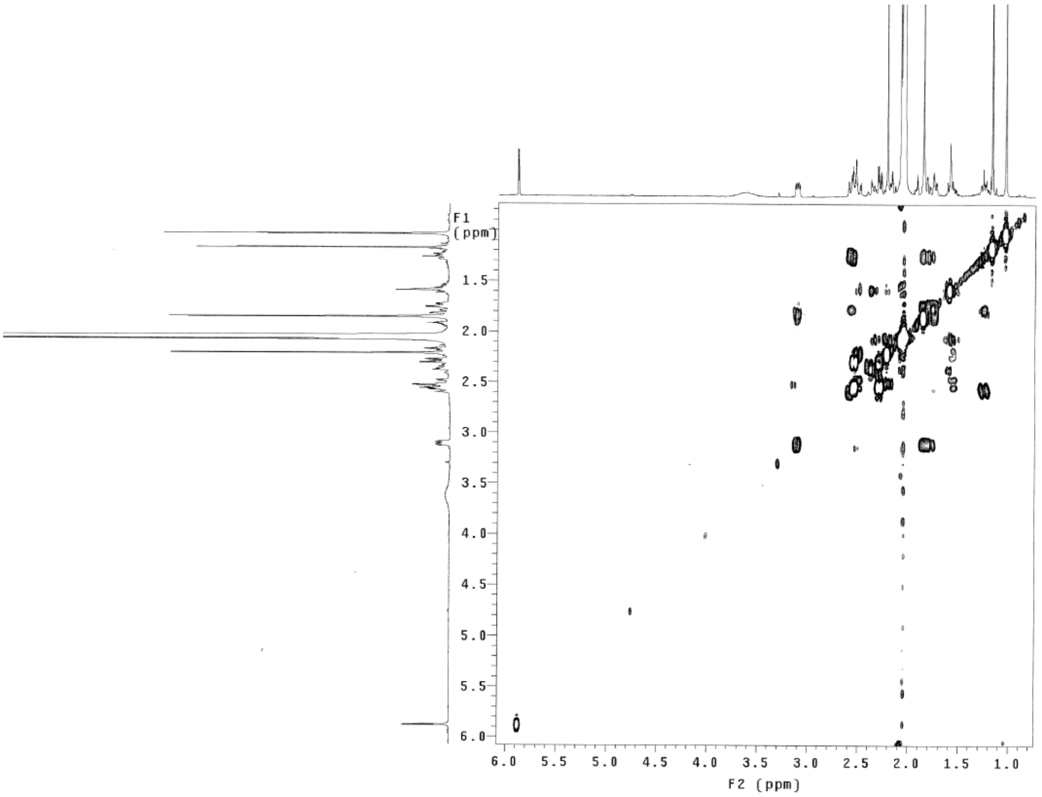

Figure S34. ^{1}H–^{1}H COSY spectrum of **4** in acetone-d_6.

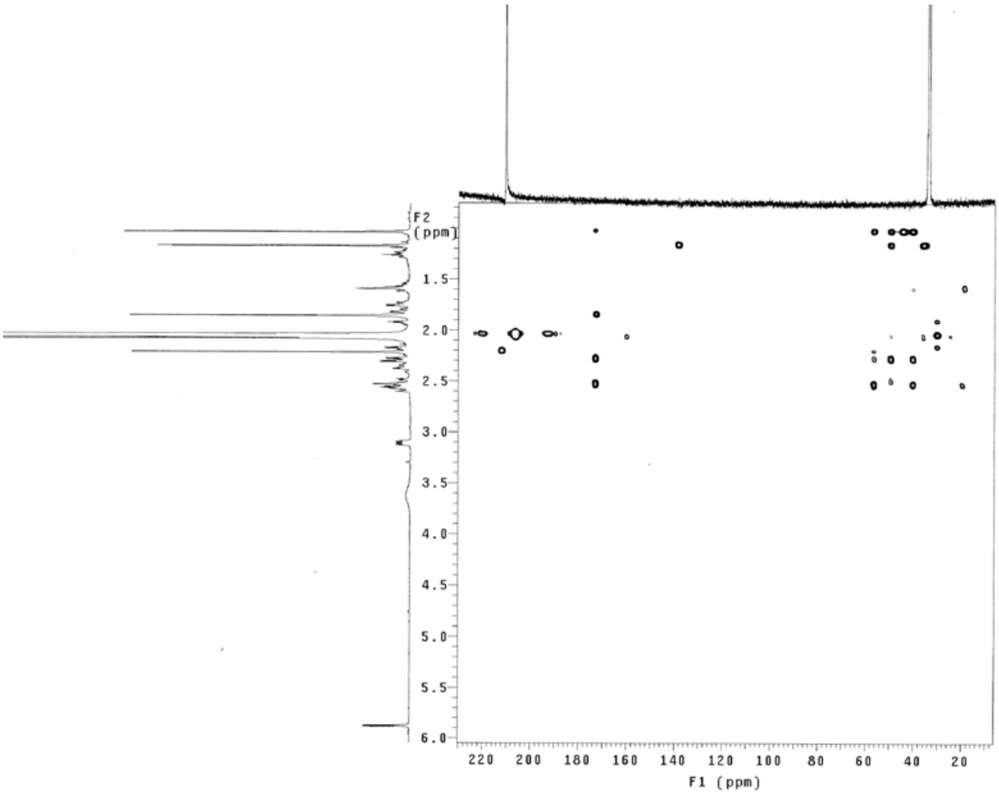

Figure S35. HMBC spectrum of **4** in acetone-d_6.

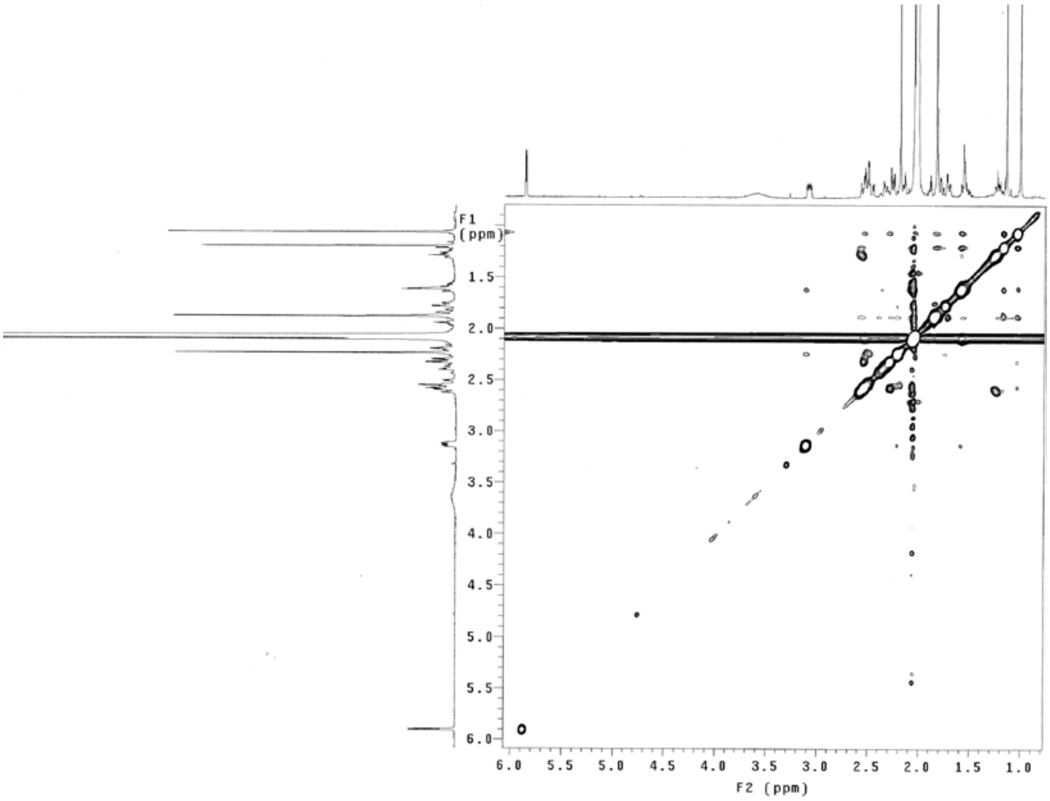

Figure S36. NOESY spectrum of **4** in acetone-d_6.

Table S6. The CD experimental data of **4**.

Wavelength [nm]	CD [mdeg]	Wavelength [nm]	CD [mdeg]	Wavelength [nm]	CD [mdeg]	Wavelength [nm]	CD [mdeg]
400.0	0.701685	375.2	0.089924	350.4	-0.21149	325.6	0.440831
399.6	0.699425	374.8	0.086017	350.0	-0.21961	325.2	0.442821
399.2	0.696615	374.4	0.082721	349.6	-0.22567	324.8	0.445519
398.8	0.693195	374.0	0.080013	349.2	-0.22954	324.4	0.448936
398.4	0.689125	373.6	0.077866	348.8	-0.23114	324.0	0.453081
398.0	0.684365	373.2	0.076252	348.4	-0.23039	323.6	0.457961
397.6	0.67887	372.8	0.075147	348.0	-0.22723	323.2	0.463586
397.2	0.672655	372.4	0.074526	347.6	-0.22165	322.8	0.469965
396.8	0.66585	372.0	0.074364	347.2	-0.21364	322.4	0.477111
396.4	0.65847	371.6	0.074638	346.8	-0.20324	322.0	0.485042
396.0	0.65055	371.2	0.075328	346.4	-0.1905	321.6	0.49378
395.6	0.642135	370.8	0.076414	346.0	-0.17551	321.2	0.50335
395.2	0.63327	370.4	0.077876	345.6	-0.15837	320.8	0.513775
394.8	0.624	370.0	0.079698	345.2	-0.13921	320.4	0.525095
394.4	0.61439	369.6	0.081863	344.8	-0.1182	320.0	0.53734
394.0	0.60448	369.2	0.084352	344.4	-0.09549	319.6	0.550545
393.6	0.594315	368.8	0.087147	344.0	-0.07128	319.2	0.564735
393.2	0.583935	368.4	0.090227	343.6	-0.04577	318.8	0.579945
392.8	0.573385	368.0	0.093566	343.2	-0.01918	318.4	0.59619
392.4	0.562695	367.6	0.097136	342.8	0.008285	318.0	0.61349
392.0	0.551895	367.2	0.100905	342.4	0.036388	317.6	0.63185
391.6	0.54101	366.8	0.104834	342.0	0.064906	317.2	0.65127
391.2	0.53005	366.4	0.108879	341.6	0.093614	316.8	0.671725
390.8	0.519035	366.0	0.112988	341.2	0.122291	316.4	0.69319
390.4	0.507975	365.6	0.117104	340.8	0.150725	316.0	0.71562
390.0	0.496874	365.2	0.121161	340.4	0.178711	315.6	0.738955
389.6	0.485727	364.8	0.125088	340.0	0.206056	315.2	0.763115
389.2	0.47453	364.4	0.128805	339.6	0.232579	314.8	0.788015
388.8	0.463273	364.0	0.132228	339.2	0.258116	314.4	0.81355
388.4	0.451944	363.6	0.135268	338.8	0.282515	314.0	0.839605
388.0	0.440527	363.2	0.13783	338.4	0.305644	313.6	0.866045
387.6	0.429003	362.8	0.13982	338.0	0.327387	313.2	0.89274
387.2	0.417354	362.4	0.141141	337.6	0.347646	312.8	0.919545
386.8	0.405562	362.0	0.141696	337.2	0.366343	312.4	0.946305
386.4	0.393608	361.6	0.141393	336.8	0.383418	312.0	0.972875
386.0	0.38148	361.2	0.140141	336.4	0.39883	311.6	0.999105
385.6	0.369165	360.8	0.137859	336.0	0.41256	311.2	1.02485
385.2	0.35666	360.4	0.134472	335.6	0.424606	310.8	1.04997
384.8	0.343965	360.0	0.129913	335.2	0.434987	310.4	1.074335
384.4	0.33109	359.6	0.124131	334.8	0.443739	310.0	1.097835
384.0	0.318052	359.2	0.117086	334.4	0.450917	309.6	1.12037
383.6	0.304878	358.8	0.108755	334.0	0.456594	309.2	1.14186
383.2	0.291603	358.4	0.099133	333.6	0.460856	308.8	1.162245
382.8	0.278269	358.0	0.088232	333.2	0.463806	308.4	1.181485
382.4	0.26493	357.6	0.076083	332.8	0.465559	308.0	1.19956
382.0	0.251643	357.2	0.062739	332.4	0.466239	307.6	1.21648
381.6	0.238471	356.8	0.048271	332.0	0.465981	307.2	1.23228
381.2	0.225482	356.4	0.032772	331.6	0.464924	306.8	1.24702
380.8	0.212743	356.0	0.016356	331.2	0.463211	306.4	1.260775
380.4	0.200323	355.6	-0.00084	330.8	0.460988	306.0	1.273655
380.0	0.188291	355.2	-0.01868	330.4	0.458398	305.6	1.285785
379.6	0.176707	354.8	-0.03698	330.0	0.455579	305.2	1.29731
379.2	0.165631	354.4	-0.05556	329.6	0.452667	304.8	1.3084
378.8	0.155113	354.0	-0.07423	329.2	0.449787	304.4	1.319235
378.4	0.145196	353.6	-0.09278	328.8	0.447056	304.0	1.330005
378.0	0.135915	353.2	-0.11101	328.4	0.444579	303.6	1.34092
377.6	0.127296	352.8	-0.12869	328.0	0.442452	303.2	1.352195
377.2	0.119357	352.4	-0.1456	327.6	0.440758	302.8	1.364035
376.8	0.112106	352.0	-0.16154	327.2	0.439566	302.4	1.376665
376.4	0.105545	351.6	-0.1763	326.8	0.438935	302.0	1.390295
376.0	0.09967	351.2	-0.18967	326.4	0.438912	301.6	1.40514
375.6	0.094469	350.8	-0.20146	326.0	0.439535	301.2	1.421395

Wavelength [nm]	CD [mdeg]	Wavelength [nm]	CD [mdeg]	Wavelength [nm]	CD [mdeg]	Wavelength [nm]	CD [mdeg]
300.8	1.439245	275.2	1.626725	249.6	-1.50158	224.0	16.0721
300.4	1.45887	274.8	1.543765	249.2	-1.62918	223.6	15.832
300.0	1.480415	274.4	1.459945	248.8	-1.75666	223.2	15.50875
299.6	1.50403	274.0	1.375435	248.4	-1.88252	222.8	15.1004
299.2	1.529815	273.6	1.2904	248.0	-2.00519	222.4	14.60555
298.8	1.55787	273.2	1.205025	247.6	-2.12303	222.0	14.02335
298.4	1.588255	272.8	1.119505	247.2	-2.23437	221.6	13.35345
298.0	1.62101	272.4	1.034035	246.8	-2.33753	221.2	12.59605
297.6	1.65614	272.0	0.94884	246.4	-2.43081	220.8	11.7521
297.2	1.69363	271.6	0.864145	246.0	-2.51253	220.4	10.82315
296.8	1.73343	271.2	0.780215	245.6	-2.58105	220.0	9.81145
296.4	1.775455	270.8	0.697325	245.2	-2.63476	219.6	8.7199
296.0	1.819595	270.4	0.615765	244.8	-2.67212	219.2	7.55225
295.6	1.86572	270.0	0.53585	244.4	-2.69166	218.8	6.3129
295.2	1.913645	269.6	0.457909	244.0	-2.692	218.4	5.0069
294.8	1.963185	269.2	0.382279	243.6	-2.67183	218.0	3.640145
294.4	2.014115	268.8	0.309302	243.2	-2.62994	217.6	2.21913
294.0	2.066175	268.4	0.239323	242.8	-2.56523	217.2	0.751
293.6	2.1191	268.0	0.17268	242.4	-2.47669	216.8	-0.75651
293.2	2.1726	267.6	0.1097	242.0	-2.36341	216.4	-2.2951
292.8	2.22636	267.2	0.050692	241.6	-2.22458	216.0	-3.85602
292.4	2.28006	266.8	-0.00406	241.2	-2.05949	215.6	-5.4301
292.0	2.33337	266.4	-0.05431	240.8	-1.86754	215.2	-7.00775
291.6	2.385945	266.0	-0.09984	240.4	-1.64822	214.8	-8.57925
291.2	2.437445	265.6	-0.14049	240.0	-1.40115	214.4	-10.1346
290.8	2.487525	265.2	-0.17614	239.6	-1.12602	214.0	-11.6637
290.4	2.535845	264.8	-0.20672	239.2	-0.82269	213.6	-13.1566
290.0	2.58208	264.4	-0.23223	238.8	-0.49107	213.2	-14.6031
289.6	2.62591	264.0	-0.25273	238.4	-0.13127	212.8	-15.9935
289.2	2.667025	263.6	-0.26833	238.0	0.2565	212.4	-17.318
288.8	2.70514	263.2	-0.27921	237.6	0.671885	212.0	-18.5675
288.4	2.739985	262.8	-0.28563	237.2	1.114355	211.6	-19.733
288.0	2.771315	262.4	-0.2879	236.8	1.583215	211.2	-20.806
287.6	2.798905	262.0	-0.28639	236.4	2.07759	210.8	-21.7788
287.2	2.822545	261.6	-0.28154	236.0	2.59641	210.4	-22.644
286.8	2.84206	261.2	-0.27385	235.6	3.13839	210.0	-23.3951
286.4	2.8573	260.8	-0.26386	235.2	3.702055	209.6	-24.0265
286.0	2.86813	260.4	-0.25219	234.8	4.28569	209.2	-24.5332
285.6	2.874455	260.0	-0.23947	234.4	4.887365	208.8	-24.9111
285.2	2.876195	259.6	-0.22639	234.0	5.5049	208.4	-25.1574
284.8	2.873295	259.2	-0.21366	233.6	6.13595	208.0	-25.27
284.4	2.865725	258.8	-0.20202	233.2	6.77785	207.6	-25.248
284.0	2.853485	258.4	-0.19222	232.8	7.4278	207.2	-25.0917
283.6	2.83659	258.0	-0.18502	232.4	8.0827	206.8	-24.8025
283.2	2.815075	257.6	-0.18118	232.0	8.7393	206.4	-24.3829
282.8	2.78901	257.2	-0.18143	231.6	9.39415	206.0	-23.8368
282.4	2.75847	256.8	-0.18649	231.2	10.0436	205.6	-23.1691
282.0	2.723565	256.4	-0.19705	230.8	10.68385	205.2	-22.3859
281.6	2.684415	256.0	-0.21374	230.4	11.31095	204.8	-21.4945
281.2	2.641155	255.6	-0.23715	230.0	11.92075	204.4	-20.5079
280.8	2.59395	255.2	-0.26778	229.6	12.50905	204.0	-19.4446
280.4	2.542955	254.8	-0.30607	229.2	13.0716	203.6	-18.3234
280.0	2.48837	254.4	-0.35238	228.8	13.60405	203.2	-17.1634
279.6	2.430375	254.0	-0.40695	228.4	14.10195	202.8	-15.9839
279.2	2.36918	253.6	-0.46993	228.0	14.56085	202.4	-14.8039
278.8	2.30499	253.2	-0.54133	227.6	14.9764	202.0	-13.6858
278.4	2.23801	252.8	-0.62105	227.2	15.3442	201.6	-12.6541
278.0	2.168465	252.4	-0.70887	226.8	15.6598	201.2	-11.7164
277.6	2.09655	252.0	-0.80442	226.4	15.9191	200.8	-10.8792
277.2	2.022485	251.6	-0.90718	226.0	16.11785	200.4	-10.1482
276.8	1.94647	251.2	-1.0165	225.6	16.25215	200.0	-9.5282
276.4	1.8687	250.8	-1.1316	225.2	16.31815		
276.0	1.789365	250.4	-1.25154	224.8	16.31235		
275.6	1.708645	250.0	-1.37527	224.4	16.2313		

Table S7. The cartesian coordinates of conformer **4a**.

C	-1.557402	0.106309	0.115678	H	-1.530468	-1.141517	-1.636435
C	-1.801670	-1.283023	-0.581010	H	-1.099808	-3.334028	-0.594681
C	-0.897493	-2.411617	-0.035435	H	-1.166441	-2.624518	1.004881
C	0.588311	-2.066614	-0.166217	H	1.201425	-2.881773	0.229412
C	0.942061	-0.742565	0.554369	H	0.839321	-1.972358	-1.231897
C	-0.012843	0.386228	0.005643	H	0.176666	0.375126	-1.079975
C	2.349683	-0.299262	0.201713	H	1.715097	2.479488	-1.144337
C	2.727924	0.956718	-0.074383	H	2.273834	2.980485	0.438079
C	1.820250	2.139544	-0.103261	H	-0.275096	2.549010	0.207595
C	0.444395	1.779327	0.491353	H	0.508873	1.801135	1.585374
C	3.559056	-1.158233	0.163074	H	4.766507	1.578603	0.384298
O	4.635865	-0.381985	-0.185365	H	-1.677404	1.514446	-1.582244
C	4.206370	0.992879	-0.354690	H	-3.185095	0.670023	-1.246397
C	-2.330612	1.163900	-0.777896	H	-4.469594	3.016456	0.746422
C	-2.896157	2.391492	-0.088444	H	-3.273604	-2.836260	-2.432605
O	-4.174568	2.195345	0.306233	H	-4.892150	-2.523553	-1.735288
C	-3.248511	-1.792215	-0.549341	H	-3.948253	-1.215421	-2.511755
C	-3.891109	-2.113432	-1.884163	H	-3.182787	0.166060	1.576506
O	-3.836045	-1.997870	0.502063	H	-1.762870	-0.679450	2.162217
O	-2.328316	3.452788	0.088886	H	-1.747739	1.082340	2.056402
C	-2.092314	0.167447	1.561507	H	1.008229	-0.026328	2.642881
C	0.934148	-0.967624	2.090655	H	0.041731	-1.493869	2.433662
O	4.442480	1.442909	-1.651016	H	1.797365	-1.583330	2.365384
O	3.689188	-2.341353	0.399939	H	5.388885	1.645003	-1.738342

Table S8. The cartesian coordinates of conformer **4c**.

C	1.415025	-0.414315	0.296779	H	1.687059	0.850318	-1.426815
C	1.849994	0.951968	-0.344737	H	1.311505	3.057703	-0.394030
C	0.974996	2.146828	0.115484	H	1.131090	2.324367	1.185518
C	-0.505682	1.924155	-0.197795	H	-0.631111	1.847405	-1.286674
C	-1.047646	0.637401	0.467897	H	-1.094878	2.786651	0.127094
C	-0.133982	-0.570238	0.026442	H	-0.210481	-0.562450	-1.072419
C	-2.436391	0.312150	-0.051026	H	-1.875527	-2.539539	-1.296204
C	-2.882404	-0.908860	-0.377492	H	-2.679950	-2.946438	0.207971
C	-2.085514	-2.165587	-0.282643	H	-0.104679	-2.757168	0.306675
C	-0.769871	-1.908170	0.478731	H	-0.973006	-1.892236	1.554919
C	-3.562585	1.265868	-0.206904	H	-4.447768	-1.058429	-1.890791
O	-4.651963	0.580498	-0.681397	H	1.763134	-2.537898	-0.114464
C	-4.317068	-0.823437	-0.827572	H	1.783302	-1.521497	-1.554209
C	2.102938	-1.576419	-0.509416	H	3.606524	-2.613842	1.102919
C	3.621593	-1.597552	-0.536509	H	4.864295	2.594825	-1.038021
O	4.250891	-2.236827	0.479906	H	4.159461	1.350107	-2.107223
C	3.302523	1.405646	-0.156347	H	3.256525	2.835048	-1.791217
C	3.937797	2.103466	-1.343167	H	1.322332	-1.473585	2.199930
O	3.901002	1.285614	0.902464	H	2.850960	-0.606870	1.938554
O	4.294417	-1.100912	-1.416262	H	1.415999	0.276882	2.390607
C	1.771917	-0.561213	1.791131	H	-2.026972	1.566568	2.167454
C	-1.199693	0.869872	1.995436	H	-1.429536	-0.054075	2.533512
O	-5.124978	-1.621249	-0.022695	H	-0.309203	1.312303	2.444974
O	-3.618002	2.457329	0.016508	H	-5.995691	-1.703157	-0.446014

Table S9. ^{13}C and ^{1}H NMR data for compound **5** (125/500 MHz) in acetone-d_6.

	**5**	
position	δ_H	δ_C
1	1.45, dd (7.0, 3.0) [a]	33.8, CH_2
2α	1.99, dd (7.0, 3.0)	26.4, CH_2
2β	1.52, dd (7.0, 3.0)	
3	3.35, d (3.0)	75.7, CH
4	–	38.3, C
5	1.50, m	49.9, CH
6	1.55, m	18.4, CH_2
7α	1.57, m	37.6, CH_2
7β	2.04, m	
8	–	38.4, CH
9	1.21, m	56.6, CH
10	–	38.4, C
11α	1.87, dd (13.5, 7.0)	17.7, CH_2
11β	1.56, m	
12α	2.05, m	22.2, CH_2
12β	2.28, dd (18.0, 6.0)	
13	–	127.6, C
14	–	168.7, C
15	6.16, br s	98.0, CH
16	–	171.7, C
17	1.26, s	21.0, CH_3
18	0.94, s	29.1, CH_3
19	0.86, s	22.4, CH_3
20	0.95, s	16.9, CH_3
3-OH	3.40, d (4.0)	

a. *J* values (Hz) in parentheses.

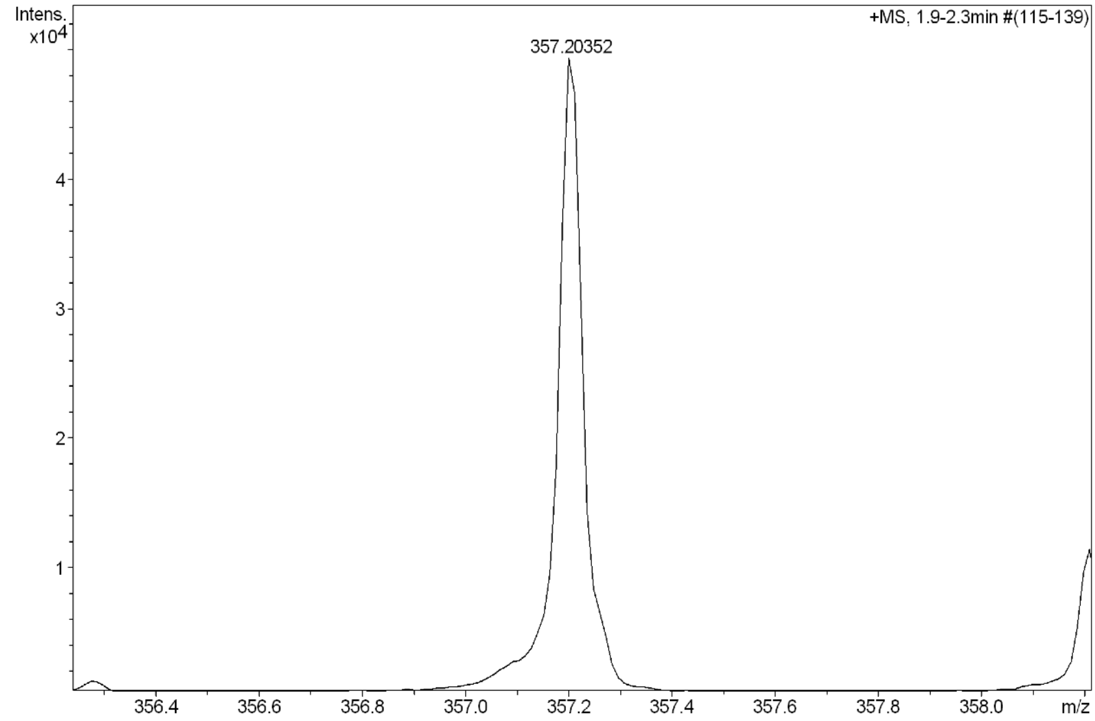

Meas. m/z	#	Formula	Score	m/z	err [mDa]	err [ppm]	mSigma	rdb	e⁻ Conf	N-Rule
357.20352	1	C 20 H 30 Na O 4	100.00	357.20363	0.11	0.31	18.2	5.5	even	ok

Figure S37. HRESIMS spectrum of **5**.

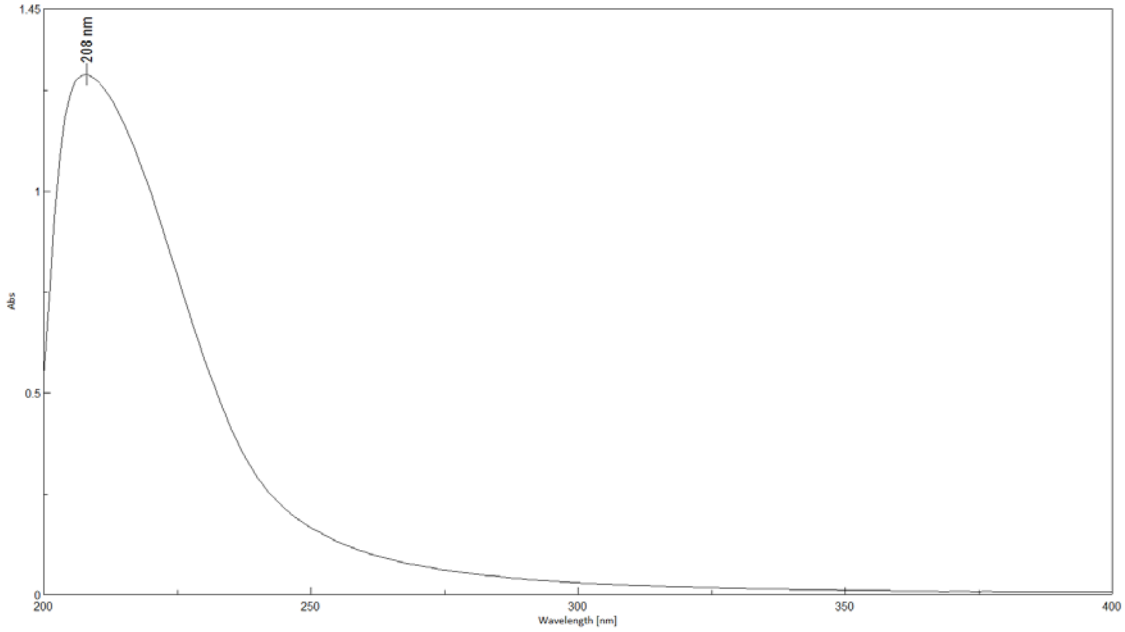

Figure S38. UV spectrum of **5**.

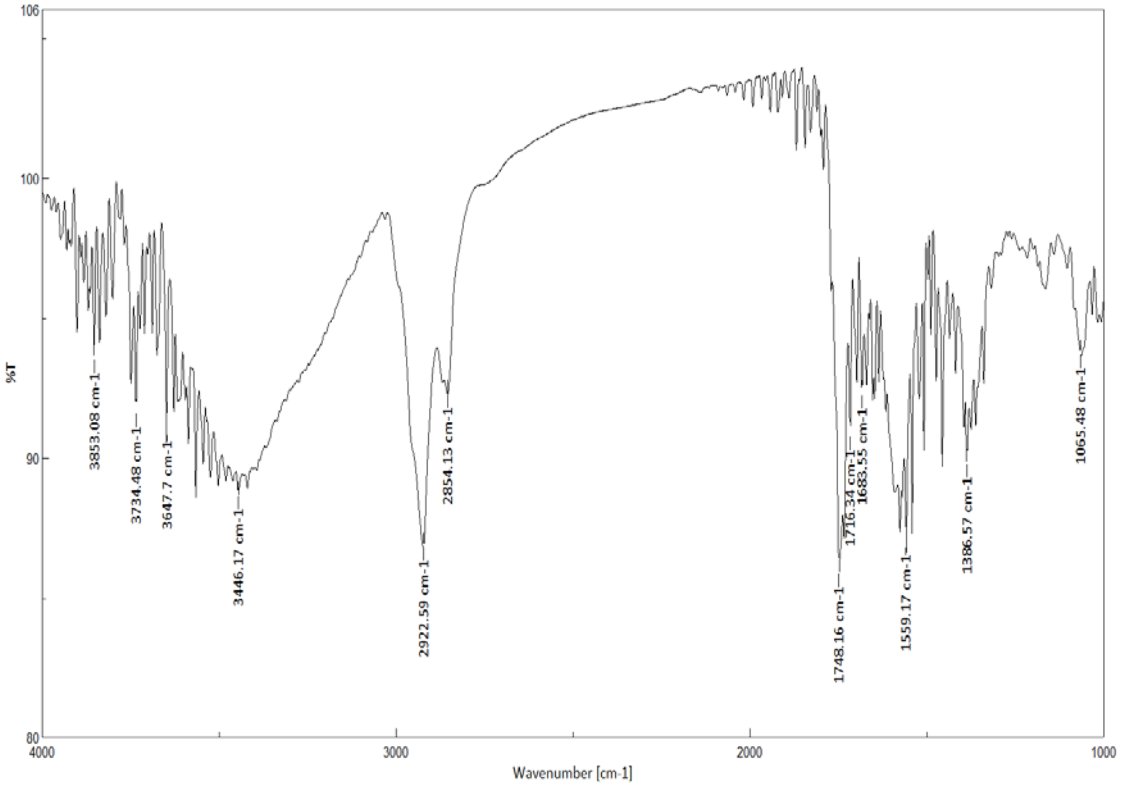

Figure S39. IR spectrum of **5**.

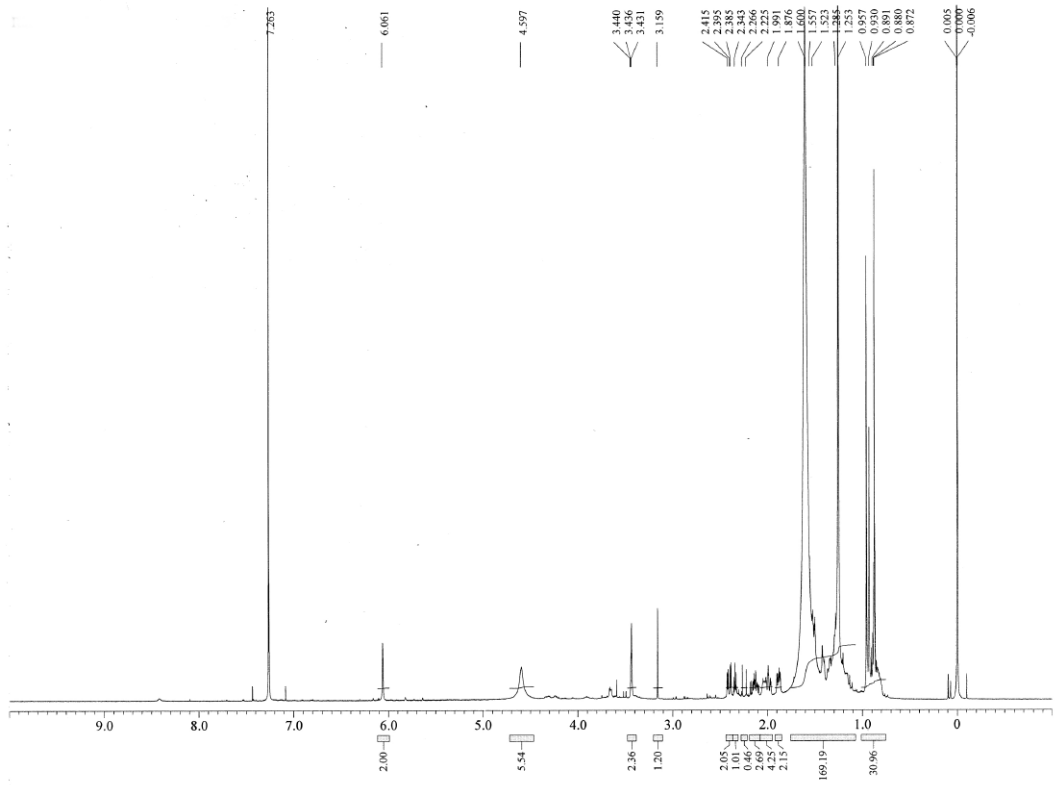

Figure S40. ^{1}H NMR spectrum of **5** in $CDCl_3$.

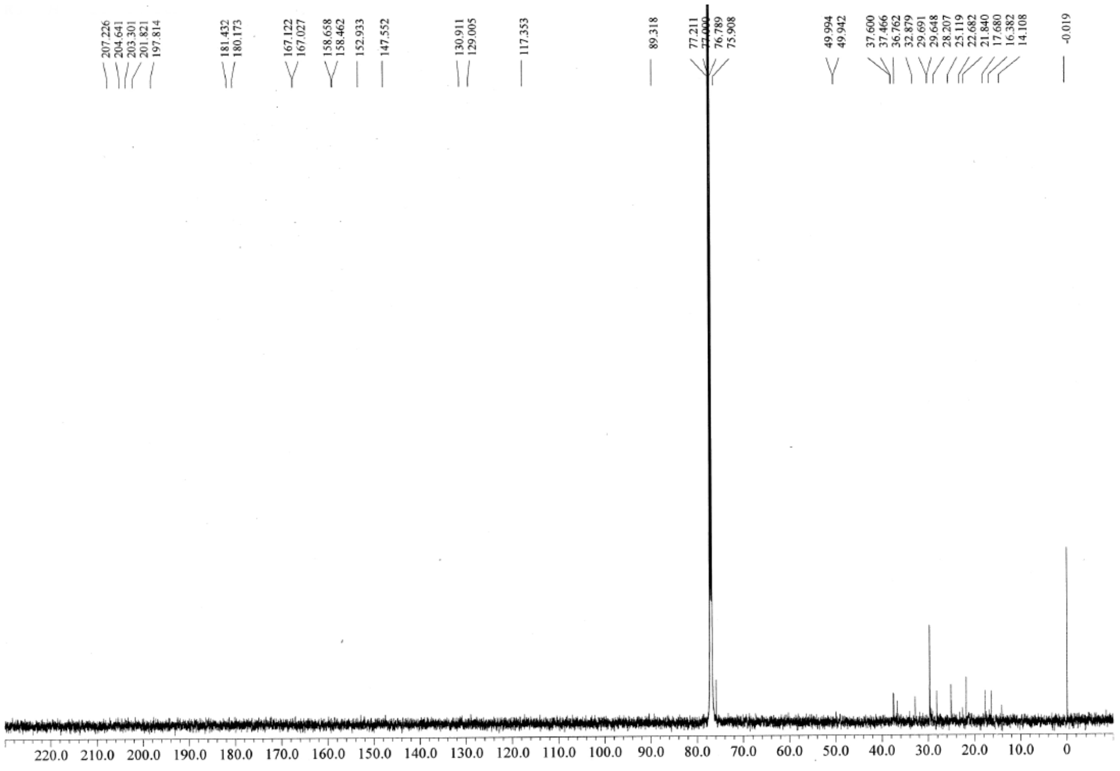

Figure S41. ^{13}C NMR spectrum of **5** in $CDCl_3$.

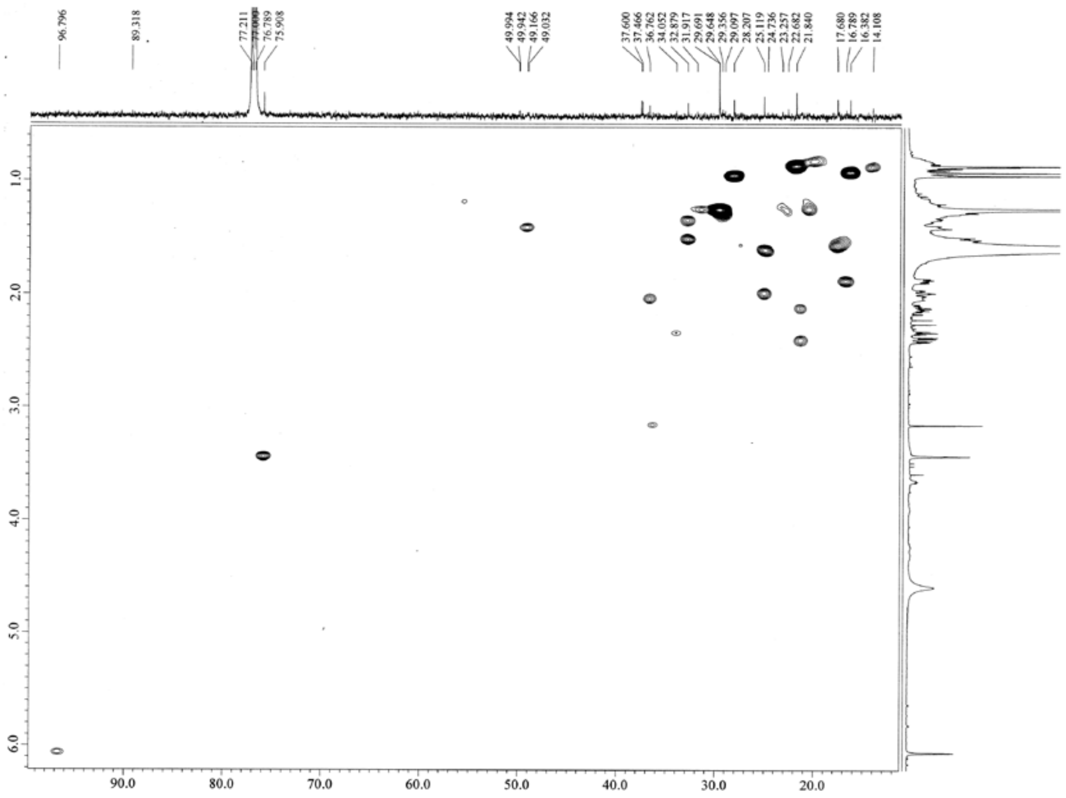

Figure S42. HSQC spectrum of **5** in $CDCl_3$.

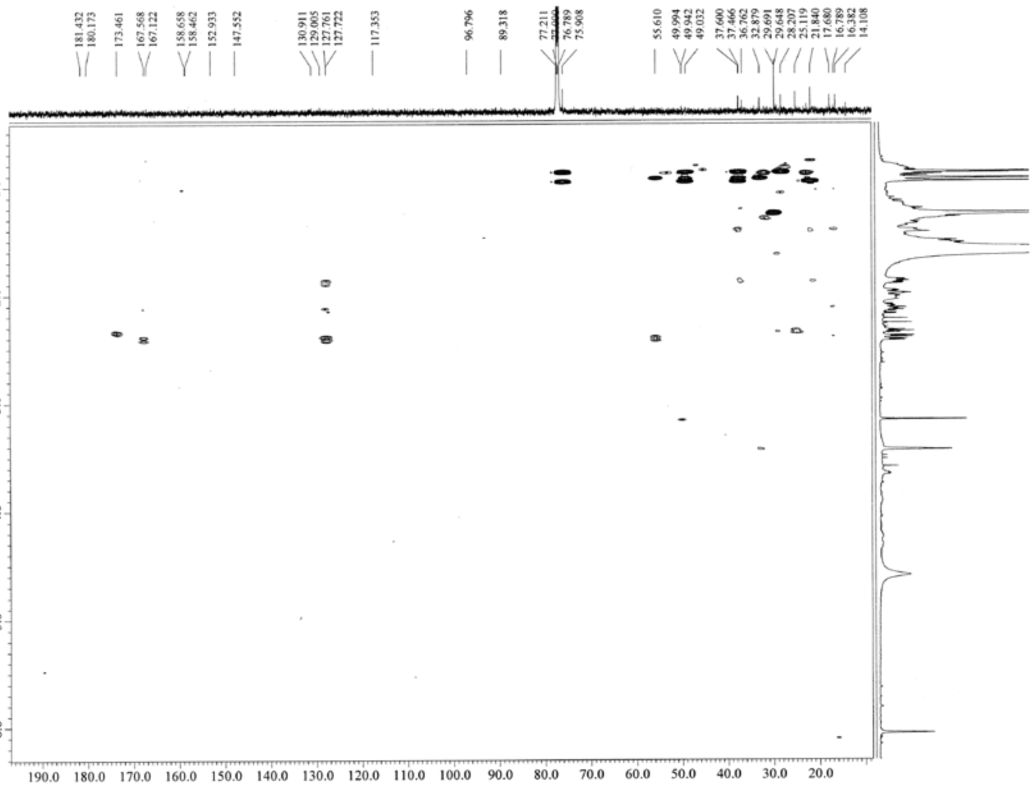

Figure S43. HMBC spectrum of **5** in $CDCl_3$.

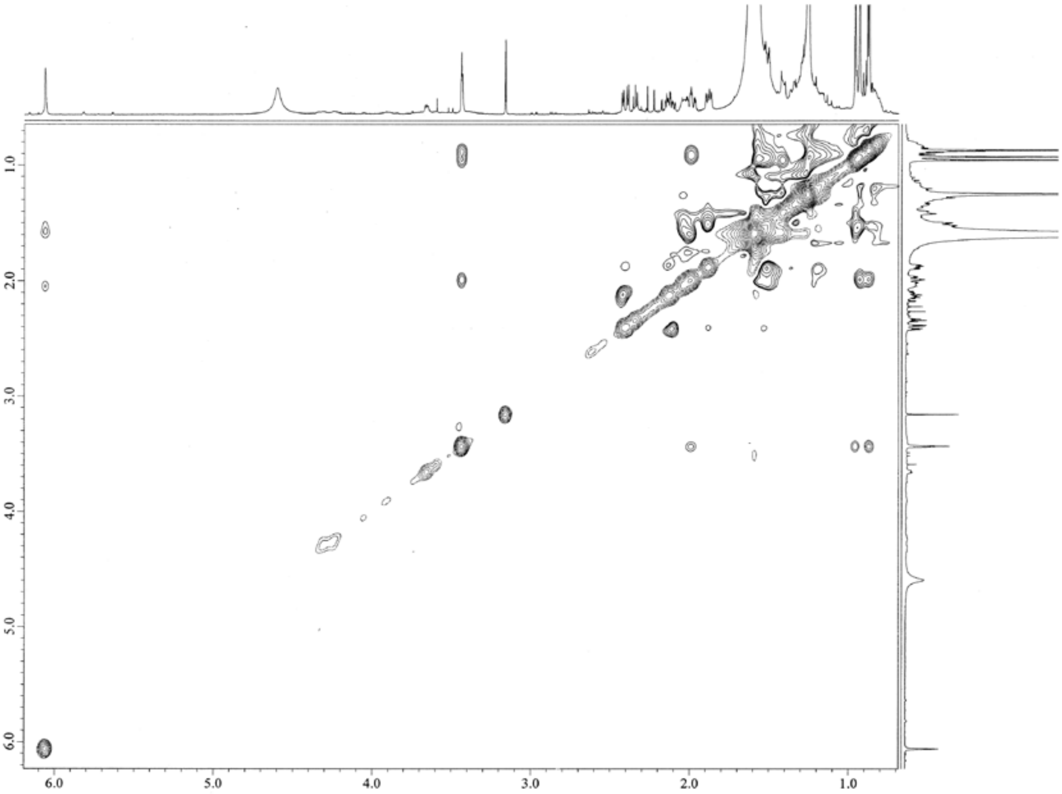

Figure S44. NOESY spectrum of **5** in $CDCl_3$.

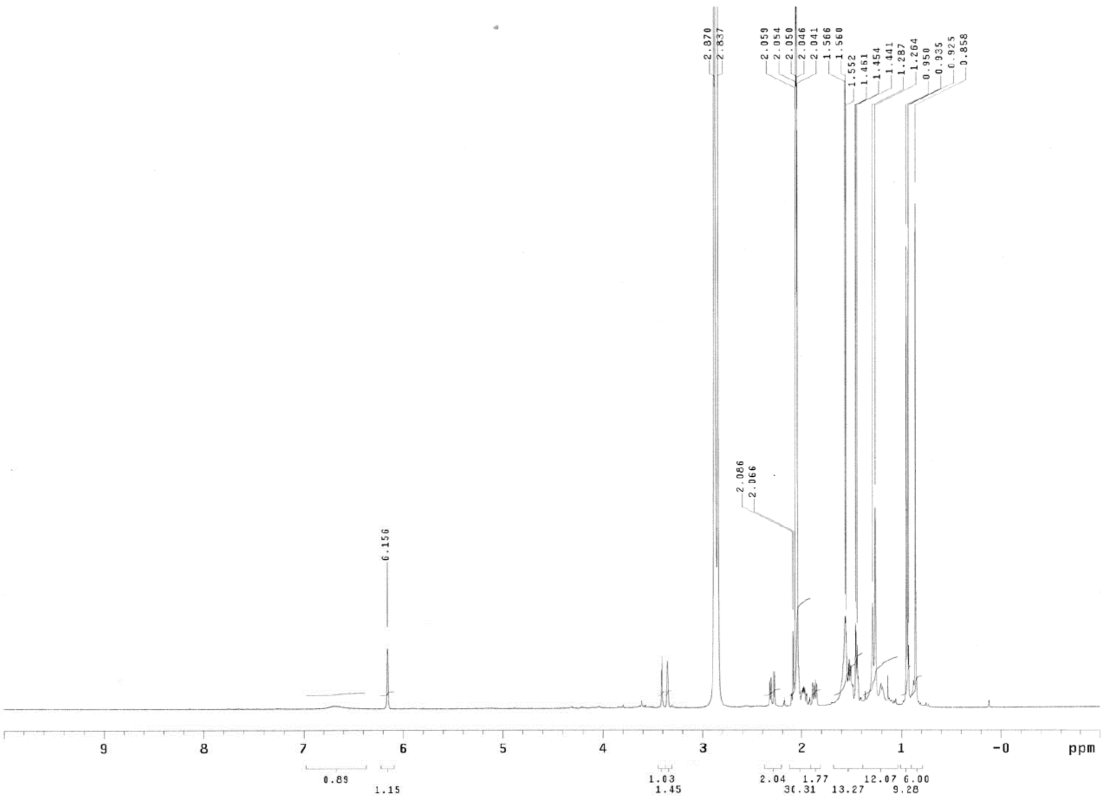

Figure S45. ^{1}H NMR spectrum of **5** in acetone-d_6.

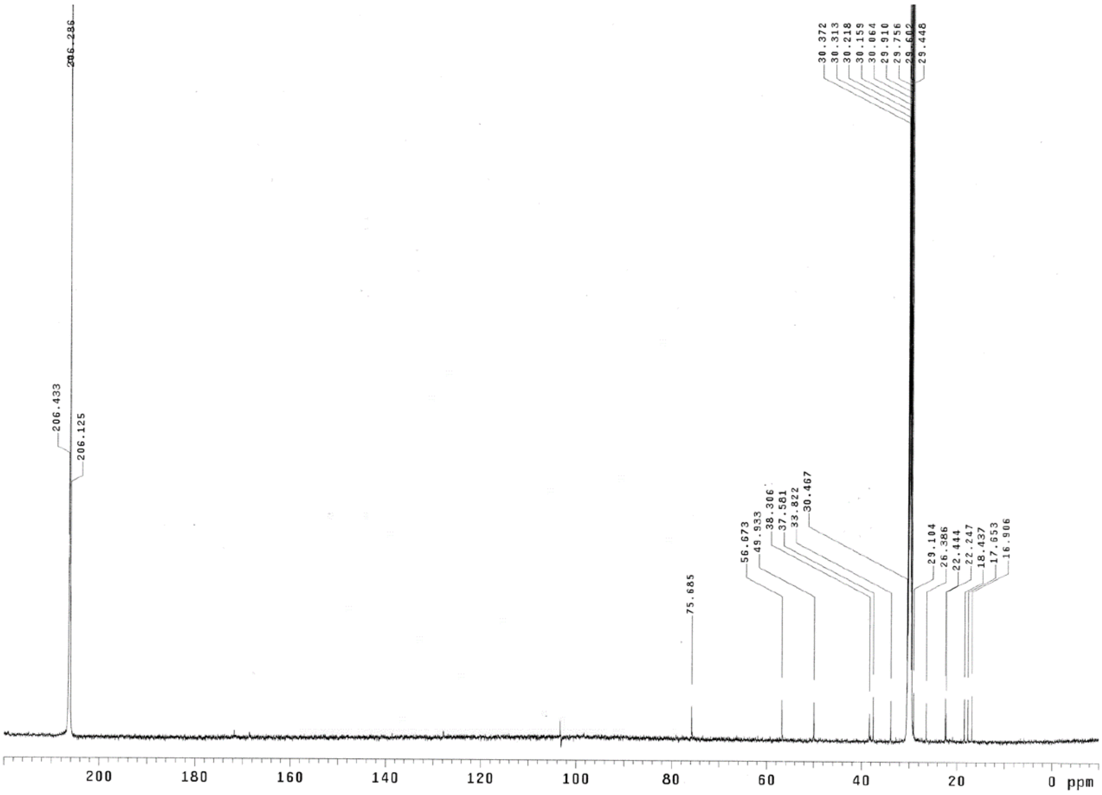

Figure S46. ^{13}C NMR spectrum of **5** in acetone-d_6.

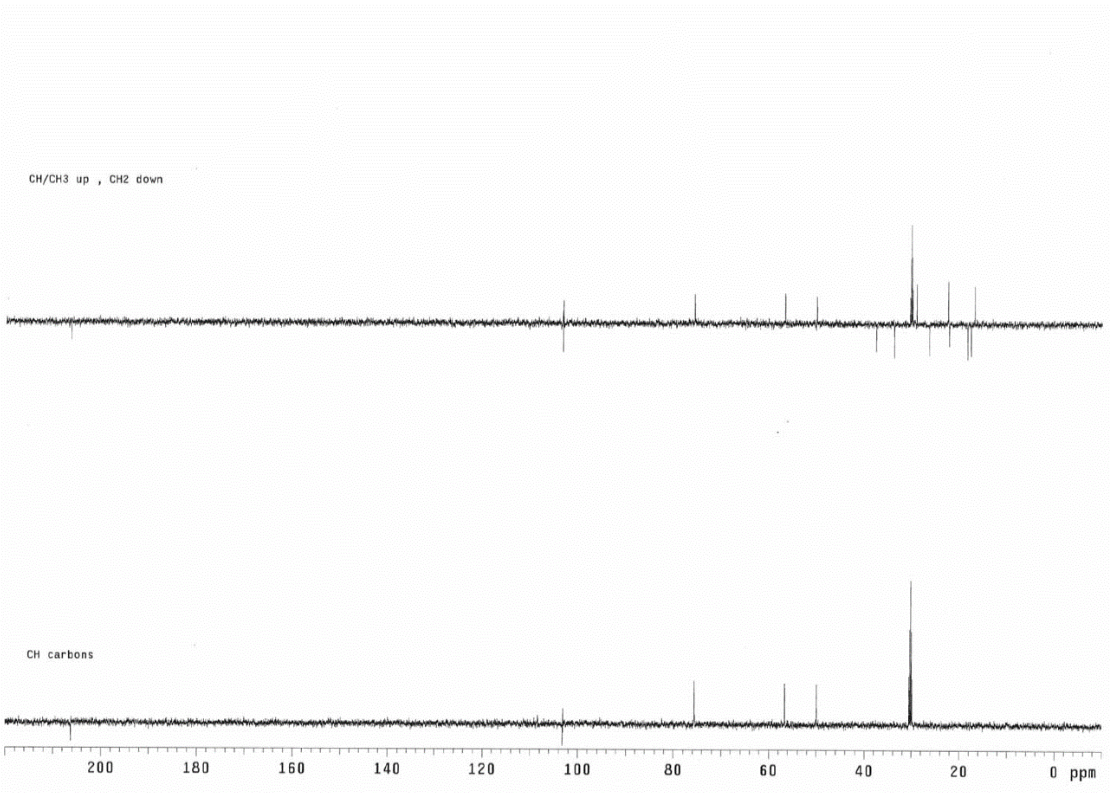

Figure S47. DEPT spectrum of **5** in acetone-d_6.

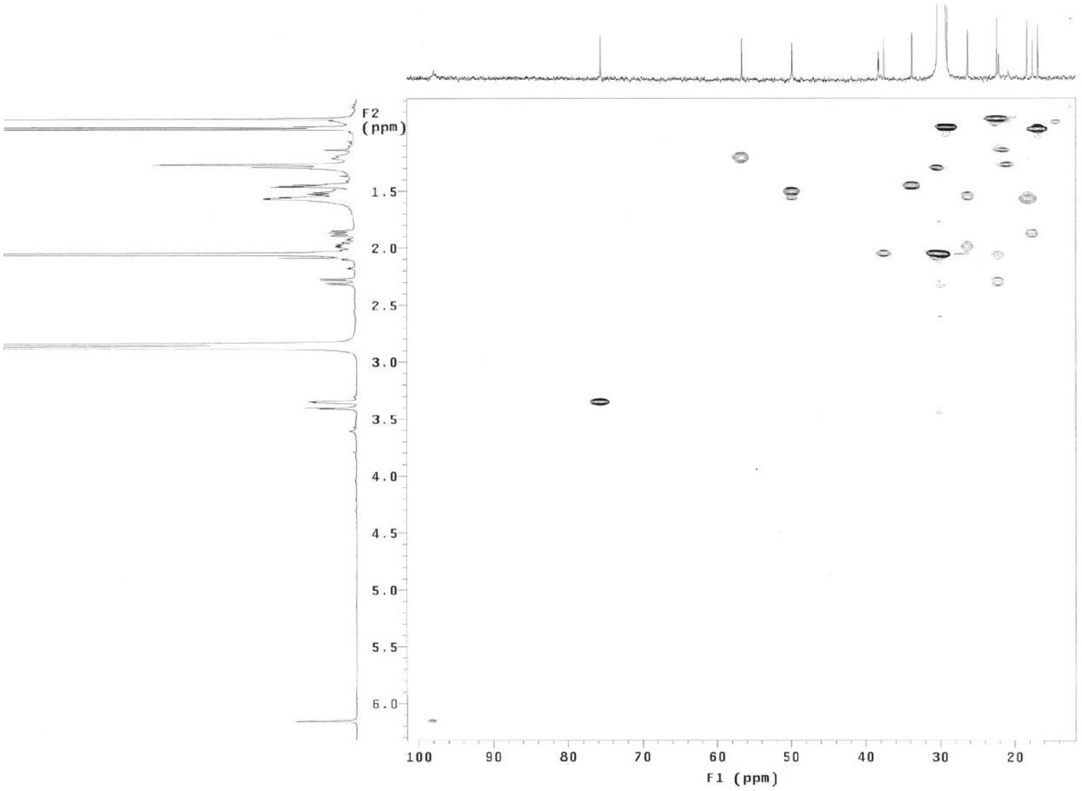

Figure S48. HSQC spectrum of **5** in acetone-d_6.

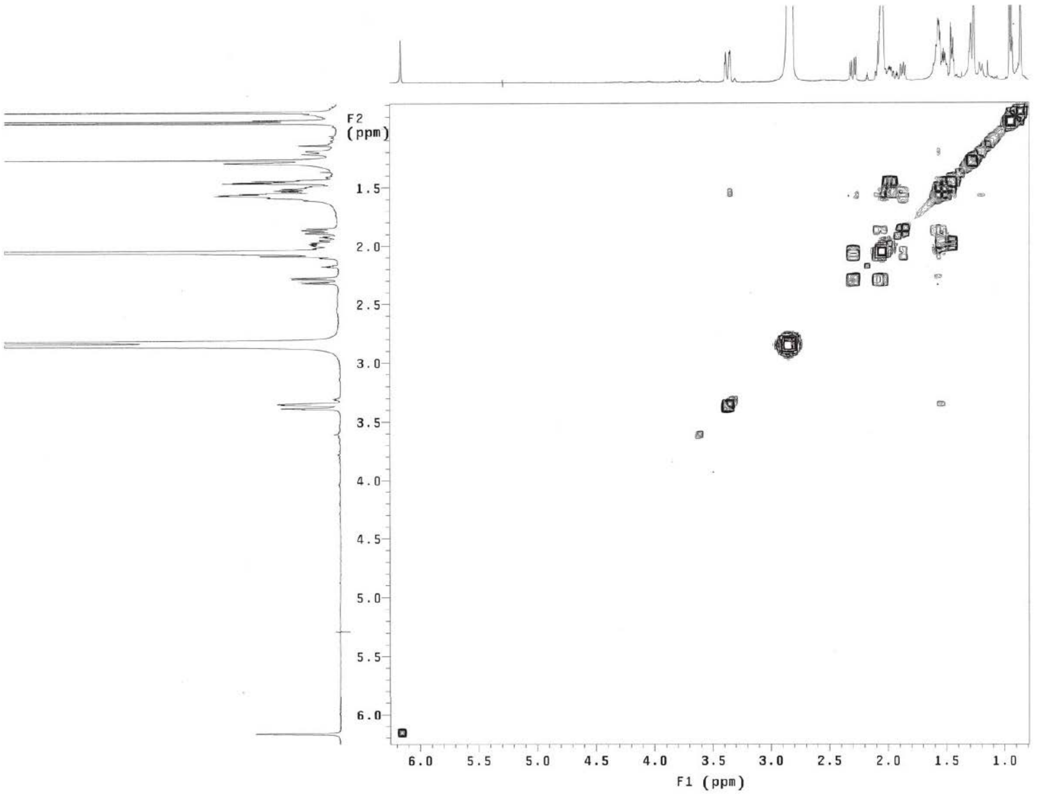

Figure S49. $^{1}H-^{1}H$ COSY spectrum of **5** in acetone-d_6.

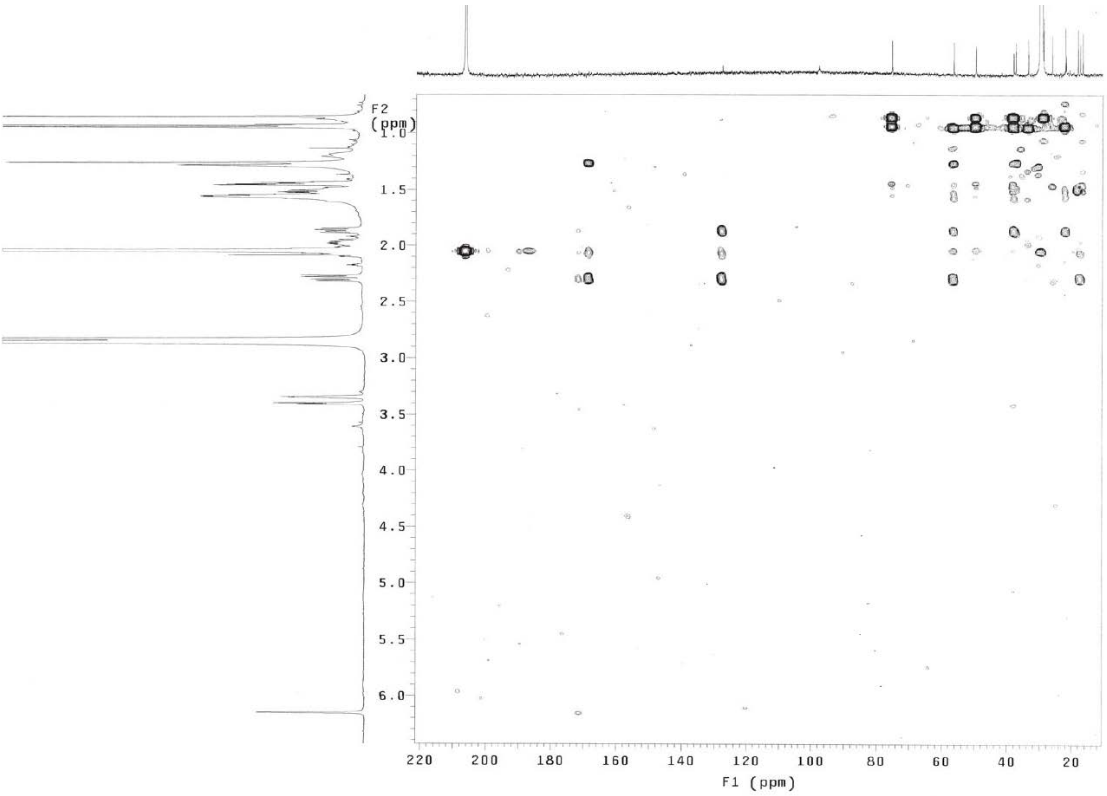

Figure S50. HMBC spectrum of **5** in acetone-d_6.

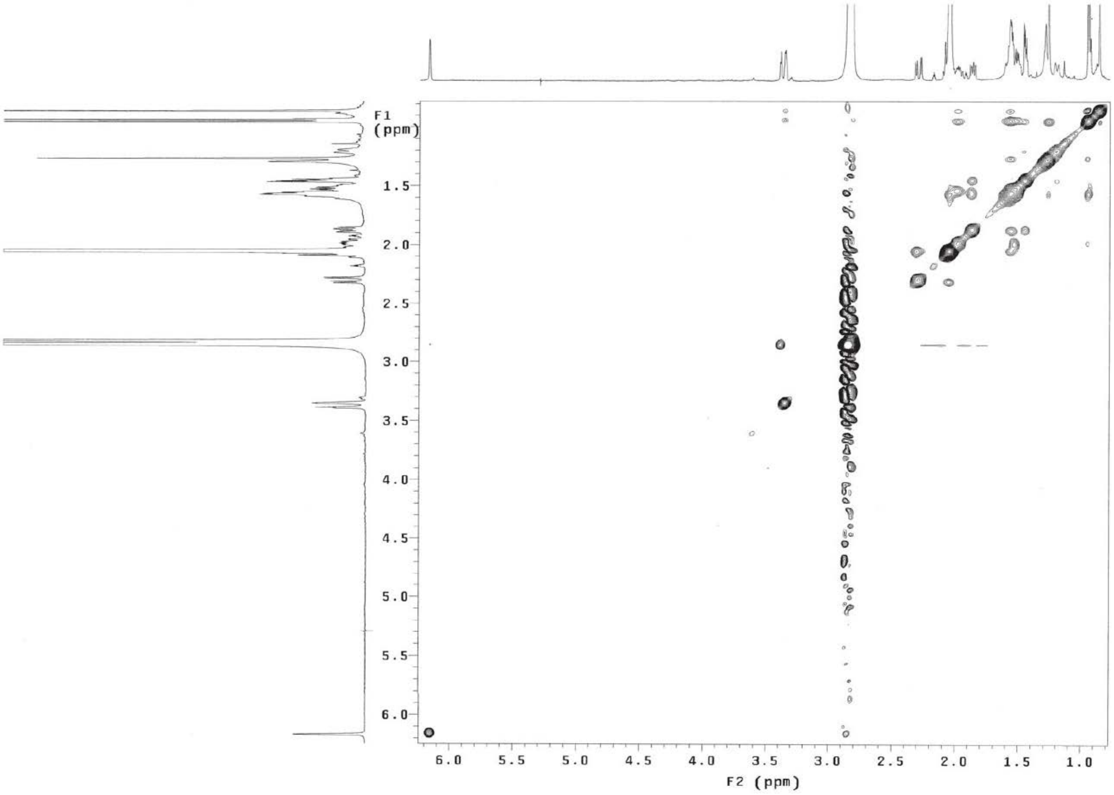

Figure S51. NOESY spectrum of **5** in acetone-d_6.

Table S10. The CD experimental data of **5**.

Wavelength [nm]	CD [mdeg]	Wavelength [nm]	CD [mdeg]	Wavelength [nm]	CD [mdeg]	Wavelength [nm]	CD [mdeg]
400.0	-0.38646	375.2	0.336575	350.4	1.19033	325.6	0.568042
399.6	-0.37066	374.8	0.312317	350.0	1.19194	325.2	0.538038
399.2	-0.35491	374.4	0.318723	349.6	1.18051	324.8	0.510011
398.8	-0.34848	374.0	0.341874	349.2	1.17167	324.4	0.502002
398.4	-0.34558	373.6	0.370816	348.8	1.16196	324.0	0.504736
398.0	-0.34395	373.2	0.396845	348.4	1.16017	323.6	0.506536
397.6	-0.34602	372.8	0.418305	348.0	1.16414	323.2	0.503908
397.2	-0.35318	372.4	0.434449	347.6	1.16695	322.8	0.503704
396.8	-0.35737	372.0	0.45676	347.2	1.15771	322.4	0.501303
396.4	-0.36437	371.6	0.488191	346.8	1.12527	322.0	0.496941
396.0	-0.36882	371.2	0.518408	346.4	1.08291	321.6	0.489104
395.6	-0.36273	370.8	0.560746	346.0	1.04502	321.2	0.462455
395.2	-0.35694	370.4	0.609768	345.6	1.00778	320.8	0.427017
394.8	-0.3454	370.0	0.648991	345.2	0.985406	320.4	0.385927
394.4	-0.33531	369.6	0.6647	344.8	0.973056	320.0	0.342524
394.0	-0.31567	369.2	0.652976	344.4	0.960725	319.6	0.313313
393.6	-0.27998	368.8	0.626342	344.0	0.94112	319.2	0.290793
393.2	-0.23329	368.4	0.610006	343.6	0.931862	318.8	0.268881
392.8	-0.17595	368.0	0.615477	343.2	0.918967	318.4	0.251233
392.4	-0.10707	367.6	0.629861	342.8	0.908743	318.0	0.231072
392.0	-0.04622	367.2	0.643534	342.4	0.897142	317.6	0.198862
391.6	0.00377	366.8	0.658119	342.0	0.878161	317.2	0.169854
391.2	0.043943	366.4	0.668197	341.6	0.862541	316.8	0.141443
390.8	0.072995	366.0	0.677924	341.2	0.858093	316.4	0.113573
390.4	0.087158	365.6	0.694473	340.8	0.863934	316.0	0.108712
390.0	0.092571	365.2	0.716025	340.4	0.872147	315.6	0.103996
389.6	0.087468	364.8	0.738566	340.0	0.888446	315.2	0.098178
389.2	0.073641	364.4	0.757048	339.6	0.91297	314.8	0.091973
388.8	0.060268	364.0	0.770397	339.2	0.93991	314.4	0.067243
388.4	0.054231	363.6	0.777862	338.8	0.959766	314.0	0.052186
388.0	0.065031	363.2	0.78238	338.4	0.968796	313.6	0.049755
387.6	0.083114	362.8	0.79239	338.0	0.947867	313.2	0.041623
387.2	0.107433	362.4	0.807232	337.6	0.905035	312.8	0.031553
386.8	0.151021	362.0	0.817982	337.2	0.865609	312.4	0.023094
386.4	0.201649	361.6	0.819027	336.8	0.850932	312.0	0.006783
386.0	0.247511	361.2	0.812948	336.4	0.852853	311.6	-0.00611
385.6	0.293694	360.8	0.803005	336.0	0.874086	311.2	-0.01212
385.2	0.335448	360.4	0.793723	335.6	0.901947	310.8	-0.02557
384.8	0.380455	360.0	0.790269	335.2	0.92154	310.4	-0.03879
384.4	0.442253	359.6	0.813739	334.8	0.908817	310.0	-0.05004
384.0	0.520751	359.2	0.86272	334.4	0.878487	309.6	-0.05918
383.6	0.586814	358.8	0.927203	334.0	0.841205	309.2	-0.07062
383.2	0.629578	358.4	0.985911	333.6	0.790066	308.8	-0.10336
382.8	0.644609	358.0	1.01803	333.2	0.751217	308.4	-0.15322
382.4	0.639317	357.6	1.01798	332.8	0.733063	308.0	-0.2067
382.0	0.632149	357.2	1.00863	332.4	0.714621	307.6	-0.26023
381.6	0.609968	356.8	1.00624	332.0	0.691143	307.2	-0.31189
381.2	0.596291	356.4	1.01289	331.6	0.659605	306.8	-0.34698
380.8	0.58678	356.0	1.02994	331.2	0.628993	306.4	-0.37768
380.4	0.57808	355.6	1.03957	330.8	0.599078	306.0	-0.41208
380.0	0.593613	355.2	1.0419	330.4	0.573697	305.6	-0.42579
379.6	0.594923	354.8	1.04282	330.0	0.559474	305.2	-0.43682
379.2	0.570919	354.4	1.04211	329.6	0.542629	304.8	-0.46008
378.8	0.533924	354.0	1.03535	329.2	0.522076	304.4	-0.49233
378.4	0.491024	353.6	1.02434	328.8	0.511251	304.0	-0.52948
378.0	0.446954	353.2	1.01936	328.4	0.513722	303.6	-0.55281
377.6	0.415802	352.8	1.03137	328.0	0.520894	303.2	-0.56272
377.2	0.400087	352.4	1.06219	327.6	0.543192	302.8	-0.55773
376.8	0.388213	352.0	1.1033	327.2	0.565616	302.4	-0.56035
376.4	0.385814	351.6	1.14332	326.8	0.587395	302.0	-0.57433
376.0	0.378556	351.2	1.17023	326.4	0.598079	301.6	-0.59194
375.6	0.363968	350.8	1.17884	326.0	0.591418	301.2	-0.62071

Wavelength [nm]	CD [mdeg]	Wavelength [nm]	CD [mdeg]	Wavelength [nm]	CD [mdeg]	Wavelength [nm]	CD [mdeg]
300.8	-0.64937	275.2	0.089804	249.6	-7.39512	224.0	12.7626
300.4	-0.67171	274.8	0.106174	249.2	-7.50902	223.6	12.4566
300.0	-0.68162	274.4	0.123363	248.8	-7.57667	223.2	12.0459
299.6	-0.66527	274.0	0.131878	248.4	-7.6356	222.8	11.5735
299.2	-0.6339	273.6	0.150347	248.0	-7.68369	222.4	11.0979
298.8	-0.6162	273.2	0.184322	247.6	-7.7231	222.0	10.5932
298.4	-0.60867	272.8	0.227554	247.2	-7.75743	221.6	10.0754
298.0	-0.61634	272.4	0.275682	246.8	-7.74691	221.2	9.52282
297.6	-0.63101	272.0	0.314327	246.4	-7.69945	220.8	8.94226
297.2	-0.64001	271.6	0.338996	246.0	-7.60871	220.4	8.35951
296.8	-0.63928	271.2	0.348419	245.6	-7.48943	220.0	7.71277
296.4	-0.62846	270.8	0.341983	245.2	-7.35833	219.6	6.93892
296.0	-0.60299	270.4	0.325778	244.8	-7.21788	219.2	6.17605
295.6	-0.58148	270.0	0.290684	244.4	-7.08185	218.8	5.43729
295.2	-0.57328	269.6	0.238024	244.0	-6.917	218.4	4.70589
294.8	-0.57993	269.2	0.193511	243.6	-6.72376	218.0	4.02903
294.4	-0.59621	268.8	0.153312	243.2	-6.48507	217.6	3.35908
294.0	-0.60893	268.4	0.1153	242.8	-6.19181	217.2	2.66628
293.6	-0.60243	268.0	0.094569	242.4	-5.84293	216.8	1.93661
293.2	-0.58483	267.6	0.075611	242.0	-5.46985	216.4	1.18769
292.8	-0.56408	267.2	0.049985	241.6	-5.07571	216.0	0.41711
292.4	-0.54486	266.8	0.023893	241.2	-4.68643	215.6	-0.37192
292.0	-0.5314	266.4	-0.01747	240.8	-4.28335	215.2	-1.16376
291.6	-0.53335	266.0	-0.06733	240.4	-3.8707	214.8	-1.88311
291.2	-0.53803	265.6	-0.12724	240.0	-3.45193	214.4	-2.64181
290.8	-0.54003	265.2	-0.18826	239.6	-2.97653	214.0	-3.5209
290.4	-0.53573	264.8	-0.24869	239.2	-2.44537	213.6	-4.39837
290.0	-0.50983	264.4	-0.32738	238.8	-1.8761	213.2	-5.27134
289.6	-0.47803	264.0	-0.44425	238.4	-1.24159	212.8	-6.18682
289.2	-0.44725	263.6	-0.59335	238.0	-0.59711	212.4	-6.99648
288.8	-0.42072	263.2	-0.75557	237.6	0.008358	212.0	-7.74706
288.4	-0.40583	262.8	-0.93808	237.2	0.629272	211.6	-8.49765
288.0	-0.39902	262.4	-1.11823	236.8	1.2285	211.2	-9.30593
287.6	-0.38998	262.0	-1.27847	236.4	1.84538	210.8	-10.1407
287.2	-0.37407	261.6	-1.43423	236.0	2.50419	210.4	-10.958
286.8	-0.35859	261.2	-1.57516	235.6	3.17191	210.0	-11.751
286.4	-0.34856	260.8	-1.70673	235.2	3.87545	209.6	-12.4808
286.0	-0.3413	260.4	-1.86432	234.8	4.56927	209.2	-13.0961
285.6	-0.34512	260.0	-2.03529	234.4	5.20492	208.8	-13.5869
285.2	-0.34763	259.6	-2.23242	234.0	5.80375	208.4	-14.0392
284.8	-0.35555	259.2	-2.42958	233.6	6.31522	208.0	-14.3887
284.4	-0.36227	258.8	-2.60458	233.2	6.78379	207.6	-14.6983
284.0	-0.362	258.4	-2.79	232.8	7.29363	207.2	-15.0553
283.6	-0.35883	258.0	-2.97782	232.4	7.82787	206.8	-15.3901
283.2	-0.36108	257.6	-3.173	232.0	8.42654	206.4	-15.7146
282.8	-0.34833	257.2	-3.39043	231.6	9.06371	206.0	-15.9212
282.4	-0.33785	256.8	-3.63279	231.2	9.69787	205.6	-15.9523
282.0	-0.3175	256.4	-3.88642	230.8	10.3103	205.2	-15.7729
281.6	-0.27533	256.0	-4.16912	230.4	10.8599	204.8	-15.1738
281.2	-0.23557	255.6	-4.44114	230.0	11.3292	204.4	-14.3359
280.8	-0.20026	255.2	-4.68854	229.6	11.7395	204.0	-13.3763
280.4	-0.16951	254.8	-4.92016	229.2	12.1014	203.6	-12.6602
280.0	-0.14113	254.4	-5.11795	228.8	12.4466	203.2	-11.3937
279.6	-0.09792	254.0	-5.31789	228.4	12.827	202.8	-9.5677
279.2	-0.0619	253.6	-5.53299	228.0	13.1986	202.4	-7.54289
278.8	-0.02644	253.2	-5.75272	227.6	13.505	202.0	-5.4443
278.4	0.011993	252.8	-5.9827	227.2	13.7359	201.6	-3.26039
278.0	0.032576	252.4	-6.21566	226.8	13.8552	201.2	-0.97293
277.6	0.048791	252.0	-6.44156	226.4	13.8474	200.8	1.25531
277.2	0.054487	251.6	-6.65166	226.0	13.7167	200.4	3.37126
276.8	0.053976	251.2	-6.82973	225.6	13.5317	200.0	5.156
276.4	0.047197	250.8	-6.99097	225.2	13.3259		
276.0	0.052538	250.4	-7.14096	224.8	13.1378		
275.6	0.069221	250.0	-7.27307	224.4	12.9904		

Table S11. The cartesian coordinates of conformer **5a**.

C	-3.527589	1.828258	0.141222	H	-1.574286	-0.257544	-1.267653
C	-4.023315	0.683879	-0.739813	H	-1.727634	2.748053	0.867722
C	-3.418342	-0.697093	-0.339061	H	-1.622551	2.198541	-0.801368
C	-1.859835	-0.555869	-0.246906	H	-1.517631	-2.673905	-0.613326
C	-1.304712	0.609976	0.654929	H	-1.257808	-2.211851	1.053507
C	-1.995358	1.924881	0.194521	H	0.490738	-1.536170	-1.369897
C	-1.113620	-1.876710	0.019144	H	0.896419	-2.694415	-0.102653
C	0.383805	-1.742961	-0.295229	H	0.254332	0.893957	-0.762359
C	1.065166	-0.608710	0.510543	H	2.971492	2.812761	0.825216
C	0.231937	0.716045	0.325057	H	2.112217	2.777807	-0.703051
C	2.443100	-0.314116	-0.047743	H	1.189692	1.734553	2.011857
C	2.975490	0.913778	-0.165228	H	0.364493	2.821213	0.912775
C	2.302859	2.196154	0.211053	H	3.066019	-1.821306	-1.504118
C	0.977944	1.917413	0.952405	H	-3.627249	-2.753078	-1.096071
C	3.426678	-1.325678	-0.596490	H	-4.851010	-1.641505	-1.697757
O	4.572011	-0.528149	-1.012389	H	-3.197163	-1.592019	-2.353751
C	4.314575	0.795231	-0.771614	H	-3.659888	-2.123266	1.306006
C	-3.786392	-1.725528	-1.438705	H	-5.167253	-1.379211	0.764170
C	-4.104411	-1.184213	0.957747	H	-4.048652	-0.466952	1.778445
C	-1.560377	0.439893	2.177169	H	-0.814230	0.992731	2.757835
O	5.117680	1.663709	-1.058932	H	-2.535837	0.838209	2.468360
O	-3.696843	1.057871	-2.096395	H	-1.530740	-0.599746	2.511108
O	3.811466	-2.258783	0.366783	H	-4.104278	0.419174	-2.702002
C	1.269494	-1.072140	1.980907	H	4.283070	-2.989372	-0.068032
H	-3.941781	1.704061	1.148272	H	1.652576	-0.266568	2.614071
H	-3.941552	2.768788	-0.243443	H	0.342278	-1.439660	2.422551
H	-5.119297	0.611891	-0.658738	H	1.998419	-1.887302	2.006340

Table S12. The cartesian coordinates of conformer **5c**.

C	3.490410	-1.855613	-0.261818	H	1.539818	0.520105	-1.164910
C	3.999357	-0.553738	-0.887700	H	1.551079	-2.005721	-1.226814
C	3.418171	0.717900	-0.196010	H	1.686097	-2.884674	0.281415
C	1.857405	0.579902	-0.109281	H	1.542607	2.730143	-0.011619
C	1.291638	-0.742203	0.531442	H	1.295348	1.926055	1.521342
C	1.956253	-1.940428	-0.204997	H	-0.495028	1.808694	-0.967211
C	1.132809	1.821761	0.441625	H	-0.860649	2.677630	0.514304
C	-0.369358	1.779081	0.122468	H	-0.293989	-0.704841	-0.888123
C	-1.064317	0.511438	0.680391	H	-2.986593	-2.918216	0.294524
C	-0.251732	-0.756629	0.212047	H	-2.157376	-2.515003	-1.197961
C	-2.468520	0.340859	0.126032	H	-1.192580	-2.096182	1.668853
C	-3.017672	-0.839729	-0.207802	H	-0.390723	-2.936816	0.357282
C	-2.332415	-2.168781	-0.168673	H	-3.799458	1.906166	0.906130
C	-0.996373	-2.057760	0.591335	H	3.204292	1.997577	-1.982961
C	-3.519301	1.423284	-0.035780	H	4.849665	1.900643	-1.340923
O	-4.709696	0.710102	-0.480759	H	3.640745	2.881959	-0.508053
C	-4.421585	-0.620898	-0.603888	H	5.182462	1.125553	1.010787
C	3.790246	1.947651	-1.059604	H	4.054685	0.037453	1.822361
C	4.117951	0.909598	1.169191	H	3.689412	1.761386	1.709140
C	1.565586	-0.902350	2.051074	H	0.804563	-1.537635	2.515513
O	-5.259524	-1.425696	-0.967047	H	2.527970	-1.385077	2.238615
O	3.779180	-0.561083	-2.315857	H	1.577552	0.045864	2.592485
O	-3.147678	2.363197	-0.996926	H	2.833292	-0.686134	-2.494595
C	-1.238223	0.665546	2.219876	H	-3.756791	3.119651	-0.953311
H	3.883848	-2.699431	-0.842336	H	-1.614463	-0.248414	2.688093
H	3.912566	-1.957847	0.744704	H	-0.303111	0.940252	2.709475
H	5.091852	-0.513423	-0.805078	H	-1.957319	1.464763	2.434003

Table S13. Cytotoxicity (ED_{50} μg/mL) of compounds **1**−**5**.

Compound	ED_{50} (μg/mL) for HCC Huh7 cells
**1**	> 50 μg/mL
**2**	> 50 μg/mL
**3**	> 50 μg/mL
**4**	> 50 μg/mL
**5**	> 50 μg/mL

Results are presented as mean ± SD. (n = 3).
